# Synthesis, Characterization, and Antifungal Activity of Novel Benzo[4,5]imidazo[1,2-*d*][1,2,4]triazine Derivatives

**DOI:** 10.3390/molecules23040746

**Published:** 2018-03-23

**Authors:** Ling-Xia Li, Jian Jiao, Xiao-Bin Wang, Min Chen, Xin-Can Fu, Wei-Jie Si, Chun-Long Yang

**Affiliations:** 1 College of Sciences, Nanjing Agricultural University, Nanjing 210095, China; 2014111007@njau.edu.cn (L.-X.L.); 2015111008@njau.edu.cn (J.J.); 2017211003@njau.edu.cn (X.-B.W.); chenmin@njau.edu.cn (M.C.); 2016811028@njau.edu.cn (X.-C.F.); 2015202048@njau.edu.cn (W.-J.S.); 2 Jiangsu Key Laboratory of Pesticide Science, Nanjing Agricultural University, Nanjing 210095, China; 3 Key Laboratory of Monitoring and Management of Crop Diseases and Pest Insects, Ministry of Agriculture, Nanjing Agricultural University, Nanjing 210095, China

**Keywords:** benzo[4,5]imidazo[1,2-*d*][1,2,4]triazine, synthesis, crystal structure, antifungal activity

## Abstract

A series of novel fused heterocyclic compounds bearing benzo[4,5]imidazo[1,2-*d*][1,2,4]triazine **4a**–**4w** were designed and conveniently synthesized via the intermediates 2-(halogenated alkyl)-1*H*-benzo[*d*]imidazoles **2a**, **2b**, and 2-((1-(substituted phenyl)hydrazinyl)alkyl)-1*H*-benzo[*d*]imidazoles **3a**–**3g**. The structures of all target compounds were characterized by FT-IR, ^1^H NMR, ^13^C NMR, and EI-MS, of which, the structure of compound **4n** was further determined by the single crystal X-ray diffraction. The crystal structure of **4n** was crystallized in the triclinic crystal system, space group *P*$\bar{1}$ with *a* = 9.033 (6) Å, *b* = 10.136 (7) Å, *c* = 10.396 (7) Å, *α* = 118.323 (7)°, *β* = 91.750 (8)°, *γ* = 104.198 (7)°, *Z* = 2, *V* = 800.2 (9) Å^3^; total *R* indices: *R*_1_ = 0.0475, *wR*_2_ = 0.1284. The antifungal activity of title compounds **4a**–**4w** in vitro against the phytopathogenic fungi *Botrytis cinerea* (*B. cinerea*), *Rhizoctonia solani* (*R. solani*) and *Colletotrichum capsici* (*C. capsici*) were evaluated, the bioassay results demonstrated that most of the title compounds exhibited obvious fungicidal activities at 50 μg/mL. This work indicated that benzo[4,5]imidazo[1,2-*d*][1,2,4]triazine derivatives could be considered as a new leading structure in searching for novel agricultural fungicides.

## 1. Introduction

Searching for novel fungicides with different structures is a promising way to overcome resistant fungi and plays a key role in preventing loss of agricultural output [1]. Over the past decades, efforts for developing highly efficient fungicides have been made by chemists. For example, some 1,4-pentadien-3-one [2], 1,2,3-triazole [3], quinazoline [4], quinazolin-4(3*H*)-one [5], 1,3,4-oxadiazole [6], coumarin [7], and tetronic acid [8] derivatives were well reported for their fungicidal activities against plant pathogenic fungi. However, their use as novel fungicides in agriculture was largely limited due to unsatisfactory curative rates, extremely complex synthetic routes, and structural instability in the farmland. Therefore, searching for the novel, eco-friendly, and highly efficient fungicides remains an urgent challenge in agriculture.

**Figure 1:**
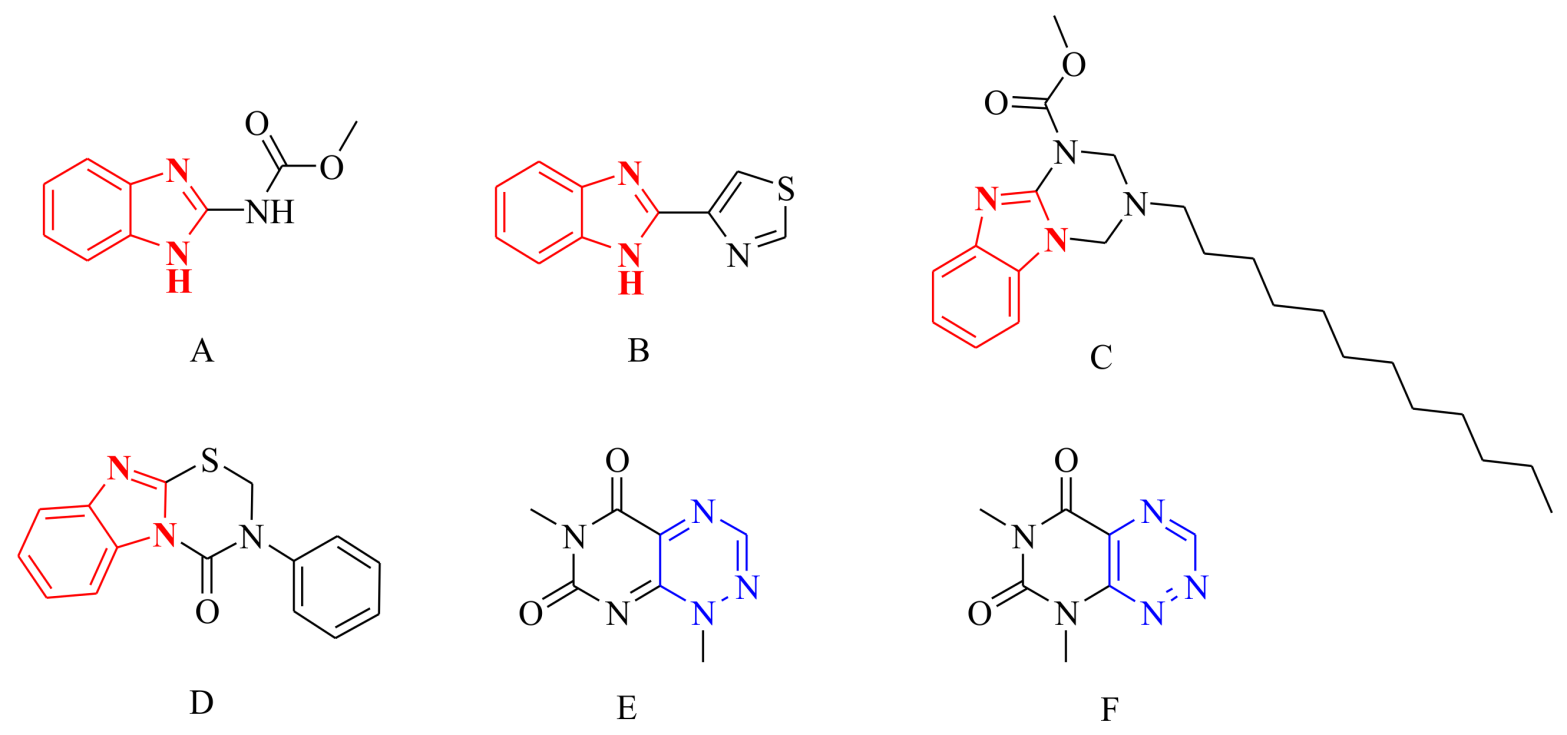
Compounds bearing a benzimidazole or triazine fragment. (**A**) Carbendazim; (**B**) Thiabendazole; (**C**) Hoe-22845; (**D**) 3-Phenyl-benzo[4,5]imidazo[2,1-*b*][1,3,5]thiadiazin-4-one; (**E**) Toxoflavin; (**F**) Fervenulin.

Benzimidazole derivatives have received considerable attention in medicinal and agrochemical chemistry due to their various bioactivities, such as antibacterial [9], anti-inflammatory [10], insecticidal [11], anti-cancer [12], and antiviral activities [13]. Some benzimidazole derivatives, such as carbendazim (Figure 1A), thiabendazole (Figure 1B), and Hoe-22845 (Figure 1C), were developed as high efficiency agricultural fungicides that made outstanding contributions for improving the yield and quality of crops [14]. Owing to their various antifungal activities, studies on the related synthesis and bioactivities of novel benzimidazole derivatives have attracted considerable attention [15,16]. Among these benzimidazole derivatives, the agricultural fungicide Hoe-22845 (Figure 1C) containing a unique benzo[4,5]imidazo[1,2-*a*][1,3,5]triazine substructure is interesting due to its broader antifungal spectra than other agricultural benzimidazole fungicides. Meanwhile, featuring a similar substructure, 3-phenyl-benzo[4,5]imidazo[2,1-*b*][1,3,5]thiadiazin-4-one (Figure 1D) was also reported for its wide and potent antifungal activity [17]. In addition, the 1,2,4-triazine fragment, an important scaffold of some natural products, including toxoflavin (Figure 1E) and fervenulin (Figure 1F) [18], has been widely studied due to its promising bioactivities, such as antifungal [19], anti-HIV [20], antimicrobial, and anti-cancer [21] activities. Recently, the antifungal activities of 1,2,4-triazines derivatives against *Candida albicans*, *Aspergillus niger*, and *Cryptococcus neoformans* were found by chemists [22]*.* Obviously, based on the structures of benzimidazole and triazine, further study to find novel effective fungicides is of great significance.

In continuation of our research on searching for novel agricultural fungicides, the above-mentioned active substructures in different compounds were comprehensively considered to design and synthesize a series of novel benzo[4,5]imidazo[1,2-*d*][1,2,4]triazine derivatives. The obtained target compounds were confirmed by FT-IR, ^1^H NMR, ^13^C NMR, EI-MS, and single crystal X-ray diffraction. Meanwhile, their agricultural antifungal activities against *Botrytis cinerea* (*B. cinerea*), *Rhizoctonia solani* (*R. solani*), and *Colletotrichum capsici* (*C. capsici*) were evaluated. To the best of our knowledge, this is the first report on the agricultural antifungal activity of benzo[4,5]imidazo[1,2-*d*][1,2,4]triazine derivatives. 

## 2. Results and Discussion

### 2.1. Synthesis Strategy for Intermediate ***3***

**Scheme 1:**
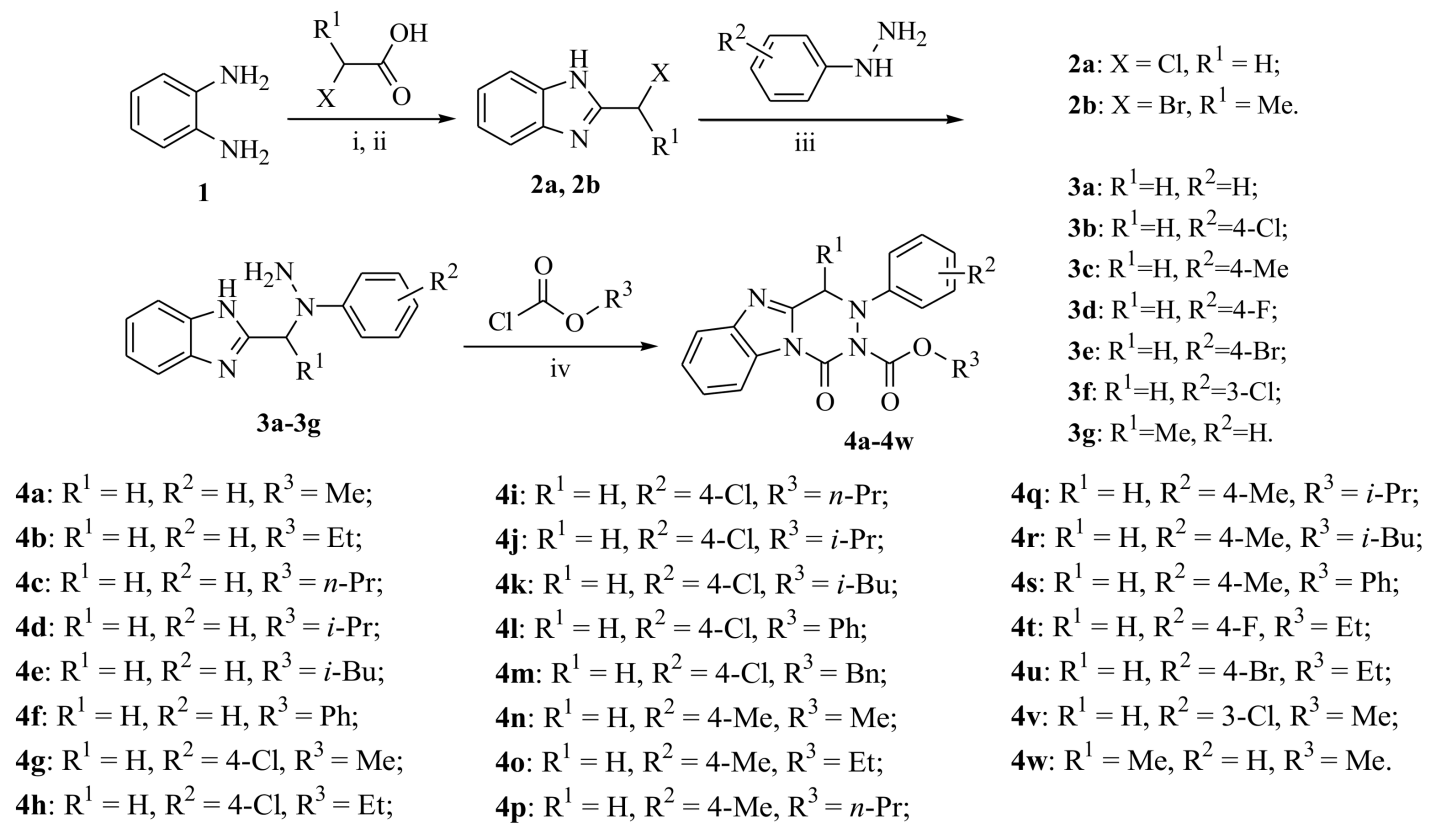
Synthetic route to the target compounds **4a**–**4w**. Reagents and conditions: (i) 2-Chloroacetic acid or 2-bromopropionic acid, 0.15 g/mL hydrochloric acid/r.t., reflux, 4 h; (ii) NaHCO_3_/r.t.; (iii) Substituted phenylhydrazines, Et_3_N, MeOH/reflux, 4 h; (iv) Et_3_N, chloroformates, THF/0–5 °C, 2–8 h.

The target compounds **4a**–**4w** and their intermediates **2a**, **2b,** and **3a**–**3g** were successfully achieved, and the synthetic route is illustrated in Scheme 1. The compounds **2a** and **2b** were synthesized by the reactions of 1,2-diaminobenzene with 2-chloroacetic acid or 2-bromopropionic acid in 70–74% yields, respectively. Then, using Et_3_N as catalyst, the yellow powders **3a**–**3g** were obtained in 51–64% yields by reaction of compound **2** with substituted phenylhydrazine in refluxing MeOH. 

**Scheme 2:**
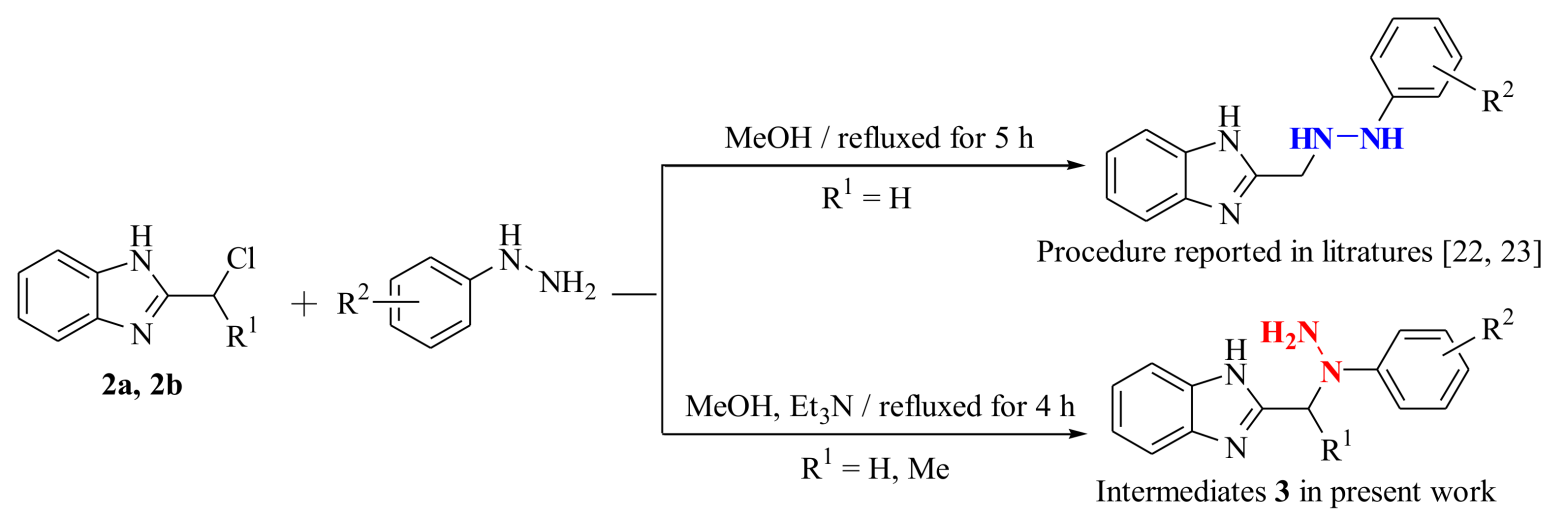
Two nucleophilic substitutions of intermediate **2** with substituted phenylhydrazines.

**Figure 2:**
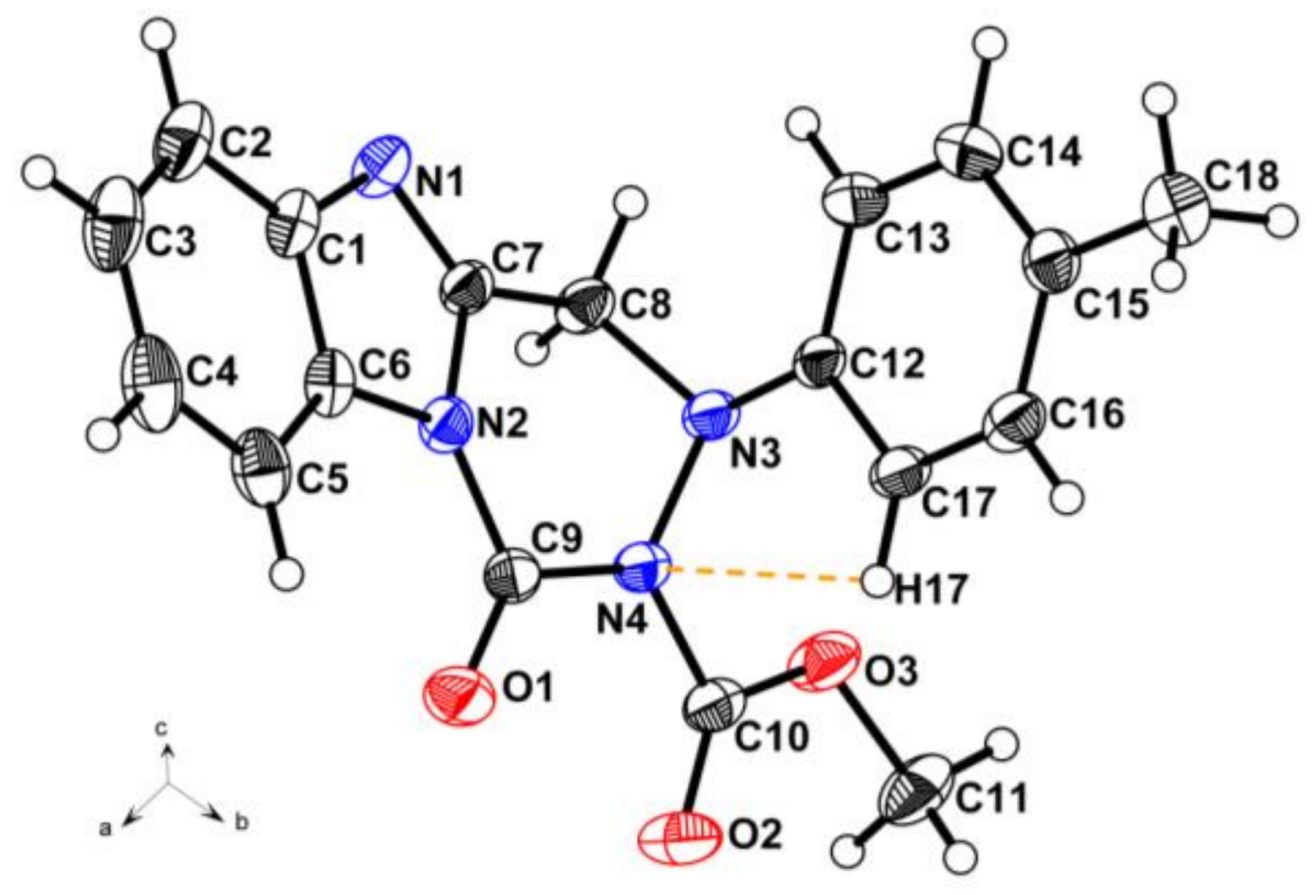
Crystal structure diagram of compound **4n** with intramolecular hydrogen bond.

According to the procedure reported in the literature [23,24], the nucleophilic substitution of intermediate **2** with substituted phenylhydrazines was carried out in refluxed methanol for 5 h to obtain 2-((2-(substituted phenyl)hydrazinyl)methyl)-1*H*-benzo[*d*]imidazole. However, in the present work, the intermediates **3a**–**3f** (2-((1-(substituted phenyl)hydrazinyl)methyl)-1*H*-benzo[*d*]imidazole) and **3g** (2-(1-(1-phenylhydrazinyl)ethyl)-1*H*-benzo[*d*]imidazole) were successfully prepared using similar materials in refluxed methanol containing Et_3_N for 4 h. It is an interesting phenomenon that using similar materials and procedures to permit different nucleophilic substitutions happened at different N-positions of the substituted phenylhydrazine groups (Scheme 2). Noteworthily, their only difference is the existence of excess Et_3_N as the catalyst. In the present work, Et_3_N was used as an acid binding agent to promote the nucleophilic substitution reaction at the secondary nitrogen position of the hydrazine group. This synthetic strategy in the present work is quite practical and plays a significant role in synthesis of the novel fused heterocycle benzo[4,5]imidazo[1,2-*d*][1,2,4]triazine derivatives. The above phenomenon was clearly confirmed by the crystal structure of compound **4n**. As illustrated in Figure 2, the presence of 4-methylphenyl group at N-3 position of compound **4n** indirectly proves the structural correctness of the compounds **3a**–**3g**.

### 2.2. Synthesis and Characterization of Title Compounds ***4***

The compounds **3a**–**3g** were reacted with different chloroformates to gain the target compounds **4a**–**4w** with 26–38% yields. All the synthesized compounds **4a**–**4w** were confirmed via IR, ^1^H NMR, ^13^C NMR, and EI-MS. The characteristic absorption at 1740–1790 cm^−^^1^ was attributed to the presence of C=O groups. In the ^1^H NMR spectra, the aromatic protons and the 1,2,4-triazine methylene protons of compounds **4a**–**4w** ranged 6.94–8.02 and 5.12–5.84 ppm, respectively. In the ^13^C NMR spectra, the absorption peaks at 150.24–153.56 and 113.83–149.01 ppm revealed the presences of C=O groups and aromatic carbons of compounds **4a**–**4w**. Meanwhile, the signals at 55.01–73.73 and 48.07–53.90 ppm signified the carbons in the 2-position of benzimidazole and the 6-position of 1,2,4-triazine, respectively. The mass spectral data of the target compounds **4a**–**4w** were consistent with their chemical structures. 

### 2.3. X-ray Crystal Structure of Compound ***4n***

**Figure 3:**
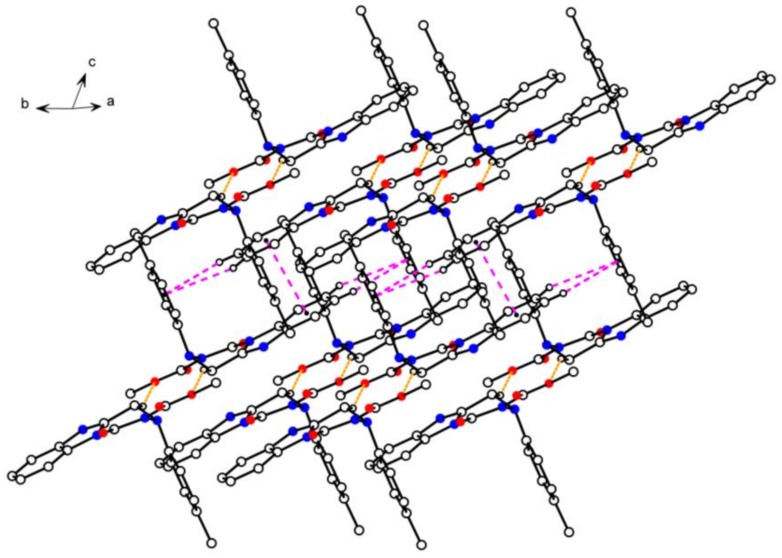
Crystal packing diagram of compound **4n**. Yellow dashed lines show intermolecular hydrogen bonds. Pink dashed lines show *π*···*π* interactions.

**Figure 4:**
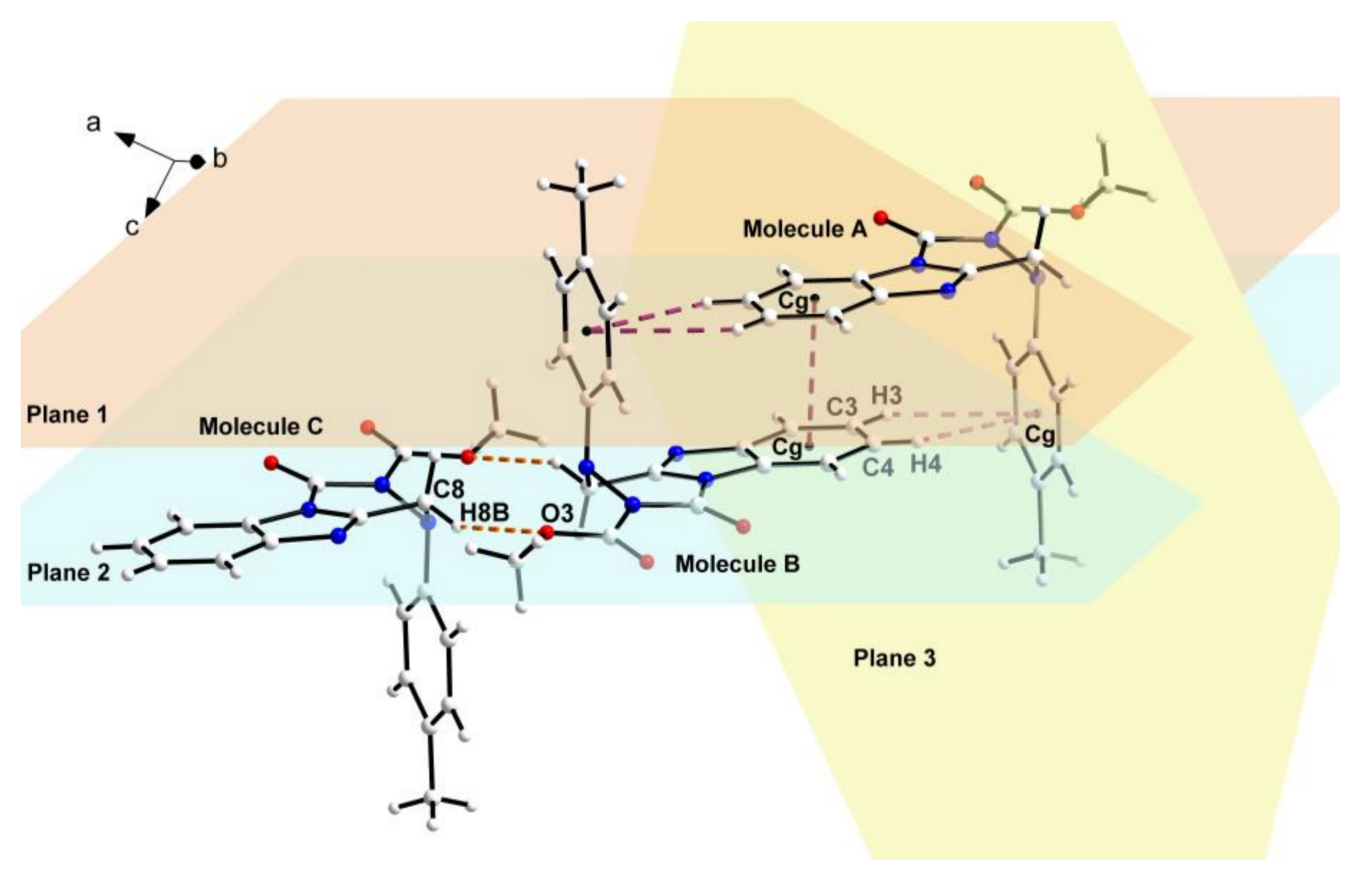
Partial crystal parking diagram of compound **4n**. Planes 1, 2, and 3 indicate respectively three planes containing benzimidazole ring of molecule A, benzimidazole ring of molecule B, and benzene ring of molecule A.

As a representative example, the structure of compound **4n** was further studied using single crystal X-ray analysis. The obtained crystal data was solved, refined, and is summarized in Table 1. The corresponding single crystal structure and crystal packing diagrams are shown by Figure 2, Figure 3 and Figure 4, respectively.

As shown in the crystal structure of compound **4n** (Figure 2), the aromatic benzimidazole and benzene rings and the nonaromatic hexatomic ring containing C7, C8, N2, N3, N4, and C9 atoms construct the skeletal structure of benzo[4,5]imidazo[1,2-*d*][1,2,4]triazine derivatives. In the single crystal structure, the intramolecular hydrogen bond C17–H17···N4 combines with the N3 and C12 atoms to form a latent penta-heterocycle. In the above penta-heterocycle, the angle of C17–H17···N4 and the distance between H17 and N4 were 100° and 2.450 Å, respectively. Meanwhile, owing to the electron delocalization, the single bonds N1–C1 (1.410(2) Å), N2–C6 (1.406(2) Å), N2–C7 (1.401(2) Å), N2–C9 (1.402(2) Å), N4–C9 (1.408(2) Å) and N4–C10 (1.417(2) Å) were obviously shorter than the normal bond N–C (1.47–1.50 Å), and the double bond C7=N1 (1.293(2) Å) was also shorter than the normal bond C=N (1.34–1.38 Å). The relevant data of all the bond lengths and angles in the crystal structure of compound **4n** are listed in the Supporting Information.

As shown in the crystal parking diagram of compound **4n** (Figure 3), the space network structure with many cavities was obviously constituted by the intermolecular hydrogen bond (C8–H8B···O3) and the intermolecular *π*–*π* stacking interactions. In order to further understand the crystal structure of the title compounds, the partial crystal parking diagram of **4n** containing three single molecules were obtained and are shown in Figure 4. In this Figure, planes 1, 2, and 3 indicate three planes containing the benzimidazole ring of molecule A, the benzimidazole ring of molecule B, and the benzene ring of molecule A, respectively. The Cg means the centers of all the benzene rings in the single crystal structure of compound **4n**. As shown in Figure 4, molecules B and C were connected by the intermolecular hydrogen bond C8–H8B···O3, and the molecules A and B were combined by the two kinds of *π*–*π* stacking interactions. These *π*–*π* stacking interactions contain an offset face-to-face stacking interaction between two benzimidazole rings and an edge-to-face stacking interaction between benzimidazole and benzene rings in different molecules. In the face-to-face stacking interaction, the centroid-centroid and centroid-plane distances were 3.5279(18) Å and 3.3624(4) Å between the parallel Planes 1 and 2, respectively, which indicates a strong *π*–*π* stacking interactions existing in the crystal structure of compound **4n**. In the edge-to-face stacking interaction, the distances of C3···Cg, H3···Cg, C4···Cg, and H4···Cg were 3.7844(33) Å, 3.0998(17) Å, 3.8439(29) Å, and 3.2161(14) Å, respectively. Moreover, the angles of C3–H3···Cg and C4–H4···Cg were 131.905(150)° and 126.606(126)°, respectively.

### 2.4. Antifungal Activity

Using the mycelium growth rate method on PSA (potato sucrose agar) medium [25], the inhibition effects of title compounds **4a**–**4w** against the phytopathogenic fungi including *B. cinerea*, *R. solani*, and *C. capsici* were tested in vitro at 50 μg/mL. The agricultural fungicide carbendazim was used as the standard antifungal reference. Antifungal results in Table 2 indicated that compounds **4a**, **4g**–**4o**, **4q**, **4u**, and **4v** shown good activities toward *B. cinerea* with inhibitory rates of 62.1%, 70.5%, 68.6%, 68.4%, 51.5%, 50.8%, 50.5%, 50.8%, 69.2%, 76.7%, 52.1%, 64.4% and 62.1%, respectively. The compounds **4b**, **4f**–**4i**, **4n**–**4p**, and **4s**–**4v** obviously inhibited the mycelium growth of *R. solani*, with inhibitory rates of 63.5%, 57.3%, 58.0%, 53.7%, 50.6%, 53.9%, 57.8%, 52.8%, 61.4%, 50.2%, 60.6% and 61.1%, respectively. The mycelium growth inhibition rates of compounds **4a**, **4g**, **4l**, **4n**, and **4t**–**4v** against *C. capsici* reached respectively 52.1%, 60.3%, 57.8%, 58.9%, 66.8%, 51.7% and 60.1%. Noteworthily, the inhibitory rates of compounds **4g**, **4n**, **4u**, and **4v** against the above all tested fungi exceeded 50%. Meanwhile, compounds **4o** against *B. cinerea*, **4b** against *R. solani,* and **4t** against *C. capsici* showed the highest antifungal activities with inhibitory rates of 76.7%, 63.5%, and 66.8%, respectively.

### 2.5. Structure–Activity Relationships

Most of the title compounds showed obvious antifungal activities against *B. cinerea*, *R. solani,* and *C. capsici*, and some structure–activity relationships can be analyzed and summarized. First, the presence of electron-withdrawing groups, such as fluorine, chlorine, and bromine atoms, at R^2^ position contributes to the enhancement of antifungal activities. For example, the inhibitory rates of compound **4g** against *B. cinerea*, *R. solani,* and *C. capsici* were 70.5%, 58.0%, and 60.3%, respectively, which were better than that of compounds **4a** (62.1%, 32.0%, and 52.1%) and **4n** (69.2%, 53.9%, and 58.9%). Second, the introduction of small alkyls, such as methyl, ethyl, *n*-propyl, and *i*-propyl groups at the R^3^ position plays a positive effect on the antifungal activities of title compounds. For instance, compounds **4g** (R^3^ = Me), **4h** (R^3^ = Et), **4i** (R^3^ = *n*-Pr), and **4j** (R^3^ = *i*-Pr) shown higher activities than **4k** (R^3^ = *i*-Bu), **4l** (R^3^ = Ph), and **4m** (R^3^ = Bn) against almost all tested phytopathogenic fungi. Third, a methyl group at the R^1^ position of the title compounds weakened their antifungal activities*.* For example, the inhibitory rates of compound **4a** against *B. cinerea*, *R. solani,* and *C. capsici* were respectively 62.1, 32.0, and 52.1%, which were higher than that of the compounds **4w** (45.9, 28.1, and 26.6%, respectively).

## 3. Experimental Section

### 3.1. General

All melting points of the title compounds were determined on an uncorrected WRS-1B digital melting point apparatus (Jingmi Science, Shanghai, China). ^1^H NMR and ^13^C NMR spectra were measured on Bruker 400 spectrometer (Bruker, Karlsruhe, Germany) using DMSO-*d*_6_ as solvent and TMS as internal standard. Mass spectra were recorded on a TRACE 2000 spectrometer (Finnigan, Silicon Valley, CA, USA). The compound samples prepared with KBr disk were gauged on a Thermo Nicolet 380 FT-IR spectrometer to obtain the corresponding infrared spectra. Progress of the reactions was monitored by thin layer chromatography on silica gel GF_254_ (Yuhua, Gongyi, China). All reagents and solvents purchased from commercial suppliers were analytically or chemically pure and were not further purified. 

### 3.2. Chemistry

#### 3.2.1. General Procedure for Synthesis of Compounds **2**

The intermediate **2a** was synthesized according to the literature [16] with minor modification. The mixture of *o*-phenylenediamine (20 mmol) and chloroacetic acid (22 mmol) in 0.15 g/mL hydrochloric acid (120 mL) was refluxed under 108 °C for 4 h. Then, the obtained mixture was cooled to room temperature and neutralized with sodium bicarbonate until the pH value was over 7.0 to generate yellow solid **2a**. The 2-(chloromethyl)-1*H*-benzo[*d*]imidazole **2a** was obtained with the 70.3% yield, m.p. 147.6 °C (Ref. 146–148 °C [16]). 

Using the similar method, *o*-phenylenediamine was reacted with 2-bromopropionic acid to obtain the intermediate **2b**. *2-(1-Bromoethyl)-1H-benzo[d]imidazole* (**2b**): white powder; yield 73.7%; m.p. 130.8–132.4 °C; IR (KBr, cm^−1^) *v*: 2982, 2818, 2731, 1430, 1272, 1226, 745, 690, 617; ^1^H NMR (400 MHz, DMSO-*d*_6_) *δ* (ppm): 10.17 (s, 1H), 6.74 (dd, *J* = 17.2, 7.7 Hz, 2H), 6.67 (d, *J* = 7.3 Hz, 1H), 6.59 (d, *J* = 7.5 Hz, 1H), 3.76 (q, *J* = 5.3 Hz, 1H), 1.24 (d, *J* = 6.6 Hz, 3H); ^13^C NMR (100 MHz, DMSO-*d*_6_) *δ* (ppm): 153.73, 138.72, 122.79, 115.85, 114.69, 51.76, 23.54.

#### 3.2.2. General Procedure for Synthesis of Compounds **3**

Triethylamine (6.3 mL, 45 mmol) was added to a mixture of compounds **2a** (5.0 g, 30 mmol) and substituted hydrazinobenzene (33 mmol) in methanol (50 mL). After refluxing for 4 h, the mixture was poured into cooled water, and then precipitated to obtain the yellow solids **3a**–**3f**. Using the same procedure and intermediate **2b** as a material, the yellow solid **3g** was synthesized with 60.7% yield.

*2-((1-Phenylhydrazinyl)methyl)-1H-benzo[d]imidazole* (**3a**): yellow powder; yield 51.4%; m.p. 138.8–139.9 °C; IR (KBr, cm^−1^) *v*: 3255, 3146, 3055, 1596, 1493, 1420, 1198, 767, 738, 689; ^1^H NMR (400 MHz, DMSO-*d*_6_) *δ* (ppm): 12.37 (br, 1H), 7.50 (s, 2H), 7.21–7.09 (m, 4H), 7.06 (d, *J* = 8.0 Hz, 2H), 6.65 (t, *J* = 6.7 Hz, 1H), 4.82 (s, 2H), 4.79 (br, 2H); ^13^C NMR (100 MHz, DMSO-*d*_6_) *δ* (ppm): 152.81, 151.99, 129.08, 121.89, 117.80, 113.50, 53.80.

*2-((1-(4-Chlorophenyl)hydrazinyl)methyl)-1H-benzo[d]imidazole* (**3b**): yellow powder; yield 60.2%; m.p. 246.6–247.8 °C; IR (KBr, cm^−1^) *v*: 3319, 3054, 2995, 1592, 1491, 1422, 1032, 1023, 857, 807, 739, 747; ^1^H NMR (400 MHz, DMSO-*d*_6_) *δ* (ppm): 12.39 (br, 1H), 7.51 (s, 2H), 7.30–7.06 (m, 6H), 4.86 (br, 2H), 4.84 (s, 2H); ^13^C NMR (100 MHz, DMSO-*d*_6_) *δ* (ppm): 152.28, 150.86, 128.70, 121.96, 121.27, 114.98, 53.63.

*2-((1-(4-Tolyl)hydrazinyl)methyl)-1H-benzo[d]imidazole* (**3c**): yellow powder; yield 63.6%; m.p. 157.2–159.1 °C; IR (KBr, cm^−1^) *v*: 3313, 3029, 2915, 1513, 1424, 1310, 899, 805, 745; ^1^H NMR (400 MHz, DMSO-*d*_6_) *δ* (ppm): 12.16 (br, 1H), 7.49 (dd, *J* = 5.0, 3.2 Hz, 2H), 7.12 (dd, *J* = 5.9, 3.1 Hz, 2H), 6.96 (q, *J* = 8.8 Hz, 4H), 4.78 (br, 2H), 4.77 (s, 2H), 2.15 (s, 3H); ^13^C NMR (100 MHz, DMSO-*d*_6_) *δ* (ppm): 152.85, 150.01, 129.51, 126.54, 121.84, 113.93, 54.14, 20.45.

*2-((1-(4-Fluorophenyl)hydrazinyl)methyl)-1H-benzo[d]imidazole* (**3d**): yellow powder; yield 58.7%; m.p. 246.0–247.4 °C; IR (KBr, cm^−1^) *v*: 3349, 3047, 2982, 1503, 1430, 1216, 821, 746, 699; ^1^H NMR (400 MHz, DMSO-*d*_6_) *δ* (ppm): 12.55 (s, 1H), 7.50 (s, 2H), 7.13 (dd, *J* = 5.8, 3.1 Hz, 2H), 7.07 (dd, *J* = 8.9, 4.6 Hz, 2H), 6.98 (t, *J* = 8.8 Hz, 2H), 4.81 (br, 2H), 4.78 (s, 2H); ^13^C NMR (100 MHz, DMSO-*d*_6_) *δ* (ppm): 152.52, 148.87, 121.94, 115.84, 115.52, 115.16, 54.34.

*2-((1-(4-Bromophenyl)hydrazinyl)methyl)-1H-benzo[d]imidazole* (**3e**): yellow powder; yield 55.4%; m.p. 225.7–227.7 °C; IR (KBr, cm^−1^) *v*: 3221, 3060, 2914, 1590, 1487, 1451, 1437, 1319, 1023, 804, 747; ^1^H NMR (400 MHz, DMSO-*d*_6_) *δ* (ppm): 12.28 (s, 1H), 7.51 (s, 2H), 7.27 (d, *J* = 8.7 Hz, 2H), 7.13 (dd, *J* = 5.8, 3.1 Hz, 2H), 7.01 (d, *J* = 8.8 Hz, 2H), 4.90 (s, 2H), 4.84 (s, 2H); ^13^C NMR (100 MHz, DMSO-*d*_6_) *δ* (ppm): 152.19, 151.17, 131.51, 121.99, 115.50, 108.81, 53.48.

*2-((1-(3-Chlorophenyl)hydrazinyl)methyl)-1H-benzo[d]imidazole* (**3f**): yellow powder; yield 60.8%; m.p. 253.6–255.4 °C; IR (KBr, cm^−1^) *v*: 3334, 2983, 2605, 1590, 1495, 1420, 1364, 1271, 990, 857, 739, 687; ^1^H NMR (400 MHz, DMSO-*d*_6_) *δ* (ppm): 12.30 (s, 1H), 7.50 (s, 2H), 7.14–7.10 (m, 4H), 6.94 (d, *J* = 8.4 Hz, 1H), 6.65 (d, *J* = 7.7 Hz, 1H), 4.85 (s, 2H), 4.81 (br, 2H); ^13^C NMR (100 MHz, DMSO-*d*_6_) *δ* (ppm): 153.26, 152.17, 134.01, 130.53, 121.96, 116.90, 112.92, 111.61, 53.31.

*2-(1-(1-Phenylhydrazinyl)ethyl)-1H-benzo[d]imidazole* (**3g**): yellow powder; yield 60.7%; m.p. 232.9–233.4 °C; IR (KBr, cm^−1^) *v*: 3325, 2869, 2737, 1589, 1480, 1435, 1416, 1325, 876, 811, 759, 746, 677; ^1^H NMR (400 MHz, DMSO-*d*_6_) *δ* (ppm): 12.18 (s, 1H), 7.49 (s, 2H), 7.28–7.01 (m, 6H), 6.68 (t, *J* = 6.5 Hz, 1H), 5.32 (q, *J* = 6.6 Hz, 1H), 4.30 (br, 2H), 1.61 (d, *J* = 6.7 Hz, 3H); ^13^C NMR (101 MHz, DMSO-*d*_6_) *δ*(ppm): 156.22, 151.97, 129.11, 121.79, 118.04, 114.14, 54.39, 15.89.

#### 3.2.3. General Procedure for Synthesis of Compounds **4**

The chloroformates (8.2 mmol) were added dropwise to a mixture of compound **3** (4 mmol), Et_3_N (1.8 mL, 13 mmol) and tetrahydrofuran (10 mL) at 0 °C. Then, the obtained mixture was stirred at 0–5 °C for 2–8 h and monitored by thin layer chromatography on silica gel GF_254_ using the mixture of ethyl acetate and petroleum ether (*V*:*V* = 1:1). After completion of the reaction, the mixture was filtered, concentrated in vacuo and purified on a silica gel column to afford the title compounds **4.**

*Methyl 1-oxo-3-phenyl-3*,*4-dihydrobenzo[4,5]imidazo[1,2-d][1,2,4]triazine-2(1H)-carboxylate* (**4a**): white powder; yield 33.3%; m.p. 161.2–163.4 °C; IR (KBr, cm^−1^) *v*: 1745, 1565, 1498, 1442; ^1^H NMR (400 MHz, DMSO-*d*_6_) *δ* (ppm): 8.00–7.93 (m, 1H), 7.72–7.65 (m, 1H), 7.41–7.34 (m, 2H), 7.29 (dd, *J* = 8.7, 7.4 Hz, 2H), 7.10 (d, *J* = 8.0 Hz, 2H), 6.95 (t, *J* = 7.3 Hz, 1H), 5.57 (s, 1H), 5.18 (s, 1H), 3.91 (s, 3H); ^13^C NMR (100 MHz, DMSO-*d*_6_) *δ* (ppm): 152.10, 151.08, 147.47, 145.58, 142.76, 131.22, 130.19, 125.69, 123.11, 120.35, 115.26, 114.40, 55.06, 48.20; EI-MS (*m*/*z*): 322, found 322 [M]^+^.

*Ethyl 1-oxo-3-phenyl-3,4-dihydrobenzo[4,5]imidazo[1,2-d][1,2,4]triazine-2(1H)-carboxylate* (**4b**): white powder; yield 32.1%; m.p. 177.1–179.3 °C; IR (KBr, cm^−1^) *v*: 1740, 1568, 1497, 1451; ^1^H NMR (400 MHz, DMSO-*d*_6_) *δ* (ppm): 8.00–7.93 (m, 1H), 7.72–7.65 (m, 1H), 7.41–7.34 (m, 2H), 7.29 (t, *J* = 8.0 Hz, 2H), 7.10 (d, *J* = 8.0 Hz, 2H), 6.95 (t, *J* = 7.3 Hz, 1H), 5.57 (s, 1H), 5.18 (s, 1H), 4.36 (q, *J* = 7.1 Hz, 2H), 1.30 (t, *J* = 7.1 Hz, 3H); ^13^C NMR (100 MHz, DMSO-*d*_6_) *δ* (ppm): 151.54, 151.13, 147.51, 145.62, 142.77, 131.23, 130.19, 125.66, 123.07, 120.33, 115.22, 114.41, 64.29, 48.16, 14.58; EI-MS (*m*/*z*): 336, found 336 [M]^+^.

*Propyl 1-oxo-3-phenyl-dihydrobenzo[4,5]imidazo[1,2-d][1,2,4]triazine-2(1H)-carboxylate* (**4c**): white powder; yield 34.2%; m.p. 161.4–162.9 °C; IR (KBr, cm^−1^) *v*: 1788, 1709, 1624, 1596; ^1^H NMR (400 MHz, DMSO-*d*_6_) *δ* (ppm): 7.96 (d, *J* = 8.0 Hz, 1H), 7.67 (d, *J* = 7.0 Hz, 1H), 7.44–7.33 (m, 2H), 7.29 (t, *J* = 7.6 Hz, 2H), 7.07 (d, *J* = 8.0 Hz, 2H), 6.94 (t, *J* = 7.3 Hz, 1H), 5.55 (s, 1H), 5.17 (s, 1H), 4.28 (t, *J* = 6.4 Hz, 2H), 1.76–1.62 (m, 2H), 0.92 (t, *J* = 7.4 Hz, 3H); ^13^C NMR (100 MHz, DMSO-*d*_6_) *δ* (ppm): 151.59, 151.15, 147.50, 145.64, 131.23, 130.17, 125.65, 123.06, 120.31, 115.20, 114.42, 69.51, 48.19, 21.99, 10.58; EI-MS (*m*/*z*): 350, found, 350 [M]^+^.

*Isopropyl 1-oxo-3-phenyl-3,4-dihydrobenzo[4,5]imidazo[1,2-d][1,2,4]triazine-2(1H)-carboxylate* (**4d**): yellow powder; yield 31.3%; m.p. 199.1–200.5 °C; IR (KBr, cm^−1^) *v*: 1757, 1731, 1570, 1497; ^1^H NMR (400 MHz, DMSO-*d*_6_) *δ* (ppm): 7.95 (d, *J* = 7.0 Hz, 1H), 7.67 (d, *J* = 7.3 Hz, 1H), 7.42–7.33 (m, 2H), 7.28 (t, *J* = 7.6 Hz, 2H), 7.05 (d, *J* = 8.1 Hz, 2H), 6.94 (t, *J* = 7.1 Hz, 1H), 5.53 (s, 1H), 5.18 (s, 1H), 5.11 (dt, *J* = 12.3, 6.1 Hz, 1H), 1.32 (d, *J* = 3.3 Hz, 6H); ^13^C NMR (100 MHz, DMSO-*d*_6_) *δ* (ppm): 151.10, 147.54, 145.64, 142.77, 131.24, 130.16, 125.62, 123.01, 120.30, 115.17, 114.41, 72.61, 48.14, 22.00; EI-MS (*m*/*z*): 350, found, 350 [M]^+^.

*Isobutyl 1-oxo-3-phenyl-3,4-dihydrobenzo[4,5]imidazo[1,2-d][1,2,4]triazine-2(1H)-carboxylate* (**4e**): white powder; yield 30.3%; m.p. 171.3–174.5 °C; IR (KBr, cm^−1^) *v*: 1743, 1572, 1496, 1448; ^1^H NMR (400 MHz, DMSO-*d*_6_) *δ* (ppm): 7.97 (d, *J* = 6.7 Hz, 1H), 7.68 (d, *J* = 7.6 Hz, 1H), 7.42–7.33 (m, 2H), 7.29 (t, *J* = 7.8 Hz, 2H), 7.08 (d, *J* = 8.1 Hz, 2H), 6.94 (t, *J* = 7.2 Hz, 1H), 5.57 (s, 1H), 5.18 (s, 1H), 4.12 (d, *J* = 6.2 Hz, 2H), 2.06–1.87 (m, 1H), 0.92 (d, *J* = 6.6 Hz, 6H); ^13^C NMR (100 MHz, DMSO-*d*_6_) *δ* (ppm): 151.59, 151.14, 147.52, 145.66, 142.80, 131.25, 130.16, 125.65, 123.07, 120.32, 115.22, 114.45, 73.63, 48.27, 27.83, 19.09; EI-MS (*m*/*z*): 364, found 364 [M]^+^.

*Phenyl 1-oxo-3-phenyl-3*,*4-dihydrobenzo[4,5]imidazo[1,2-d][1,2,4]triazine-2(1H)-carboxylate* (**4f**): yellow powder; yield 37.3%; m.p. 185.7–189.2 °C; IR (KBr, cm^−1^) *v*: 1750, 1567, 1496, 1485; ^1^H NMR (400 MHz, DMSO-*d*_6_) *δ* (ppm): 7.98 (d, *J* = 7.3 Hz, 1H), 7.71 (d, *J* = 7.6 Hz, 1H), 7.49 (q, *J* = 7.7 Hz, 2H), 7.43–7.38 (m, 2H), 7.37–7.28 (m, 5H), 7.21 (d, *J* = 8.5 Hz, 2H), 6.98 (t, *J* = 7.2 Hz, 1H), 5.64 (s, 1H), 5.33 (s, 1H); ^13^C NMR (100 MHz, DMSO-*d*_6_) *δ* (ppm): 151.09, 150.73, 150.41, 147.31, 145.43, 142.79, 131.20, 130.29, 130.20, 127.03, 125.86, 123.30, 122.07, 121.75, 120.45, 115.34, 114.46, 48.09; EI-MS (*m*/*z*): 384, found 384 [M]^+^.

*Methyl 1-oxo-3-(4-chlorophenyl)-3,4-dihydrobenzo[4,5]imidazo[1,2-d][1,2,4]triazine-2(1H)-carboxylate* (**4g**): yellow powder; yield 32.3%; m.p. 144.5–146.8 °C; IR (KBr, cm^−1^) *v*: 1789, 1613, 1559, 1490; ^1^H NMR (400 MHz, DMSO-*d*_6_) *δ* (ppm): 7.99–7.90 (m, 1H), 7.73–7.66 (m, 1H), 7.44–7.37 (m, 2H), 7.34 (d, *J* = 9.1 Hz, 2H), 7.13 (d, *J* = 9.1 Hz, 2H), 5.57 (s, 1H), 5.18 (s, 1H), 3.91 (s, 3H); ^13^C NMR (100 MHz, DMSO-*d*_6_) *δ* (ppm): 151.97, 150.86, 146.44, 145.33, 142.75, 131.23, 129.98, 127.20, 125.74, 120.37, 117.14, 114.43, 60.24, 55.13, 48.22; EI-MS (*m*/*z*): 356, found 356 [M]^+^.

*Ethyl 1-oxo-3-(4-chlorophenyl)-3,4-dihydrobenzo[4,5]imidazo[1,2-d][1,2,4]triazine-2(1H)-carboxylate* (**4h**): yellow powder; yield 34.8%; m.p. 162.1–163.8 °C; IR (KBr, cm^−1^) *v*: 1790, 1747, 1570, 1492; ^1^H NMR (400 MHz, DMSO-*d*_6_) *δ* (ppm): 8.02–7.92 (m, 1H), 7.73–7.66 (m, 1H), 7.44–7.30 (m, 4H), 7.12 (d, *J* = 9.0 Hz, 2H), 5.58 (s, 1H), 5.18 (s, 1H), 4.37 (q, *J* = 7.0 Hz, 2H), 1.31 (t, *J* = 7.1 Hz, 3H); ^13^C NMR (100 MHz, DMSO-*d*_6_) *δ* (ppm): 151.39, 150.90, 146.47, 145.37, 142.76, 131.22, 129.99, 127.13, 125.73, 120.37, 117.09, 114.43, 64.41, 48.19, 14.56; EI-MS (*m*/*z*): 370, found 370 [M]^+^.

*Propyl 1-oxo-3-(4-chlorophenyl)-3,4-dihydrobenzo[4,5]imidazo[1,2-d][1,2,4]triazine-2(1H)-carboxylate* (**4i**): yellow powder; yield 34.1%; m.p. 150.3–152.1 °C; IR (KBr, cm^−1^) *v*: 1785, 1711, 1596, 1574; ^1^H NMR (400 MHz, DMSO-*d*_6_) *δ* (ppm): 8.00–7.94 (m, 1H), 7.70 (dd, *J* = 6.3, 2.4 Hz, 1H), 7.43–7.37 (m, 2H), 7.37–7.31 (m, 2H), 7.13 (dd, *J* = 9.7, 2.7 Hz, 2H), 5.59 (s, 1H), 5.19 (s, 1H), 4.29 (t, *J* = 6.5 Hz, 2H), 1.77–1.60 (m, 2H), 0.93 (t, *J* = 7.4 Hz, 3H); ^13^C NMR (100 MHz, DMSO-*d*_6_) *δ* (ppm): 151.44, 150.91, 146.48, 145.39, 142.77, 131.23, 129.98, 127.12, 125.72, 120.36, 117.08, 114.46, 69.63, 48.24, 21.98, 10.59; EI-MS (*m*/*z*): 384, found 384 [M]^+^.

*Isopropyl* 1*-oxo-3-(4-chlorophenyl)-3,4-dihydrobenzo[4,5]imidazo[1,2-d][1,2,4]triazine-2(1H)-carboxylate* (**4j**): white powder; yield 35.3%; m.p. 165.5–168.3 °C; IR (KBr, cm^−1^) *v*: 1787, 1713, 1569, 1494; ^1^H NMR (400 MHz, DMSO-*d*_6_) *δ* (ppm): 7.98–7.93 (m, 1H), 7.73–7.63 (m, 1H), 7.43–7.37 (m, 2H), 7.34 (d, *J* = 8.8 Hz, 2H), 7.09 (d, *J* = 8.9 Hz, 2H), 5.56 (s, 1H), 5.14 (s, 1H), 5.15–5.02 (m, 1H), 1.33 (d, *J* = 3.2 Hz, 6H); ^13^C NMR (100 MHz, DMSO-*d*_6_) *δ* (ppm): 150.91, 146.51, 145.40, 142.77, 131.23, 129.99, 127.09, 125.70, 120.35, 117.05, 114.45, 72.78, 48.21, 22.00; EI-MS (*m*/*z*): 384, found 384 [M]^+^.

*Isobutyl 1-oxo-3-(4-chlorophenyl)-3*,*4-dihydrobenzo[4,5]imidazo[1,2-d][1,2,4]triazine-2(1H)-carboxylate* (**4k**): white powder; yield 32.3%; m.p.158.0–159.7 °C; IR (KBr, cm^−1^) *v*: 1786, 1707, 1619, 1573; ^1^H NMR (400 MHz, DMSO-*d*_6_) *δ* (ppm): 8.00–7.93 (m, 1H), 7.72–7.65 (m, 1H), 7.43–7.37 (m, 2H), 7.35 (d, *J* = 7.9 Hz, 2H), 7.11 (d, *J* = 8.9 Hz, 2H), 5.57 (s, 1H), 5.17(s, 1H), 4.12 (d, *J* = 6.3 Hz, 2H), 1.98 (tq, *J* = 12.7, 6.3 Hz, 1H), 0.93 (d, *J* = 6.7 Hz, 6H); ^13^C NMR (100 MHz, DMSO-*d*_6_) *δ* (ppm): 151.43, 150.90, 146.49, 145.41, 142.78, 131.24, 129.96, 127.13, 125.69, 120.34, 117.08, 114.48, 73.73, 48.30, 27.80, 19.08; EI-MS (*m*/*z*): 398, found 398 [M]^+^.

*Phenyl 1-oxo-3-(4-chlorophenyl)-3,4-dihydrobenzo[4,5]imidazo[1,2-d][1,2,4]triazine-2(1H)-carboxylate* (**4l**): yellow powder; yield 36.0%; m.p. 175.5–177.6 °C; IR (KBr, cm^−1^) *v*: 1751, 1570, 1491, 1450; ^1^H NMR (400 MHz, DMSO-*d*_6_) *δ* (ppm): 8.06–7.94 (m, 1H), 7.74 (d, *J* = 6.8 Hz, 1H), 7.52 (t, *J* = 7.8 Hz, 2H), 7.41 (dt, *J* = 17.1, 7.6 Hz, 7H), 7.29 (d, *J* = 9.1 Hz, 2H), 5.68 (s, 1H), 5.37 (s, 1H); ^13^C NMR (100 MHz, DMSO-*d*_6_) *δ* (ppm): 150.76, 150.24, 146.25, 145.17, 142.76, 131.19, 130.17, 127.37, 127.05, 125.90, 122.05, 120.47, 117.25, 114.49, 48.13; EI-MS (*m*/*z*): 418, found 418 [M]^+^.

*Benzyl 1-oxo-3-(4-chlorophenyl)-3,4-dihydrobenzo[4,5]imidazo[1,2-d][1,2,4]triazine-2(1H)-carboxylate* (**4m**): white powder; yield 33.7%; m.p. 158.5–161.2 °C; IR (KBr, cm^−1^) *v*: 1747, 1567, 1492, 1449; ^1^H NMR (400 MHz, DMSO-*d*_6_) *δ* (ppm): 7.97 (dd, *J* = 6.3, 2.6 Hz, 1H), 7.70 (dd, *J* = 6.2, 2.5 Hz, 1H), 7.51–7.27 (m, 9H), 7.14 (d, *J* = 9.1 Hz, 2H), 5.59 (s, 1H), 5.42 (s, 2H), 5.20 (s, 1H); ^13^C NMR (100 MHz, DMSO-*d*_6_) *δ* (ppm): 151.40, 150.90, 146.43, 145.35, 142.77, 135.66, 131.21, 129.98, 128.97, 128.78, 128.27, 127.21, 125.76, 120.38, 117.16, 114.48, 69.33, 48.25; EI-MS (*m*/*z*): 432, found 432 [M]^+^.

*Methyl 1-oxo-3-(4-tolyl)-3,4-dihydrobenzo[4,5]imidazo[1,2-d][1,2,4]triazine-2(1H)-carboxylate* (**4n**): white powder; yield 37.3%; m.p. 171.0–173.2 °C; IR (KBr, cm^−1^) *v*: 1792, 1574, 1508, 1454; ^1^H NMR (400 MHz, DMSO-*d*_6_) *δ* (ppm): 7.95 (d, *J* = 7.8 Hz, 1H), 7.68 (d, *J* = 7.0 Hz, 1H), 7.38 (p, *J* = 7.3 Hz, 2H), 7.08 (d, *J* = 8.3 Hz, 2H), 6.96 (d, *J* = 8.3 Hz, 2H), 5.48 (s, 1H), 5.14 (s, 1H), 3.89 (s, 3H), 2.16 (d, *J* = 19.4 Hz, 3H); ^13^C NMR (100 MHz, DMSO-*d*_6_) *δ* (ppm): 152.12, 151.12, 145.65, 145.18, 142.77, 132.22, 131.22, 130.56, 125.65, 120.31, 115.32, 114.36, 55.01, 48.39, 20.35; EI-MS (*m*/*z*): 336, found 336 [M]^+^.

*Ethyl 1-oxo-3-(4-tolyl)-3*,*4-dihydrobenzo[4,5]imidazo[1,2-d][1,2,4]triazine-2(1H)-carboxylate* (**4o**): white powder; yield 35.3%; m.p. 178.1–180.0 °C; IR (KBr, cm^−1^) *v*: 1783, 1613, 1565, 1508; ^1^H NMR (400 MHz, DMSO-*d*_6_) *δ* (ppm): 7.95 (dd, *J* = 6.3, 2.5 Hz, 1H), 7.67 (dd, *J* = 6.3, 2.5 Hz, 1H), 7.40–7.32 (m, 2H), 7.08 (d, *J* = 8.5 Hz, 2H), 6.96 (d, *J* = 8.7 Hz, 2H), 5.48 (s, 1H), 5.14 (s, 1H), 4.36 (q, *J* = 7.1 Hz, 2H), 2.13 (s, 3H), 1.30 (t, *J* = 7.1 Hz, 3H); ^13^C NMR (100 MHz, DMSO-*d*_6_) *δ* (ppm): 151.57, 151.15, 145.68, 145.23, 142.77, 132.18, 131.22, 130.56, 125.62, 120.30, 115.28, 114.38, 64.22, 48.37, 20.36, 14.58; EI-MS (*m*/*z*): 350, found 350 [M]^+^.

*Propyl 1-oxo-3-(4-tolyl)-3*,*4-dihydrobenzo[4,5]imidazo[1,2-d][1,2,4]triazine-2(1H)-carboxylate* (**4p**): white powder; yield 34.2%; m.p. 145.0–146.8 °C; IR (KBr, cm^−1^) *v*: 1790, 1617, 1569, 1514; ^1^H NMR (400 MHz, DMSO-*d*_6_) *δ* (ppm): 8.01–7.90 (m, 1H), 7.75–7.62 (m, 1H), 7.44–7.29 (m, 2H), 7.08 (d, *J* = 8.4 Hz, 2H), 6.96 (d, *J* = 8.7 Hz, 2H), 5.49 (s, 1H), 5.12 (s, 1H), 4.28 (t, *J* = 6.5 Hz, 2H), 2.13 (s, 3H), 1.77–1.60 (m, 2H), 0.92 (t, *J* = 7.4 Hz, 3H); ^13^C NMR (100 MHz, DMSO-*d*_6_) *δ* (ppm): 151.65, 151.17, 145.70, 145.25, 142.78, 132.17, 131.23, 130.55, 125.61, 120.30, 115.28, 114.40, 69.45, 48.40, 22.00, 20.36, 10.59; EI-MS (*m*/*z*): 364, found 364 [M]^+^.

*Isopropyl 1-oxo-3-(4-tolyl)-3,4-dihydrobenzo[4,5]imidazo[1,2-d][1,2,4]triazine-2(1H)-carboxylate* (**4q**): yellow powder; yield 28.6%; m.p. 165.7–167.6 °C; IR (KBr, cm^−1^) *v*: 1784, 1713, 1613, 1569; ^1^H NMR (400 MHz, DMSO-*d*_6_) *δ* (ppm): 7.96 (d, 1H), 7.67 (d, *J* = 6.1, 2.5 Hz, 1H), 7.46–7.30 (m, 2H), 7.08 (d, *J* = 8.4 Hz, 2H), 6.95 (d, *J* = 8.5 Hz, 2H), 5.47 (s, 1H), 5.21–4.98 (m, 2H), 2.13 (s, 3H), 1.32 (d, *J* = 5.2 Hz, 6H); ^13^C NMR (100 MHz, DMSO-*d*_6_) *δ* (ppm): 151.18, 151.10, 145.72, 145.29, 142.80, 132.13, 131.25, 130.55, 125.58, 120.29, 115.25, 114.40, 72.52, 48.38, 22.01, 20.36; EI-MS (*m*/*z*): 364, found 364 [M]^+^.

*Isobutyl 1-oxo-3-(4-tolyl)-3,4-dihydrobenzo[4,5]imidazo[1,2-d][1,2,4]triazine-2(1H)-carboxylate* (**4r**): white powder; yield 35.3%; m.p. 115.7–116.8 °C; IR 1785, 1751, 1617, 1572; ^1^H NMR (400 MHz, DMSO-*d*_6_) *δ* (ppm): 7.96 (dd, *J* = 6.3, 2.6 Hz, 1H), 7.67 (dd, *J* = 6.1, 2.6 Hz, 1H), 7.37 (pd, *J* = 7.4, 3.7 Hz, 2H), 7.08 (d, *J* = 8.5 Hz, 2H), 6.95 (d, *J* = 8.7 Hz, 2H), 5.49 (s, 1H), 5.13 (s, 1H), 4.11 (d, *J* = 6.5 Hz, 2H), 2.14 (s, 3H), 2.03–1.89 (m, 1H), 0.93 (d, *J* = 6.7 Hz, 6H); ^13^C NMR (100 MHz, DMSO-*d*_6_) *δ* (ppm): 151.64, 151.17, 145.72, 145.26, 142.79, 132.18, 131.24, 130.55, 125.62, 120.29, 115.28, 114.42, 73.57, 48.46, 27.83, 20.37, 19.43, 19.11; EI-MS (*m*/*z*): 378, found 378 [M]^+^.

*Phenyl 1-oxo-3-(4-tolyl)-3*,*4-dihydrobenzo[4,5]imidazo[1,2-d][1,2,4]triazine-2(1H)-carboxylate* (**4s**): white powder; yield 37.3%; m.p. 184.2–185.6 °C; IR (KBr, cm^−1^) *v*: 1749, 1569, 1510, 1453; ^1^H NMR (400 MHz, DMSO-*d*_6_) *δ* (ppm): 7.98 (dd, *J* = 6.5, 2.5 Hz, 1H), 7.71 (dd, *J* = 6.2, 2.2 Hz, 1H), 7.54–7.46 (m, 2H), 7.40 (ddd, *J* = 5.9, 5.3, 3.7 Hz, 2H), 7.35 (t, *J* = 7.0 Hz, 3H), 7.16–7.07 (m, 4H), 5.58 (s, 1H), 5.32 (s, 1H), 2.16 (s, 3H); ^13^C NMR (100 MHz, DMSO-*d*_6_) *δ* (ppm): 151.12, 150.73, 145.49, 145.04, 142.79, 132.43, 131.79, 130.70, 130.26, 127.01, 125.82, 122.06, 120.42, 115.40, 114.44, 48.29, 20.40; EI-MS (*m*/*z*): 398, found 398 [M]^+^.

*Ethyl 1-oxo-3-(4-fluorophenyl)-3*,*4-dihydrobenzo[4,5]imidazo[1,2-d][1,2,4]triazine-2(1H)-carboxylate* (**4t**): yellow powder; yield 31.1%; m.p. 159.9–161.5 °C; IR (KBr, cm^−1^) *v*: 1784, 1709, 1615, 1569; ^1^H NMR (400 MHz, DMSO-*d*_6_) *δ* (ppm): 7.96 (d, *J* = 7.0 Hz, 1H), 7.67 (t, *J* = 13.3 Hz, 1H), 7.49–7.29 (m, 2H), 7.23–6.99 (m, 4H), 5.52 (s, 1H), 5.16 (s, 1H), 4.37 (q, *J* = 6.9 Hz, 2H), 1.40–1.24 (m, 3H); ^13^C NMR (100 MHz, DMSO-*d*_6_) *δ* (ppm): 151.49, 150.99, 145.52, 143.96, 142.78, 131.28, 125.70, 120.36, 117.13, 116.90, 116.68, 114.44, 64.35, 48.54, 14.57; EI-MS (*m*/*z*): 354, found 354 [M]^+^.

*Ethyl 1-oxo-3-(4-bromophenyl)-3,4-dihydrobenzo[4,5]imidazo[1,2-d][1,2,4]triazine-2(1H)-carboxylate* (**4u**): yellow powder; yield 34.2%; m.p. 151.5–153.4 °C; IR (KBr, cm^−1^) *v*: 1590, 1487, 1451, 1437; ^1^H NMR (400 MHz, DMSO-*d*_6_) *δ* (ppm): 7.96 (d, *J* = 7.2 Hz, 1H), 7.69 (d, *J* = 7.1 Hz, 1H), 7.47 (d, *J* = 8.0 Hz, 2H), 7.43–7.33 (m, 2H), 7.07 (d, *J* = 8.0 Hz, 2H), 5.57 (s, 1H), 5.18 (s, 1H), 4.38 (dd, *J* = 13.8, 6.8 Hz, 2H), 1.31 (t, *J* = 6.9 Hz, 3H); ^13^C NMR (100 MHz, DMSO-*d*_6_) *δ* (ppm): 151.35, 150.86, 146.92, 145.34, 142.76, 132.86, 131.22, 125.72, 120.36, 117.50, 115.03, 114.43, 64.42, 48.15, 14.56; EI-MS (*m*/*z*): 416, found 416 [M]^+^.

*Methyl 1-oxo-3-(3-chlorophenyl)-3,4-dihydrobenzo[4,5]imidazo[1,2-d][1,2,4]triazine-2(1H)-carboxylate* (**4v**): yellow powder; yield 28.9%; m.p. 196.2–198.5 °C; IR (KBr, cm^−1^) *v*: 1786, 1706, 1616, 1571; ^1^H NMR (400 MHz, DMSO-*d*_6_) *δ* (ppm): 7.96 (d, *J* = 6.8 Hz, 1H), 7.70 (d, *J* = 7.0 Hz, 1H), 7.40 (td, *J* = 12.4, 7.9 Hz, 2H), 7.35–7.27 (m, 1H), 7.23 (d, *J* = 1.8 Hz, 1H), 7.03 (t, *J* = 9.4 Hz, 2H), 5.63 (s, 1H), 5.14 (s, 1H), 3.90 (s, 3H); ^13^C NMR (100 MHz, DMSO-*d*_6_) *δ* (ppm): 151.87, 150.85, 149.01, 145.27, 142.75, 134.90, 131.90, 131.20, 125.80, 123.00, 120.42, 115.17, 114.47, 113.83, 55.21, 48.07; EI-MS (*m*/*z*): 358, found 358 [M]^+^.

*Methyl 1-oxo-3-phenyl-4-methyl-3,4-dihydrobenzo[4,5]imidazo[1,2-d][1,2,4]triazine-2(1H)-carboxylate* (**4w**): yellow powder; yield 26.1%; m.p. 120.0–121.9 °C; IR (KBr, cm^−1^) *v*: 1787, 1706, 1616, 1571; ^1^H NMR (400 MHz, DMSO-*d*_6_) *δ* (ppm): 8.00 (d, *J* = 7.8 Hz, 1H), 7.68 (d, *J* = 7.0 Hz, 1H), 7.44–7.35 (m, 2H), 7.30 (t, *J* = 7.9 Hz, 2H), 7.13 (d, *J* = 8.1 Hz, 2H), 6.97 (t, *J* = 7.2 Hz, 1H), 5.84 (dd, *J* = 13.4, 6.5 Hz, 1H), 3.93 (s, 3H), 1.70 (d, *J* = 6.8 Hz, 3H); ^13^C NMR (100 MHz, DMSO-*d*_6_) *δ* (ppm): 153.56, 152.70, 147.58, 145.33, 142.70, 131.06, 130.23, 125.82, 123.46, 120.42, 115.69, 114.63, 55.31, 53.90, 17.83; EI-MS (*m*/*z*): 336, found 336 [M]^+^.

### 3.3. Single Crystal X-ray Diffraction Analysis

The single crystal of compound **4n** was recrystallized from tetrahydrofuran to obtain a suitable single crystal. The X-ray single crystal diffraction data were collected on a Bruker Smart APEX Ⅱ CCD Single-crystal diffractometer (Bruker, German) with graphite monochromatized Mo K*α* radiation (*λ* = 0.71073 Å) using a *φ*-*ω* scan mode at 296(2)K. The obtained crystal structure solved directly and was refined by full-matrix least-squares method via SHELXTL [26]. The crystallographic data for the compound **4n** have been deposited in CCDC-1543178. These data can be obtained free of charge via http://www.ccdc.cam.ac.uk (or from the CCDC, 12 Union Road, Cambridge CB2 1EZ, UK; Fax: +44-1223-336033; E-mail: deposit@ccdc.cam.ac.uk).

### 3.4. Antifungal Bioassay

The antifungal activity of all the title compounds was evaluated in vitro with the mycelium growth rate method [25]. The tested strains *B. cinerea*, *R. solani,* and *C. capsici*, which were, respectively, isolated from infected strawberries, rice, and peppers in disease outbreak regions in Jiangsu Province, were provided by the Laboratory of Plant Disease Control at Nanjing Agricultural University. Every sample (4.5 mg) was dissolved in 0.5 mL methanol and evenly mixed with 89.5 mL PSA (potato sucrose agar) medium. An equal volume of methanol in 89.5 mL medium was used as the blank control. Meanwhile, the commercial agricultural fungicide carbendazim was tested as the positive control at the same concentration. Each 15 mL medium was poured into a 9 cm petri plate with 3 replicates. The fungi were inoculated to the center of the medium and cultured at 25 ± 1 °C for 3–5 days in a dark environment. After a certain incubation period (2.5 d for *B. cinerea*, 1.5 d for *R. solani,* and 4 d for *C. capsici*), the diameters of the mycelium colonies were measured, and the inhibitory percentages were calculated.

## 4. Conclusions

In continuation of our research searching for novel agricultural fungicides, a series of novel benzo[4,5]imidazo[1,2-*d*][1,2,4]triazine derivatives were synthesized by a practical and convenient reaction route. These compounds were well supported by spectroscopic data and single crystal X-ray diffraction analysis. The bioassays indicated that most of the title compounds exhibited obvious antifungal activities in vitro at 50 μg/mL. Further studies on structure optimization of benzo[4,5]imidazo[1,2-*d*][1,2,4]triazines as a leading structure of novel agricultural fungicides are well underway.

## Figures and Tables

**Table 1:** Crystal data of title compound **4n**.

Crystal Data	
C_18_H_16_N_4_O_3_	*α* = 118.323 (7)°
FW = 336.35	*β* = 91.750 (8)°
Triclinic, *P*$\bar{1}$	*γ* = 104.198 (7)°
*a* = 9.033 (6) Å	*V* = 800.2 (9) Å^3^
*b* = 10.136 (7) Å	*Z* = 2
*c* = 10.396 (7) Å	*μ* = 0.098 mm^−1^
*F* (000) = 352	Cryatal size (mm^3^): 0.38 × 0.40 × 0.45
Data Collection	
*T*_min_ = 0.9559, *T*_max_ = 0.9642	2715 observed reflections with *I* > 2*σ*(*I*)
6357 measured reflections	*R*_int_ = 0.031
3195 independent reflections	*θ*_min_ = 2.3°, *θ*_max_ = 26.4°
Refinement	
229 parameters	*R* indices (all data) *R*_1_ = 0.0475, w*R*_2_ = 0.1284
Goodness-of-fit: 1.054	*R* indices *[I* > 2*σ*(*I*)] *R*_1_ = 0.0408, w*R*_2_ = 0.1212
Δ*ρ*_max_ = 0.22 eÅ^−3^, Δ*ρ*_min_ = −0.23 eÅ^−3^	*w* [*P* = (*F_o_*^2^+ 2*F_c_*^2^)/3] = 1/[*σ*^2^(*F_o_*^2^) + (0.0680*P*)^2^ + 0.1590*P*]

**Table 2:** Antifungal activity of compounds **4** against three test fungi at 50 μg/mL.

Compd.	R^1^	R^2^	R^3^	Inhibition (%)		
				*B. cinerea*	*R. solani*	*C. capsici*
**4a**	H	H	Me	62.1 ± 0.2	32.0 ± 0.6	52.1 ± 0.2
**4b**	H	H	Et	41.1 ± 0.1	63.5 ± 0.7	29.8 ± 0.3
**4c**	H	H	*n*-Pr	11.4 ± 0.1	45.7 ± 1.0	27.4 ± 0.3
**4d**	H	H	*i*-Pr	29.7 ± 0.1	33.2 ± 0.4	38.0 ± 0.2
**4e**	H	H	*i*-Bu	28.3 ± 0.1	48.1 ± 0.3	21.2 ± 0.2
**4f**	H	H	Ph	26.2 ± 0.3	57.3 ± 0.2	27.7 ± 0.1
**4g**	H	4-Cl	Me	70.5 ± 0.1	58.0 ± 0.2	60.3 ± 0.1
**4h**	H	4-Cl	Et	68.6 ± 0.1	53.7 ± 0.2	49.4 ± 0.5
**4i**	H	4-Cl	*n*-Pr	68.4 ± 0.4	50.6 ± 0.3	43.4 ± 0.5
**4j**	H	4-Cl	*i*-Pr	51.5 ± 0.1	40.2 ± 0.2	43.4 ± 0.2
**4k**	H	4-Cl	*i*-Bu	50.8 ± 0.2	31.3 ± 0.6	45.7 ± 0.1
**4l**	H	4-Cl	Ph	50.5 ± 0.3	47.7 ± 0.3	57.8 ± 0.2
**4m**	H	4-Cl	Bn	50.8 ± 0.1	21.6 ± 0.4	39.4 ± 0.4
**4n**	H	4-Me	Me	69.2 ± 0.3	53.9 ± 0.5	58.9 ± 0.4
**4o**	H	4-Me	Et	76.7 ± 0.1	57.8 ± 0.3	40.5 ± 0.2
**4p**	H	4-Me	*n*-Pr	42.3 ± 0.2	52.8 ± 0.2	18.7 ± 0.2
**4q**	H	4-Me	*i*-Pr	52.1 ± 0.2	27.9 ± 0.3	31.8 ± 0.4
**4r**	H	4-Me	*i*-Bu	13.3 ± 0.3	34.4 ± 0.3	14.7 ± 0.3
**4s**	H	4-Me	Ph	19.3 ± 0.1	61.4 ± 0.1	27.9 ± 0.2
**4t**	H	4-F	Et	46.8 ± 0.1	50.2 ± 0.1	66.8 ± 0.0
**4u**	H	4-Br	Et	64.4 ± 0.1	60.6 ± 0.2	51.7 ± 0.2
**4v**	H	3-Cl	Me	62.1 ± 0.1	61.1 ± 0.2	60.1 ± 0.2
**4w**	Me	H	Me	45.9 ± 0.1	28.1 ± 0.2	26.6 ± 0.1
**Carbendazim**	—	—	—	100 ± 0.0	100 ± 0.0	87.5 ± 0.0

## Supplementary Materials

The supplementary materials containing FT-IR, ^1^H NMR, ^13^C NMR, EI-MS spectra of the title compounds and the crystal structure data of compound **4n** can be accessed online.
