# Supplementary material for: Synthesis, Characterization, and Antifungal Activity of Novel Benzo[4,5]imidazo[1,2-d][1,2,4]triazine Derivatives
Source: Molecules. 2018 Mar 23;23(4):746. doi: 10.3390/molecules23040746 (PMC6017302; doi:10.3390/molecules23040746)
Supplement: Supplementary file 1 [file molecules-23-00746-s001.pdf]

Supporting Information For:

Synthesis, Characterization and Antifungal Activity of Novel Benzo[4,5]imidazo[1,2-*d*][1,2,4]triazine Derivatives

Ling-Xia Li[1,2,†], Jian Jiao[1,2,†], Xiao-Bin Wang[1], Min Chen[1,2], Xin-Can Fu[1], Wei-Jie Si[1,3] and Chun-Long Yang[1,2,3,*]

1 College of Sciences, Nanjing Agricultural University, Nanjing 210095, China; 2014111007@njau.edu.cn (L.-X. Li); 2015111008@njau.edu.cn (J. Jiao)

2 Jiangsu Key Laboratory of Pesticide Science, Nanjing Agricultural University, Nanjing 210095, China;

3 Key Laboratory of Monitoring and Management of Crop Diseases and Pest Insects, Ministry of Agriculture, Nanjing Agricultural University, Nanjing 210095, China;

* Correspondence: ycl@njau.edu.cn (C.-L Yang); Tel.: +86-25-8439-5207; Fax: +86-25-8439-5207.

† These authors contributed equally to this paper.

Contents

1. ^{1}H NMR spectra of compound **4a** 4
2. ^{13}C NMR spectra of compound **4a** 4
3. EI-MS spectra of compound **4a** 5
4. FT-IR spectra of compound **4a** 5
5. ^{1}H NMR spectra of compound **4b** 6
6. ^{13}C NMR spectra of compound **4b** 6
7. EI-MS spectra of compound **4b** 7
8. FT-IR spectra of compound **4b** 7
9. ^{1}H NMR spectra of compound **4c** 8
10. ^{13}C NMR spectra of compound **4c** 8
11. EI-MS spectra of compound **4c** 9
12. FT-IR spectra of compound **4c** 9
13. ^{1}H NMR spectra of compound **4d** 9
14. ^{13}C NMR spectra of compound **4d** 10
15. EI-MS spectra of compound **4d** 10
16. FT-IR spectra of compound **4d** 11
17. ^{1}H NMR spectra of compound **4e** 11
18. ^{13}C NMR spectra of compound **4e** 12
19. EI-MS spectra of compound **4e** 12
20. FT-IR spectra of compound **4e** 13
21. ^{1}H NMR spectra of compound **4f** 13
22. ^{13}C NMR spectra of compound **4f** 14
23. EI-MS spectra of compound **4f** 14

24. FT-IR spectra of compound **4f** 15
25. ^{1}H NMR spectra of compound **4g** 15
26. ^{13}C NMR spectra of compound **4g** 16
27. EI-MS spectra of compound **4g** 16
28. FT-IR spectra of compound **4g** 17
29. ^{1}H NMR spectra of compound **4h** 17
30. ^{13}C NMR spectra of compound **4h** 18
31. EI-MS spectra of compound **4h** 18
32. FT-IR spectra of compound **4h** 19
33. ^{1}H NMR spectra of compound **4i** 19
34. ^{13}C NMR spectra of compound **4i** 20
35. EI-MS spectra of compound **4i** 20
36. FT-IR spectra of compound **4i** 21
37. ^{1}H NMR spectra of compound **4j** 21
38. ^{13}C NMR spectra of compound **4j** 21
39. EI-MS spectra of compound **4j** 22
40. FT-IR spectra of compound **4j** 23
41. ^{1}H NMR spectra of compound **4k** 23
42. ^{13}C NMR spectra of compound **4k** 24
43. EI-MS spectra of compound **4k** 24
44. FT-IR spectra of compound **4k** 25
45. ^{1}H NMR spectra of compound **4l** 25
46. ^{13}C NMR spectra of compound **4l** 26
47. EI-MS spectra of compound **4l** 26
48. FT-IR spectra of compound **4l** 27
49. ^{1}H NMR spectra of compound **4m** 27
50. ^{13}C NMR spectra of compound **4m** 28
51. EI-MS spectra of compound **4m** 28
52. FT-IR spectra of compound **4m** 29
53. ^{1}H NMR spectra of compound **4n** 29
54. ^{13}C NMR spectra of compound **4n** 30
55. EI-MS spectra of compound **4n** 30
56. FT-IR spectra of compound **4n** 31
57. ^{1}H NMR spectra of compound **4o** 31
58. ^{13}C NMR spectra of compound **4o** 32
59. EI-MS spectra of compound **4o** 32
60. FT-IR spectra of compound **4o** 33
61. ^{1}H NMR spectra of compound **4p** 33
62. ^{13}C NMR spectra of compound **4p** 34
63. EI-MS spectra of compound **4p** 34
64. FT-IR spectra of compound **4p** 35
65. ^{1}H NMR spectra of compound **4q** 35
66. ^{13}C NMR spectra of compound **4q** 36
67. EI-MS spectra of compound **4q** 36

68. FT-IR spectra of compound **4q** 37
69. ^{1}H NMR spectra of compound **4r** 37
70. ^{13}C NMR spectra of compound **4r** 38
71. EI-MS spectra of compound **4r** 38
72. FT-IR spectra of compound **4r** 38
73. ^{1}H NMR spectra of compound **4s** 39
74. ^{13}C NMR spectra of compound **4s** 39
75. EI-MS spectra of compound **4s** 40
76. FT-IR spectra of compound **4s** 41
77. ^{1}H NMR spectra of compound **4t** 41
78. ^{13}C NMR spectra of compound **4t** 42
79. EI-MS spectra of compound **4t** 42
80. FT-IR spectra of compound **4t** 43
81. ^{1}H NMR spectra of compound **4u** 43
82. ^{13}C NMR spectra of compound **4u** 44
83. EI-MS spectra of compound **4u** 44
84. FT-IR spectra of compound **4u** 45
85. ^{1}H NMR spectra of compound **4v** 45
86. ^{13}C NMR spectra of compound **4v** 45
87. EI-MS spectra of compound **4v** 46
88. FT-IR spectra of compound **4v** 47
89. ^{1}H NMR spectra of compound **4w** 47
90. ^{13}C NMR spectra of compound **4w** 48
91. EI-MS spectra of compound **4w** 48
92. FT-IR spectra of compound **4w** 49
93. Selected geometric parameters of compound **4n**. 49
94. Hydrogen bonding data for compound **4n** (Å, °). 50
95. Selected distances between hydrogen and carbon atoms for compound **4n** (Å). 50

^{1}H NMR spectra of compound **4a**

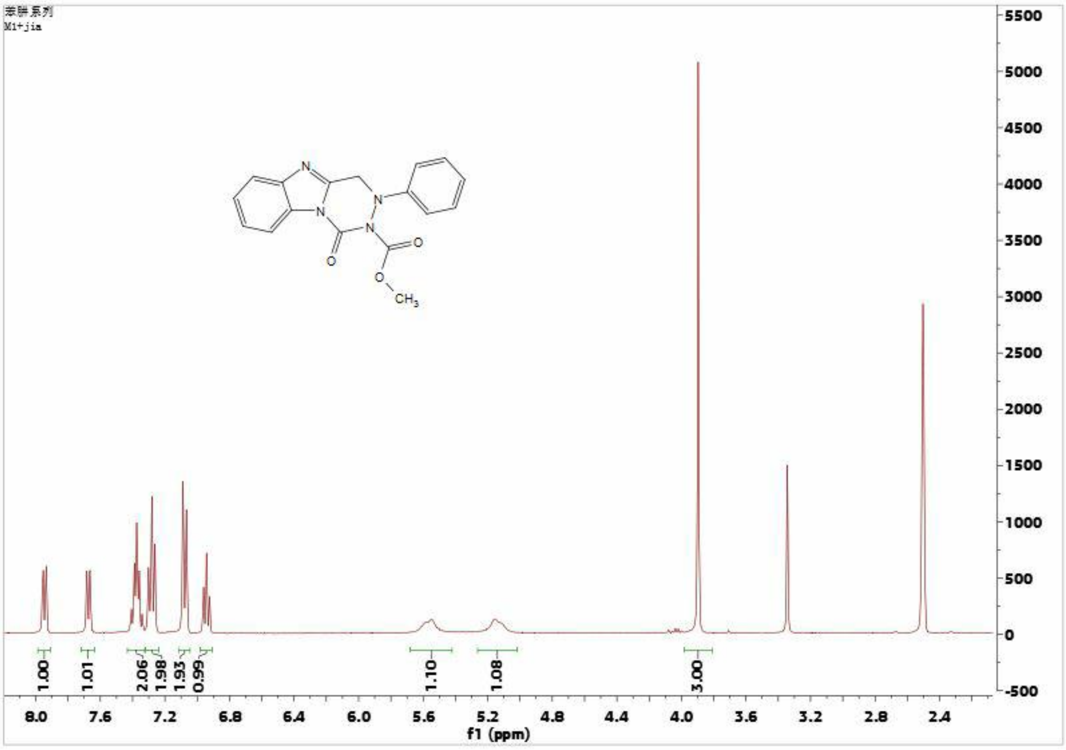

1. ^{13}C NMR spectra of compound **4a**

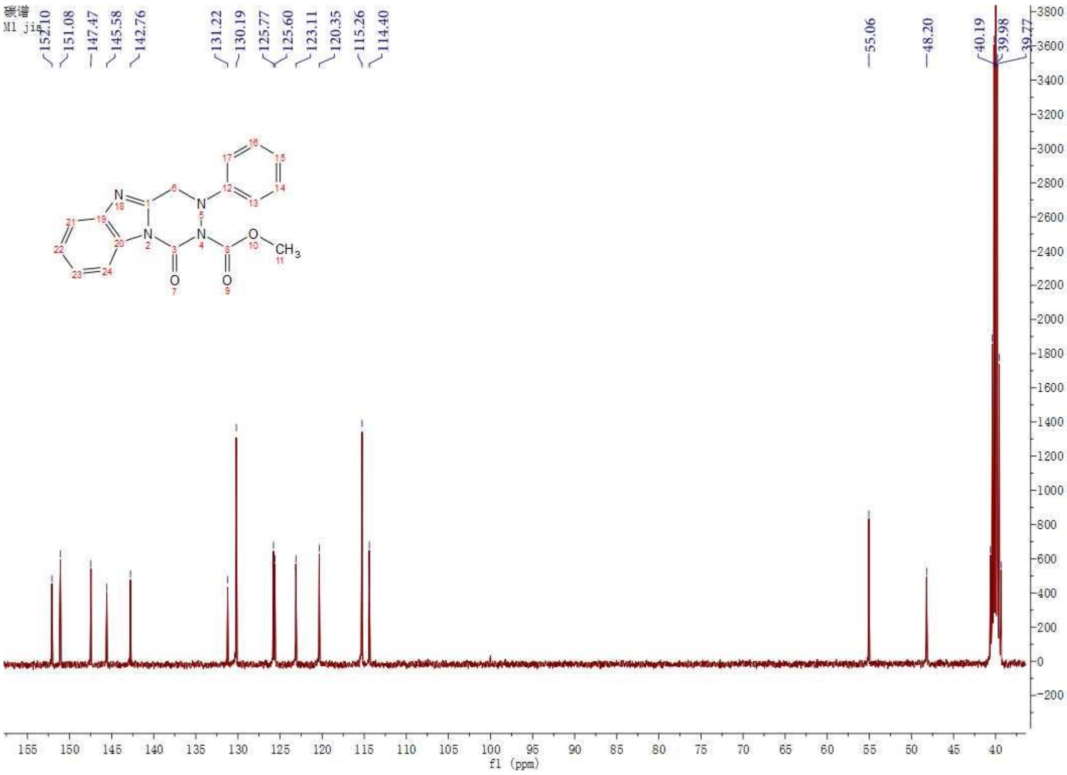

2. EI-MS spectra of compound **4a**

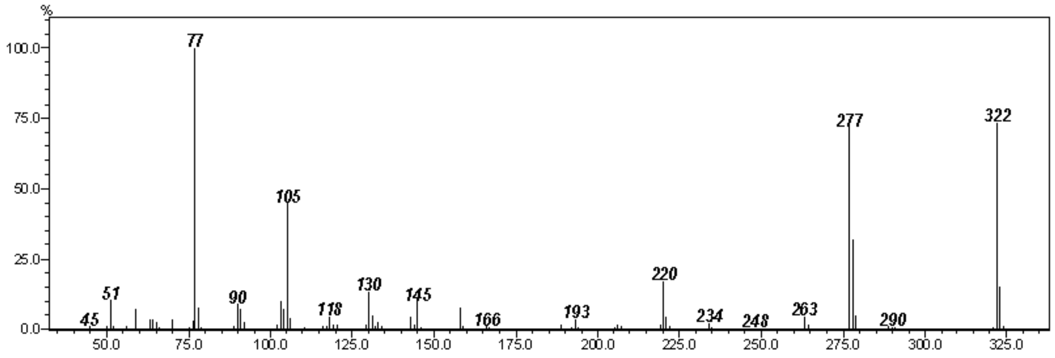

3. FT-IR spectra of compound **4a**

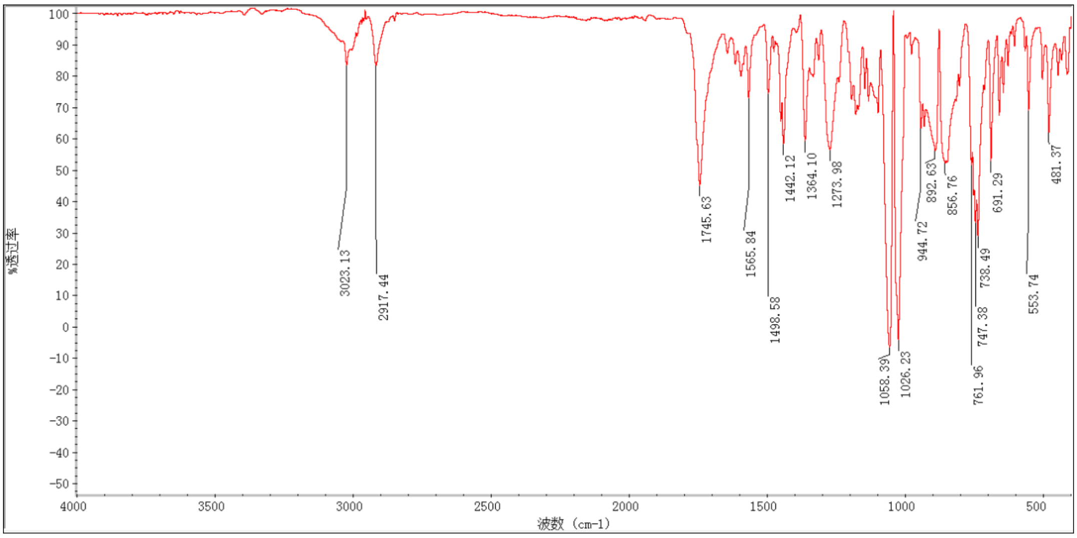

4. ^{1}H NMR spectra of compound **4b**

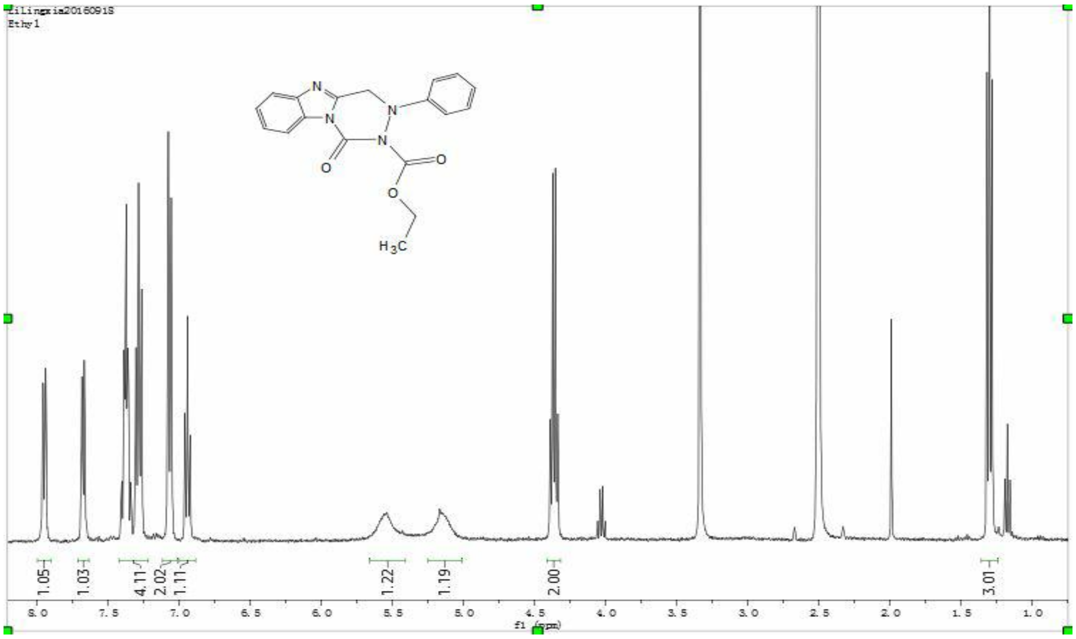

5. ^{13}C NMR spectra of compound **4b**

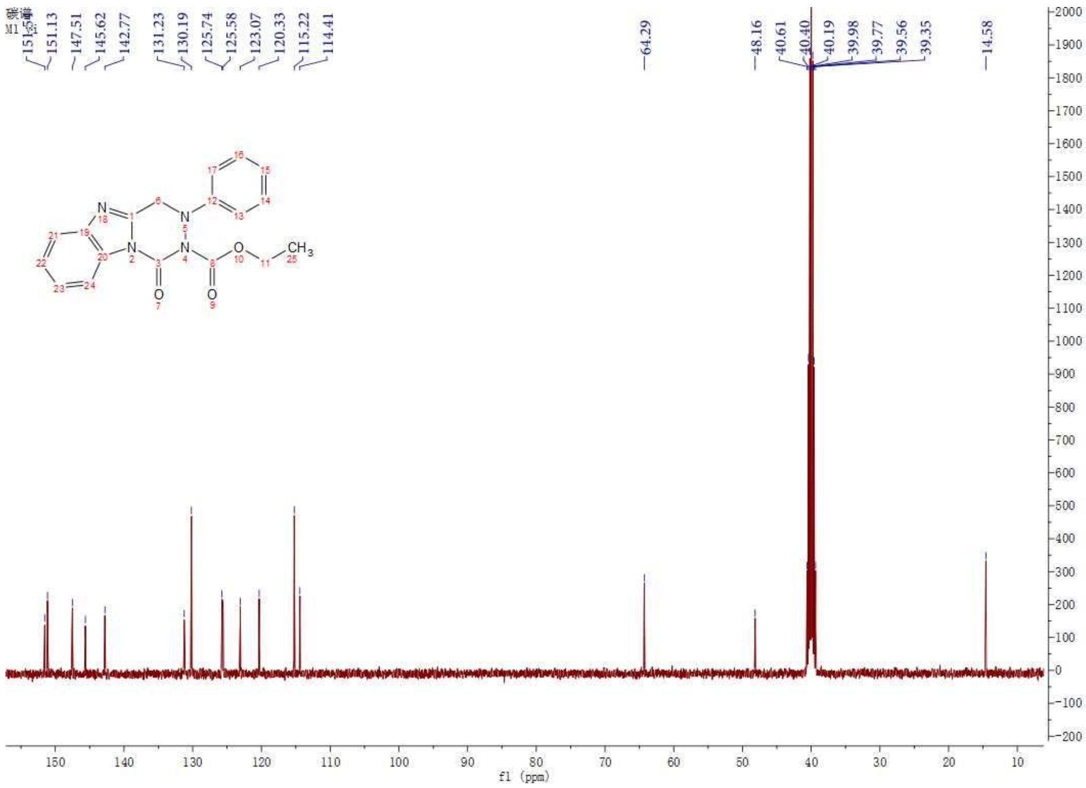

6. EI-MS spectra of compound **4b**

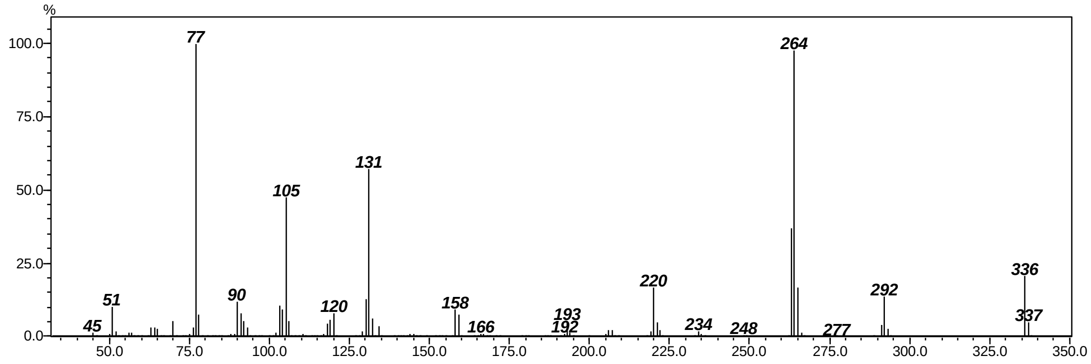

7. FT-IR spectra of compound **4b**

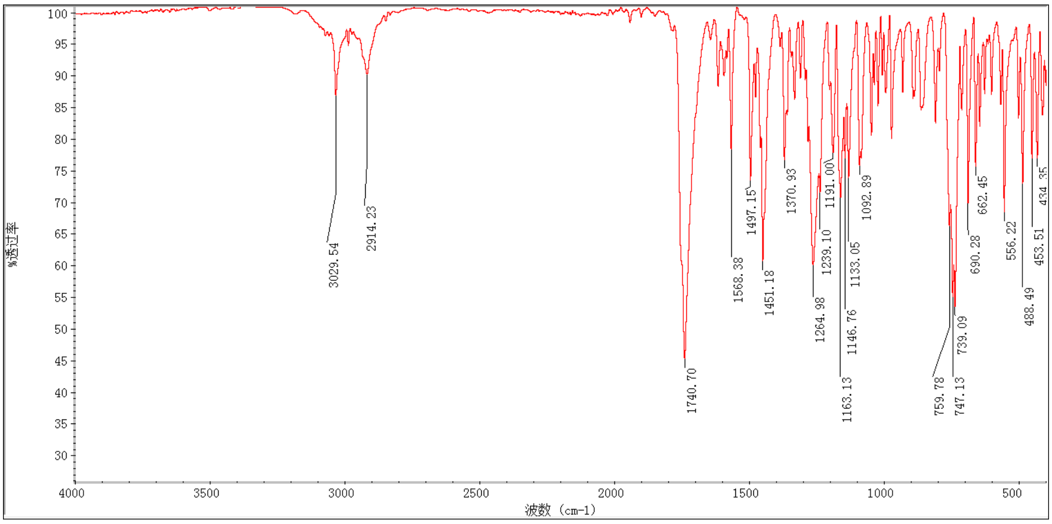

8. ^{1}H NMR spectra of compound **4c**

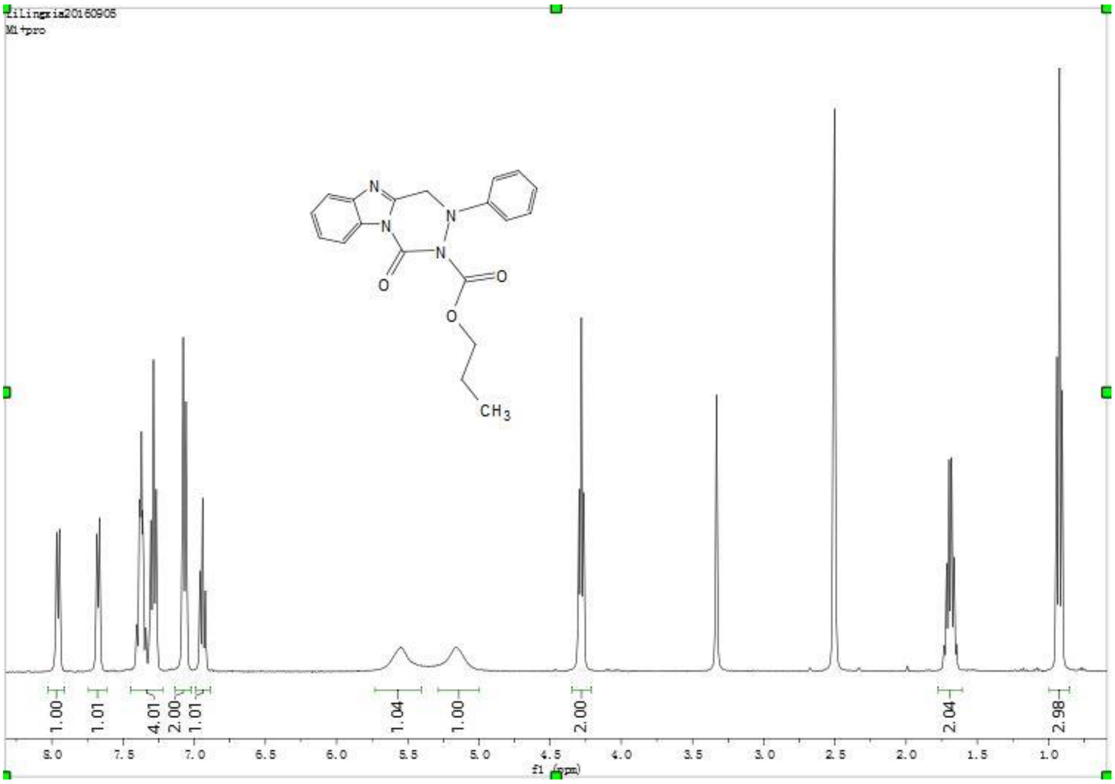

9. ^{13}C NMR spectra of compound **4c**

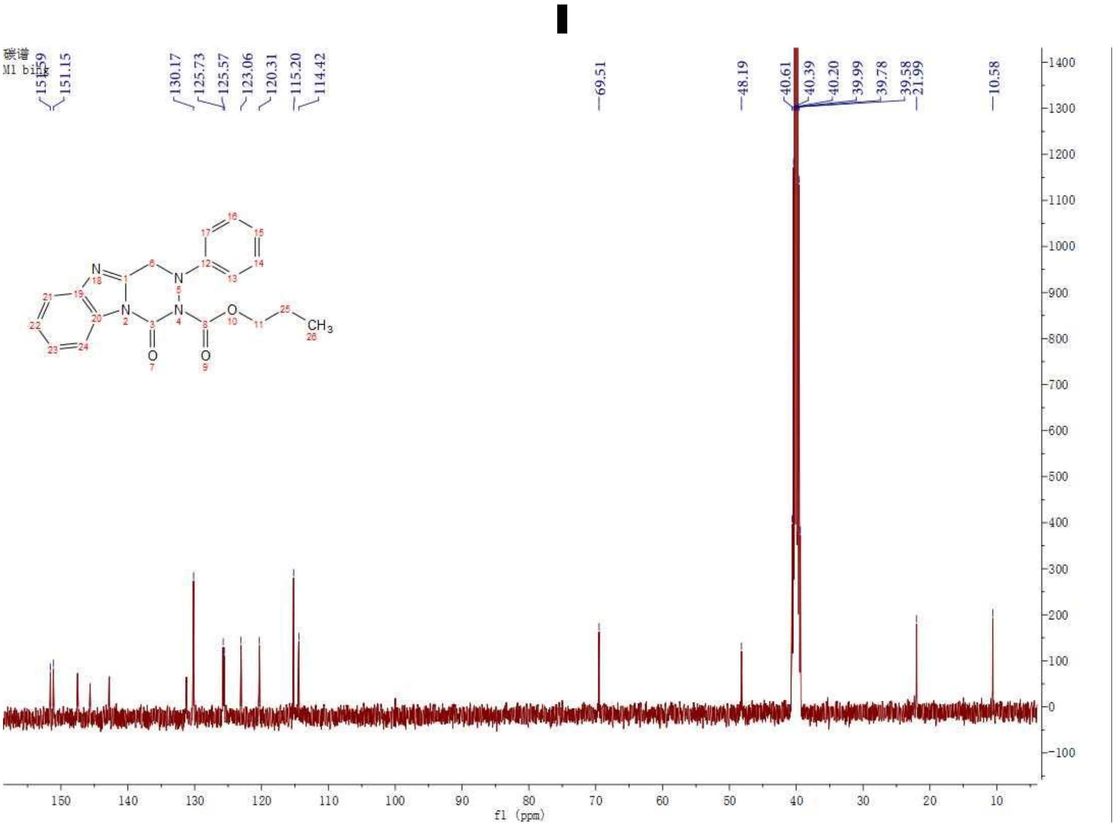

10. EI-MS spectra of compound **4c**

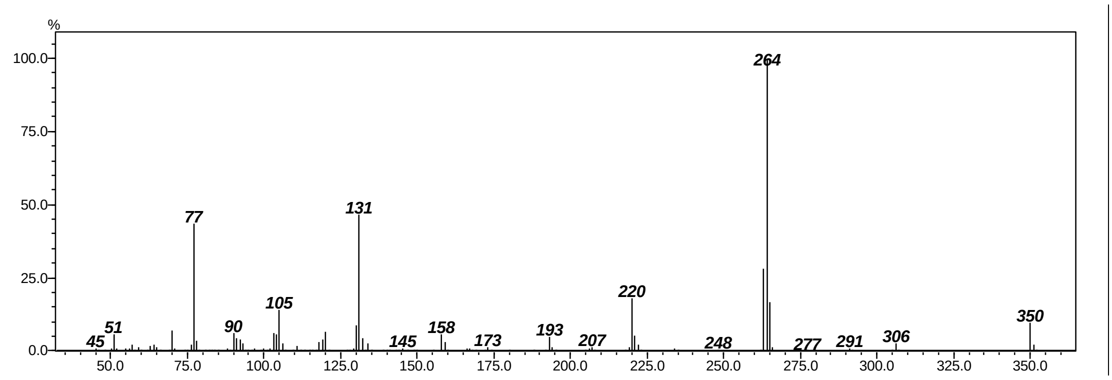

11. FT-IR spectra of compound **4c**

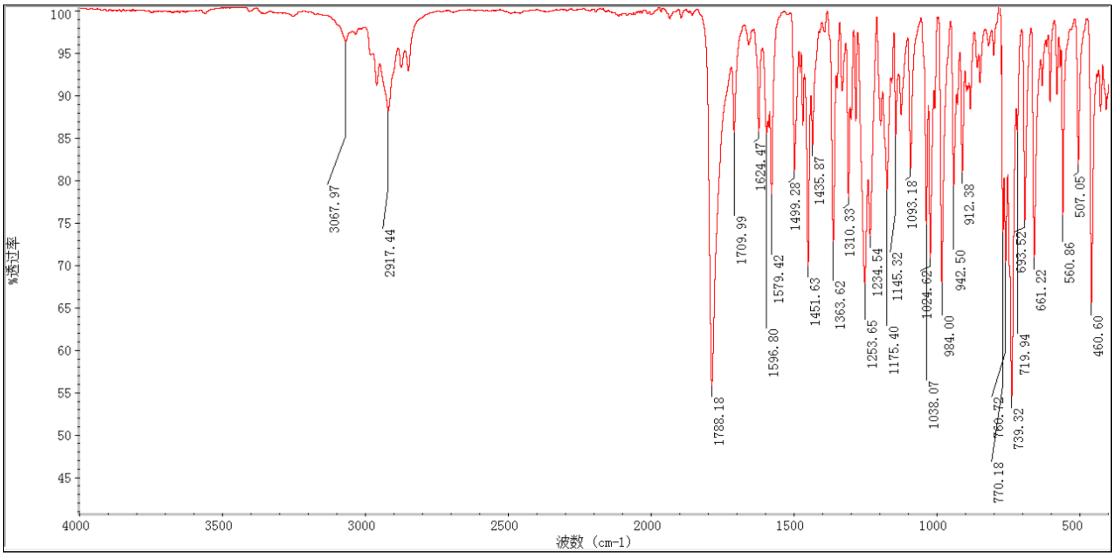

12. ^{1}H NMR spectra of compound **4d**

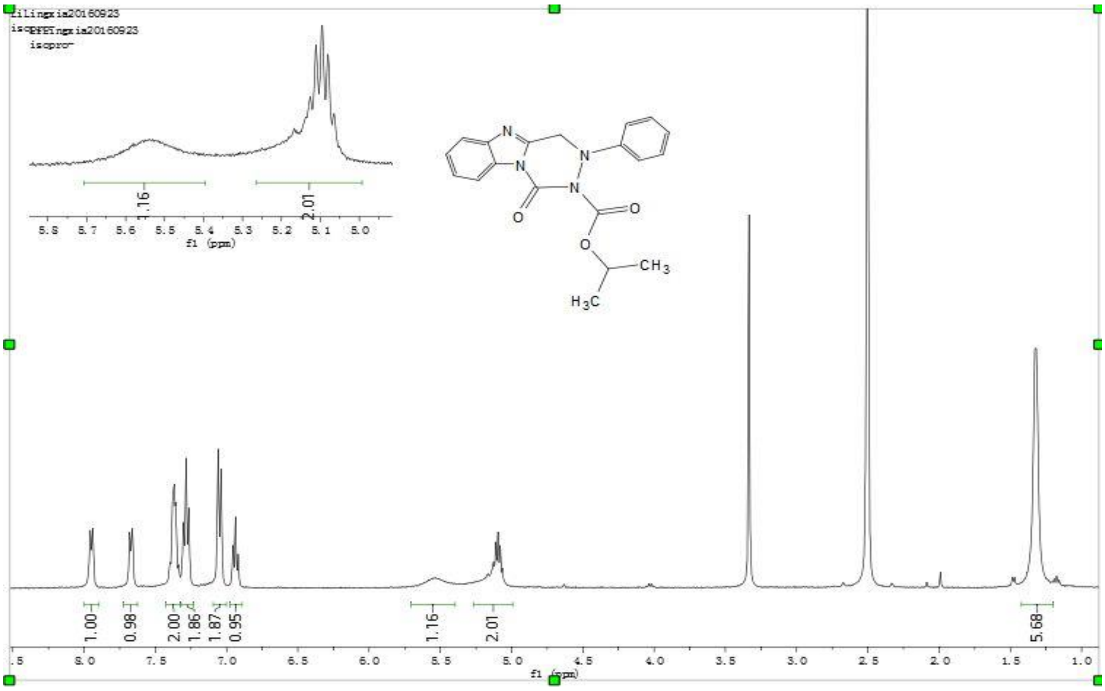

13. ^{13}C NMR spectra of compound **4d**

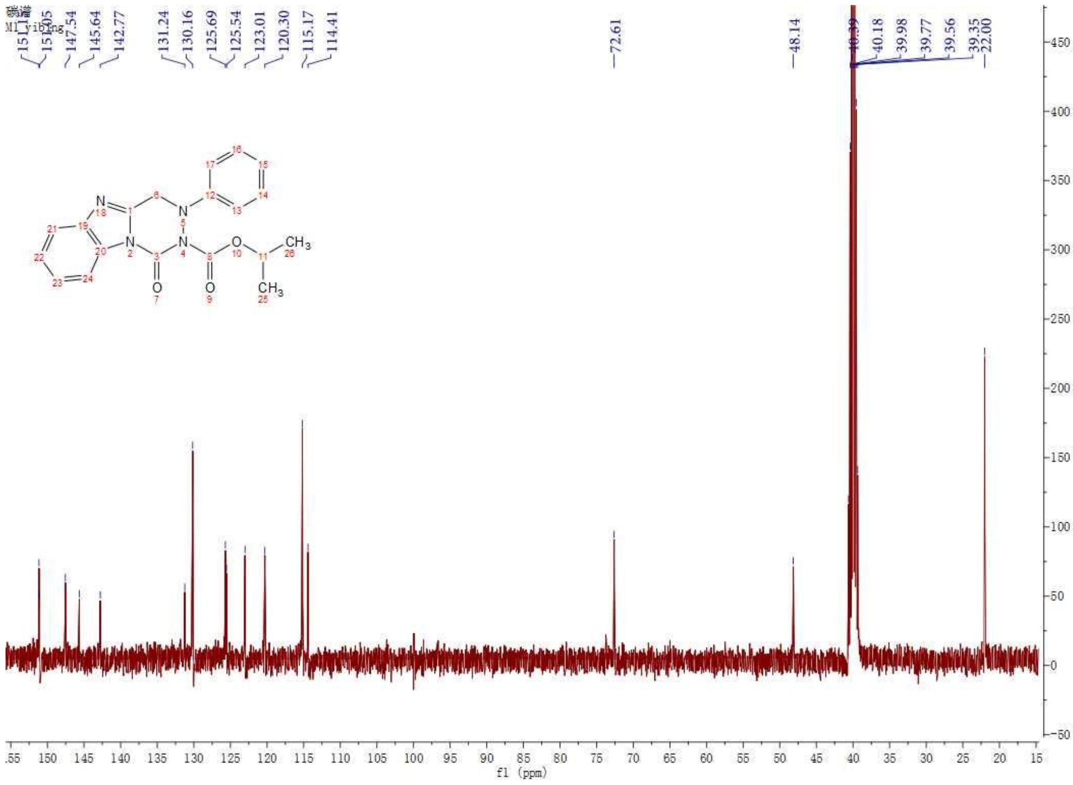

14. EI-MS spectra of compound **4d**

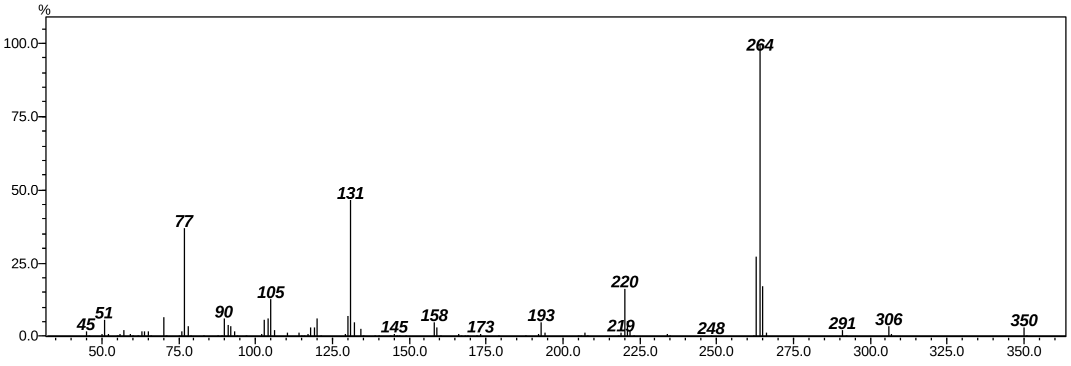

15. FT-IR spectra of compound **4d**

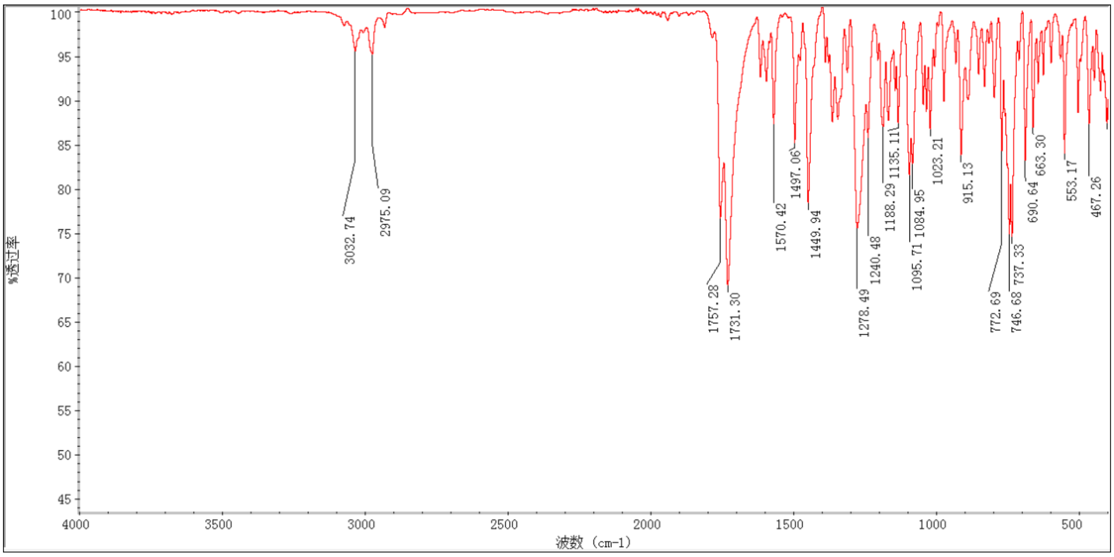

16. ^{1}H NMR spectra of compound **4e**

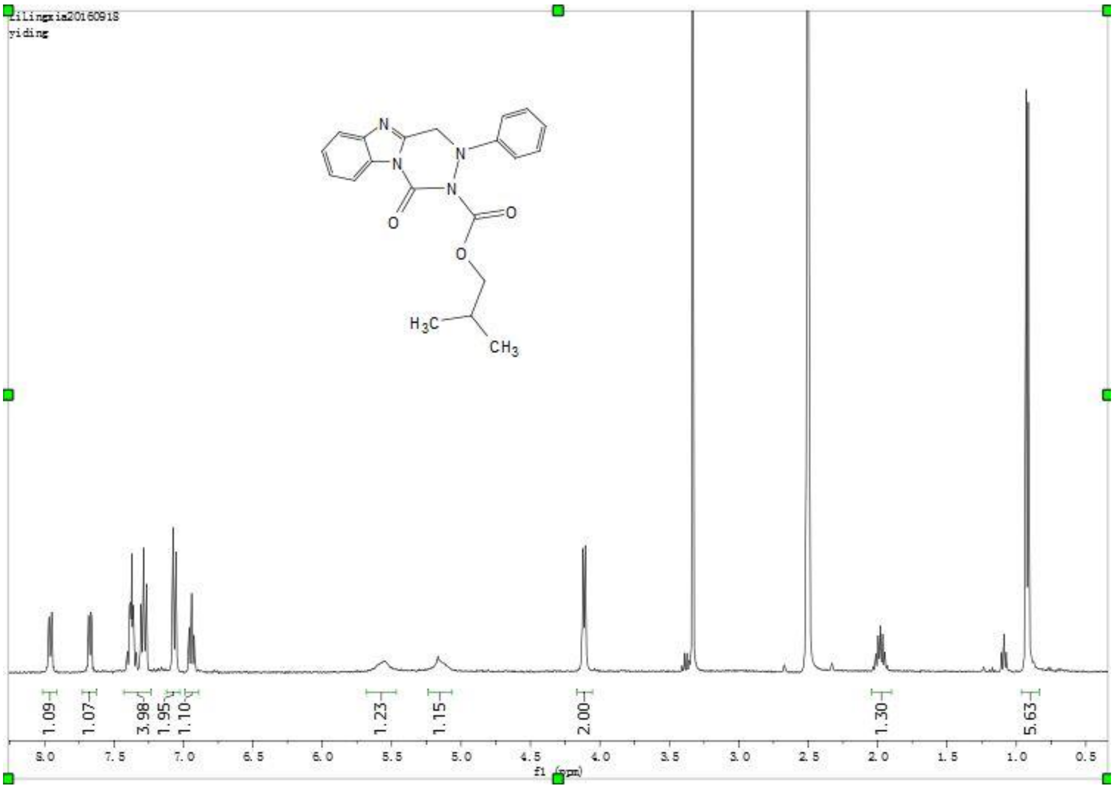

17. ^{13}C NMR spectra of compound **4e**

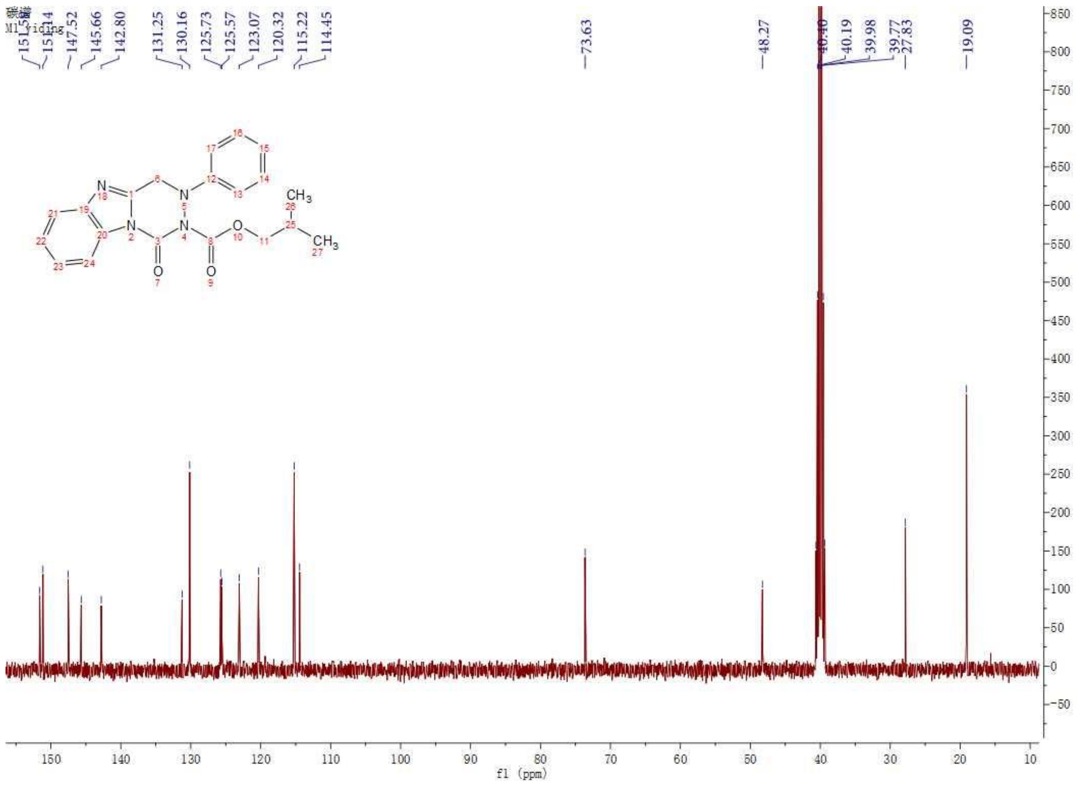

18. EI-MS spectra of compound **4e**

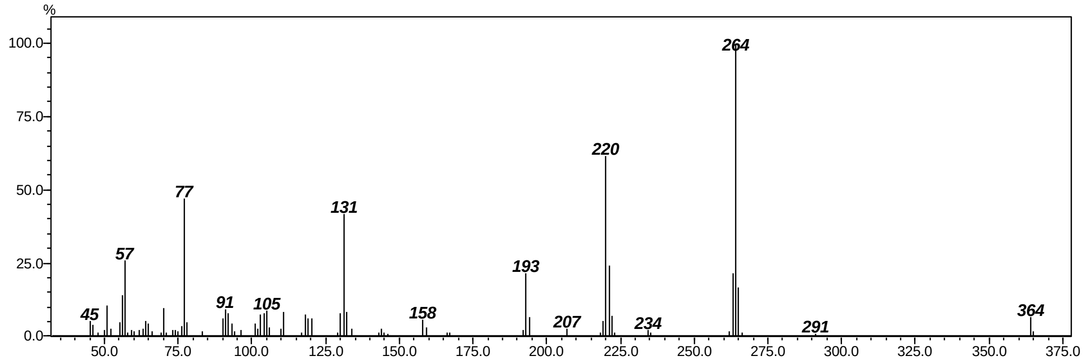

19. FT-IR spectra of compound **4e**

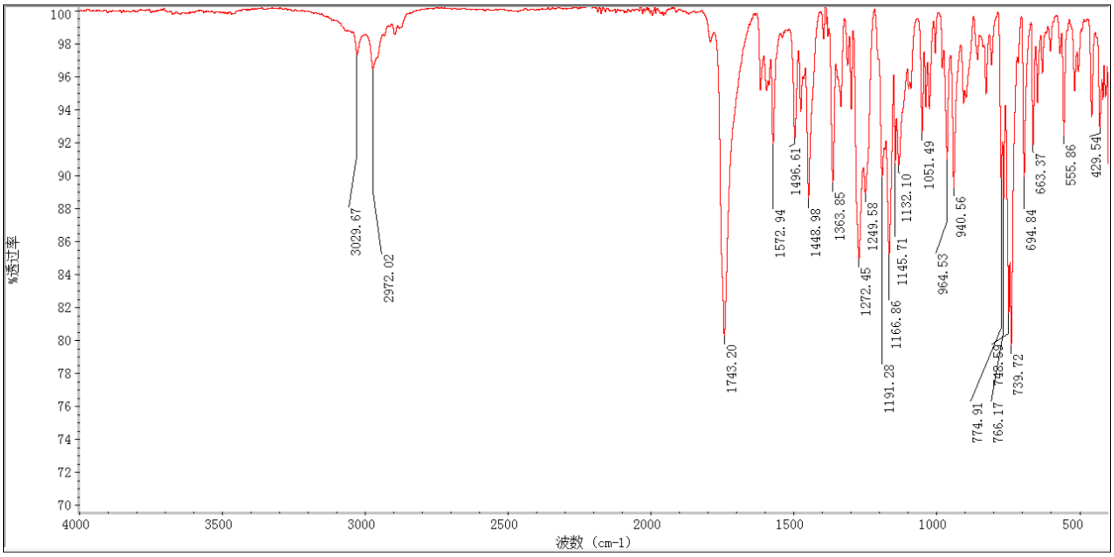

20. ^{1}H NMR spectra of compound **4f**

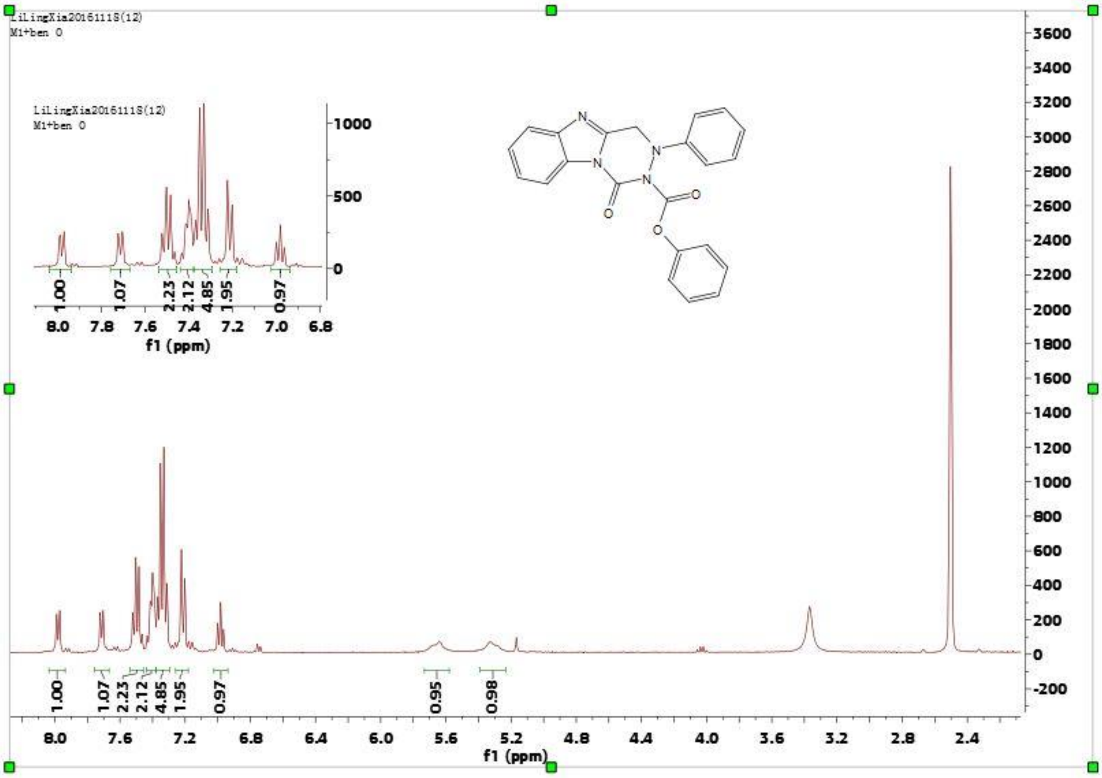

21. ^{13}C NMR spectra of compound **4f**

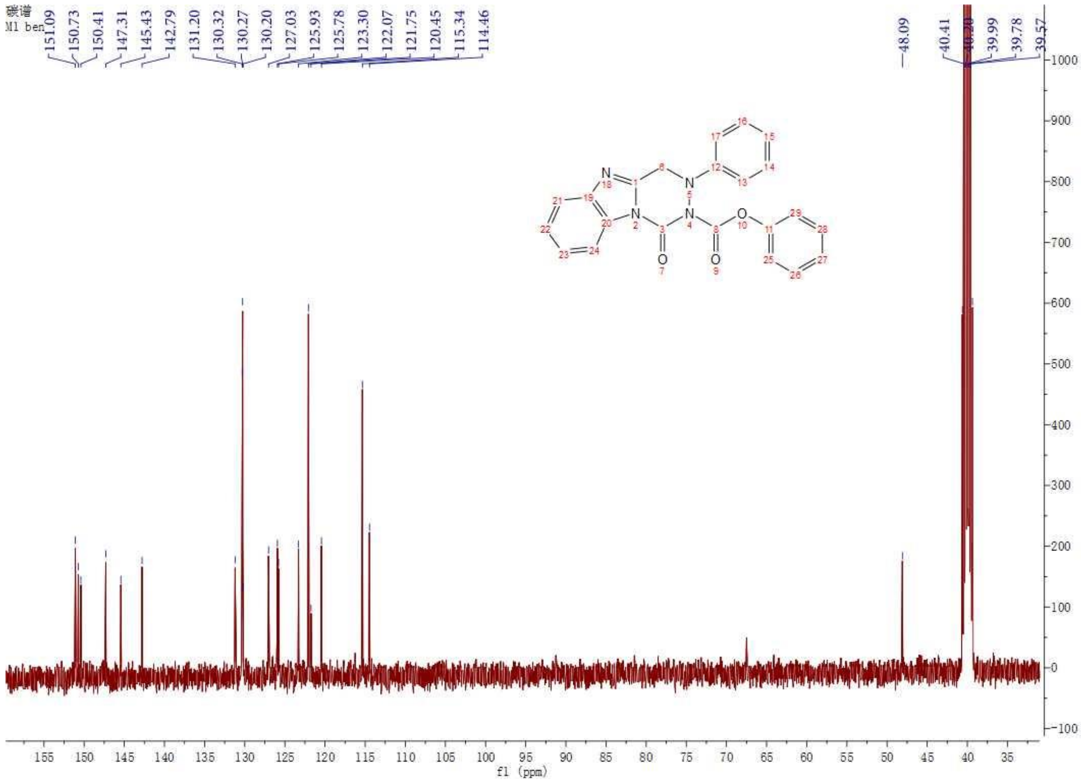

22. EI-MS spectra of compound **4f**

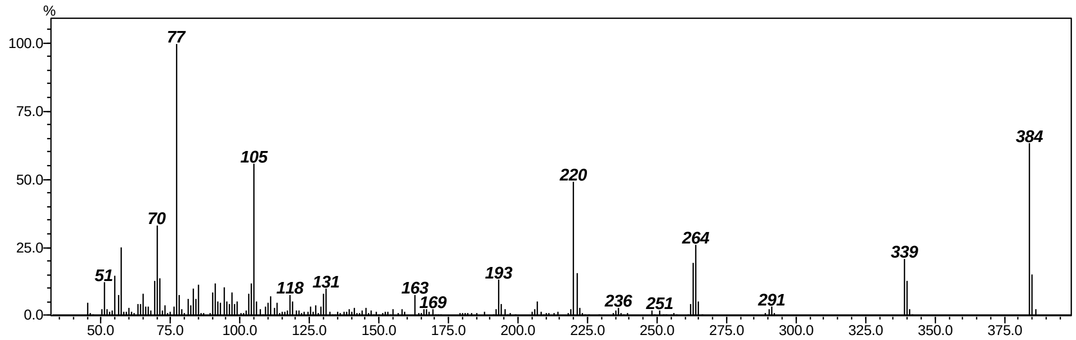

23. FT-IR spectra of compound **4f**

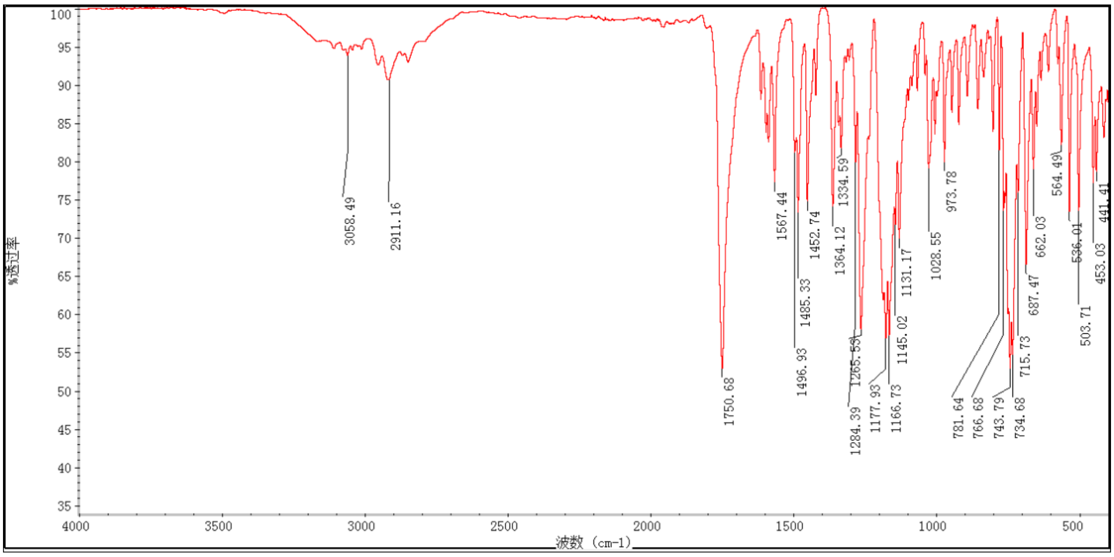

24. ^{1}H NMR spectra of compound **4g**

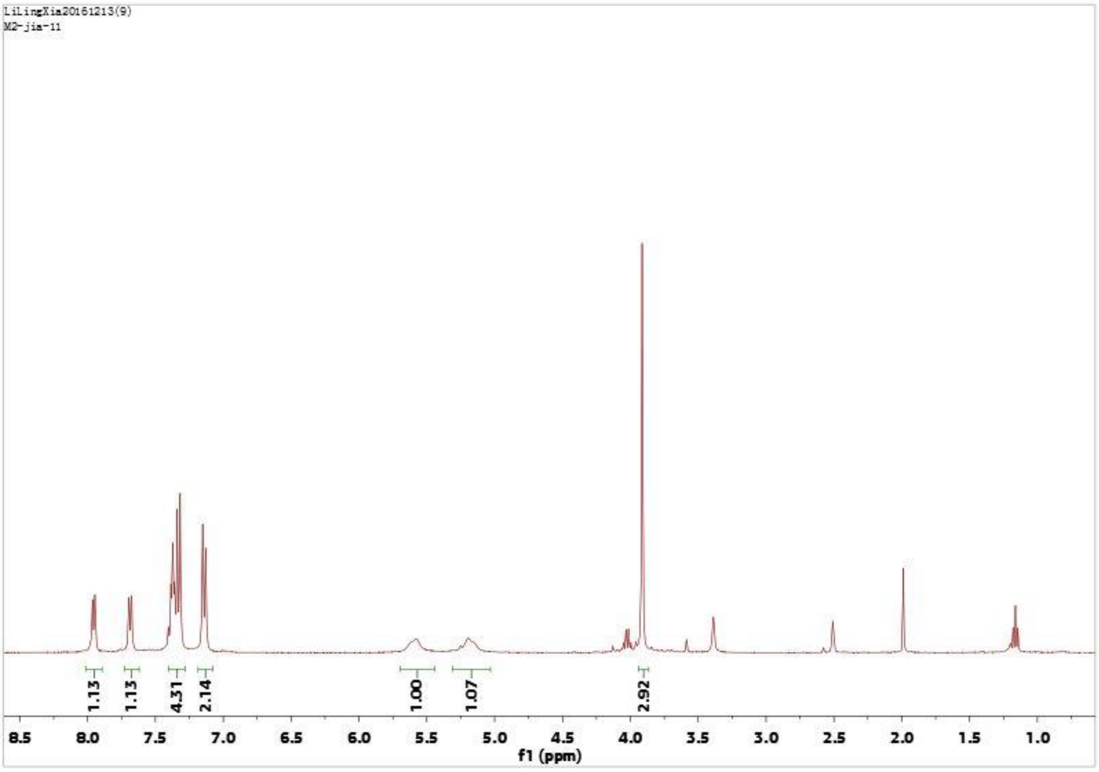

25. ^{13}C NMR spectra of compound **4g**

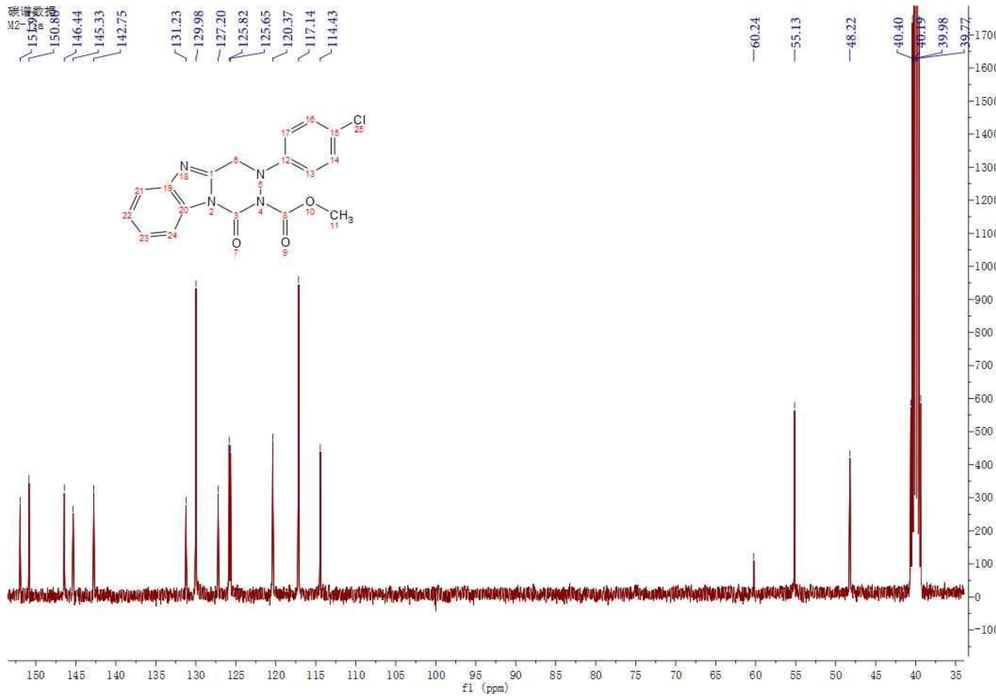

26. EI-MS spectra of compound **4g**

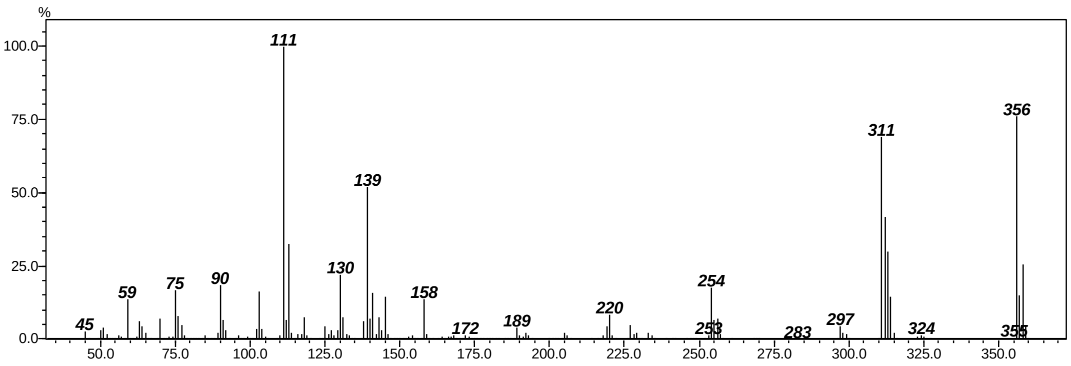

27. FT-IR spectra of compound **4g**

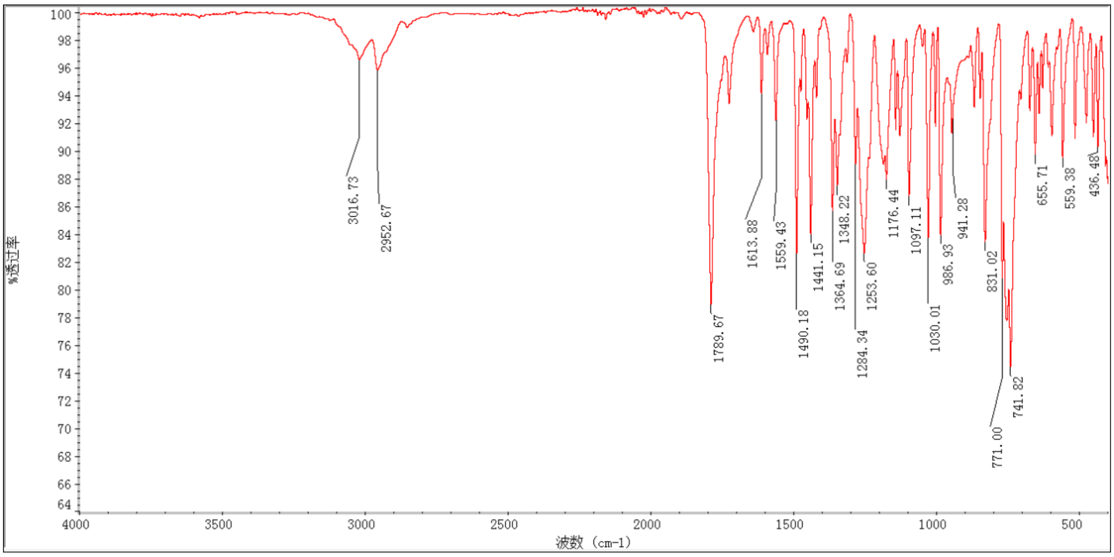

28. ^{1}H NMR spectra of compound **4h**

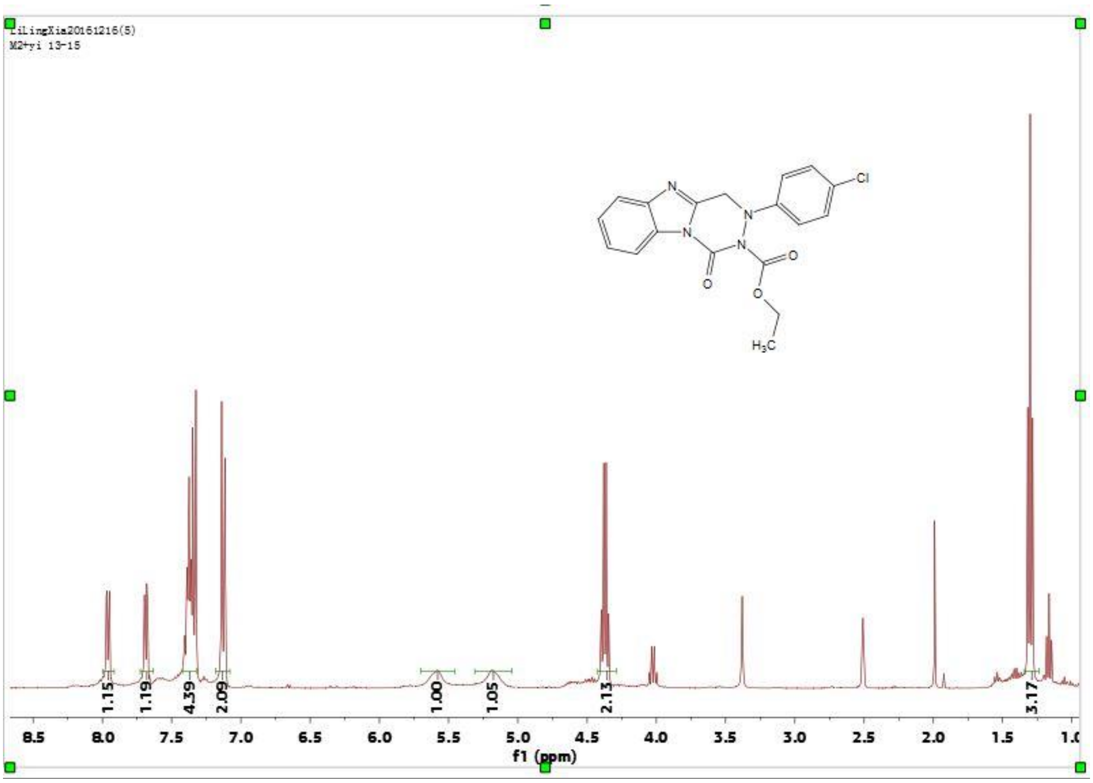

29. ^{13}C NMR spectra of compound **4h**

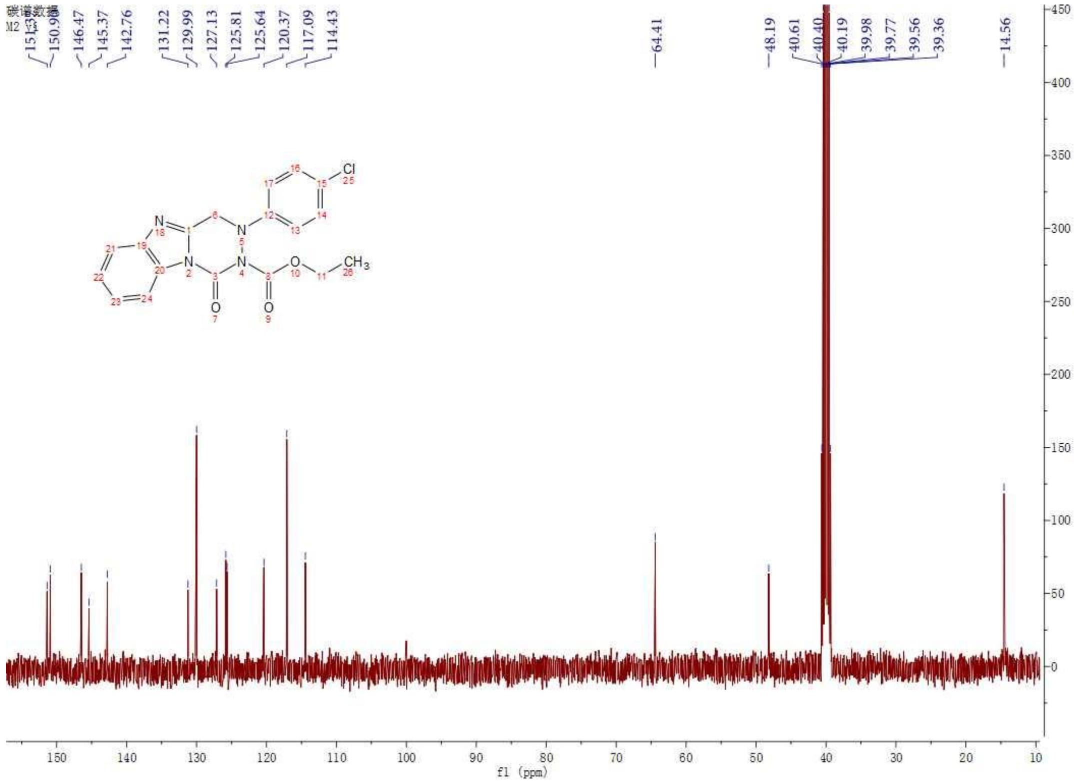

30. EI-MS spectra of compound **4h**

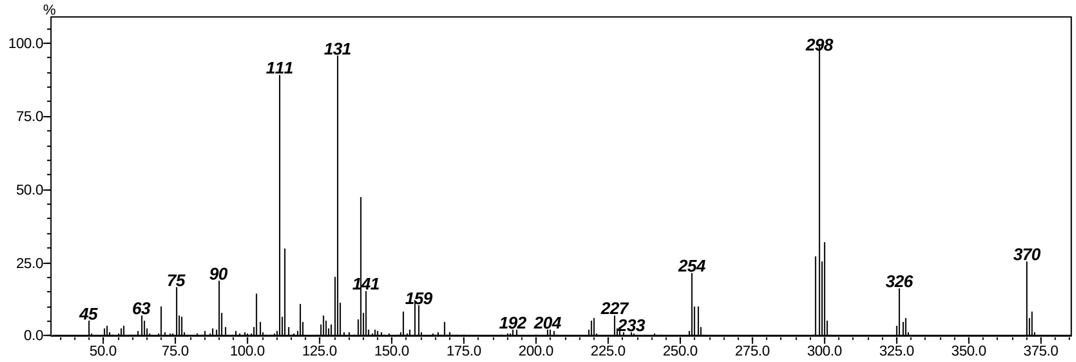

31. FT-IR spectra of compound **4h**

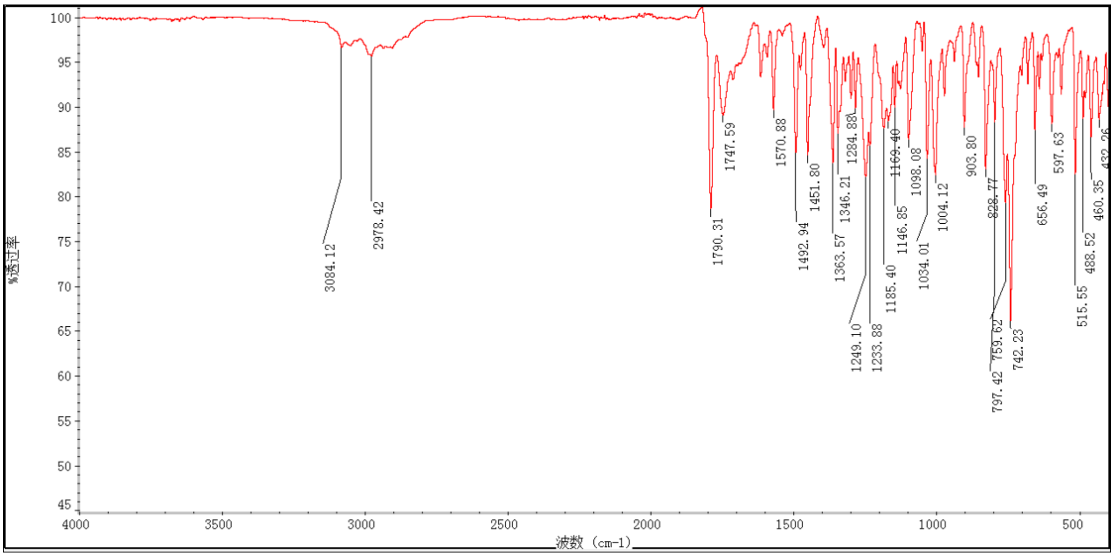

32. ^{1}H NMR spectra of compound **4i**

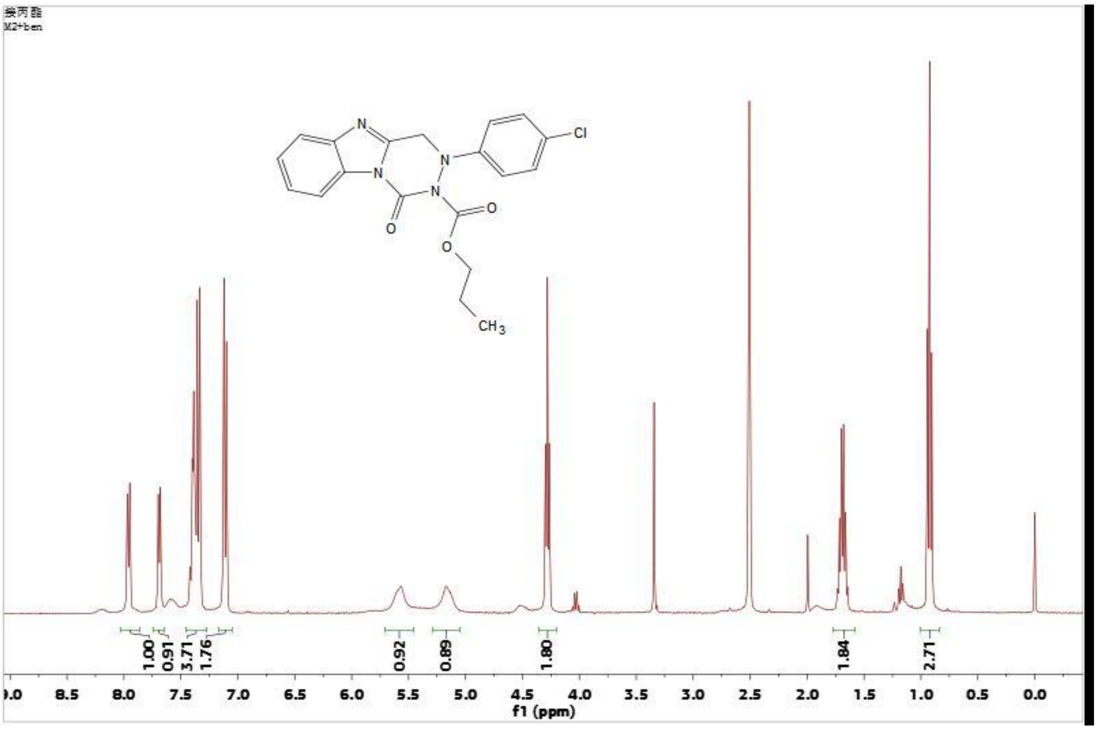

33. ^{13}C NMR spectra of compound **4i**

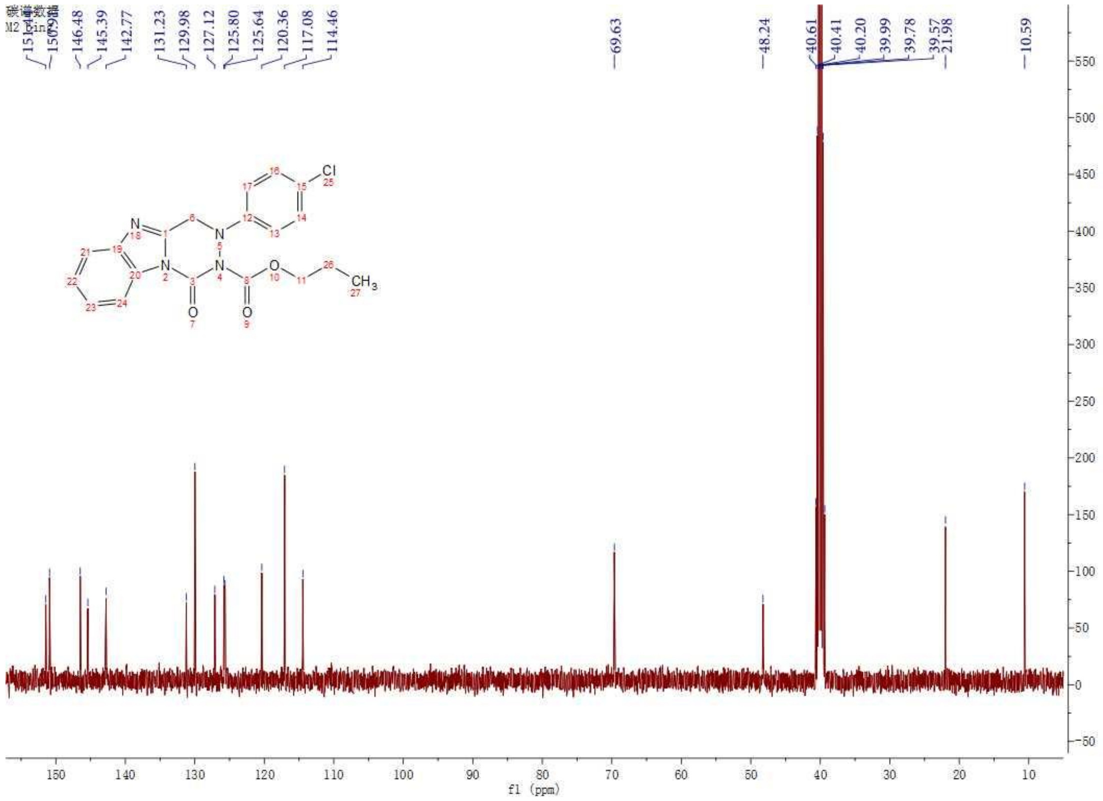

34. EI-MS spectra of compound **4i**

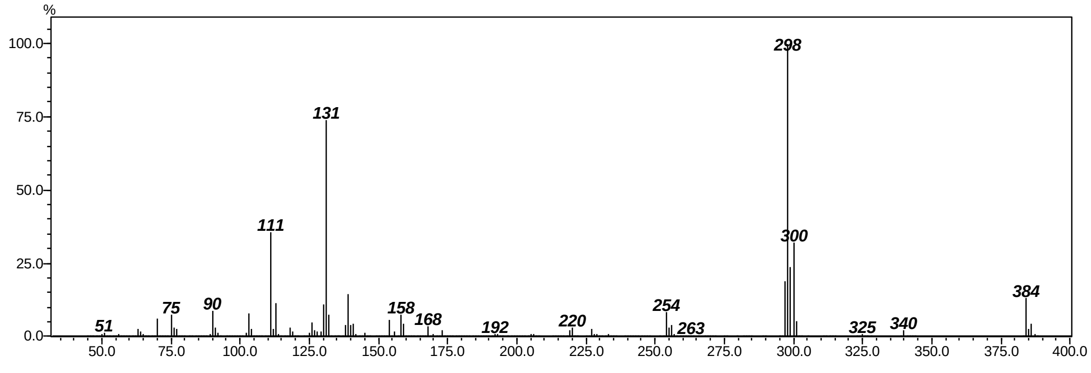

35. FT-IR spectra of compound **4i**

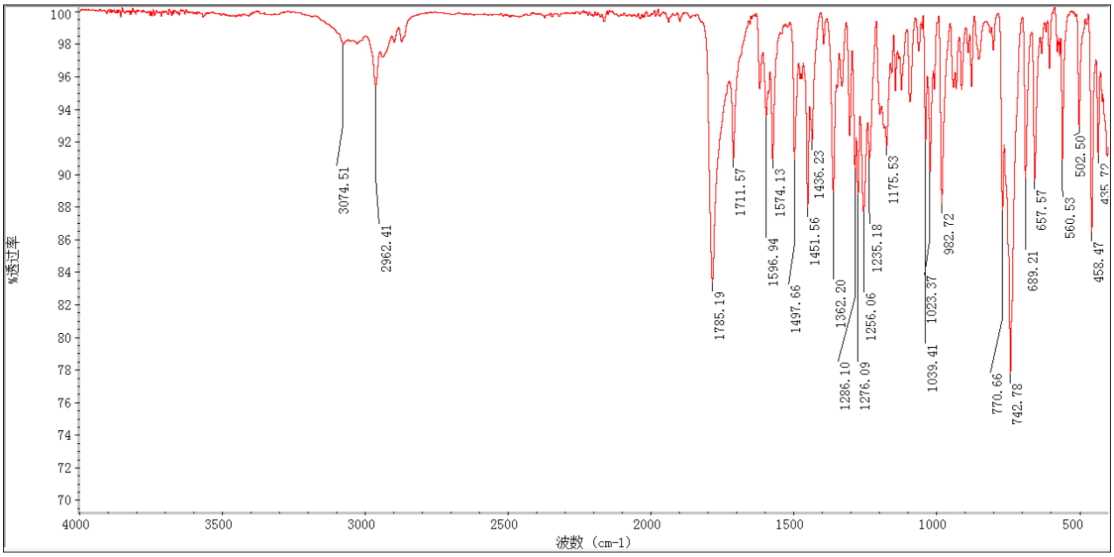

36. ^{1}H NMR spectra of compound **4j**

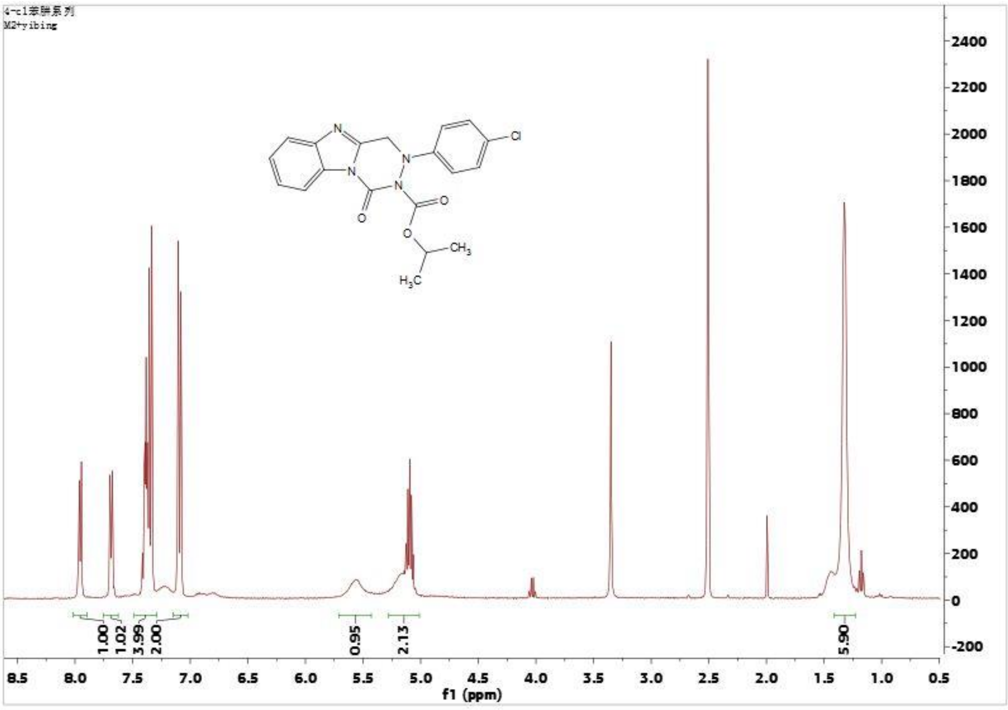

37. ^{13}C NMR spectra of compound **4j**

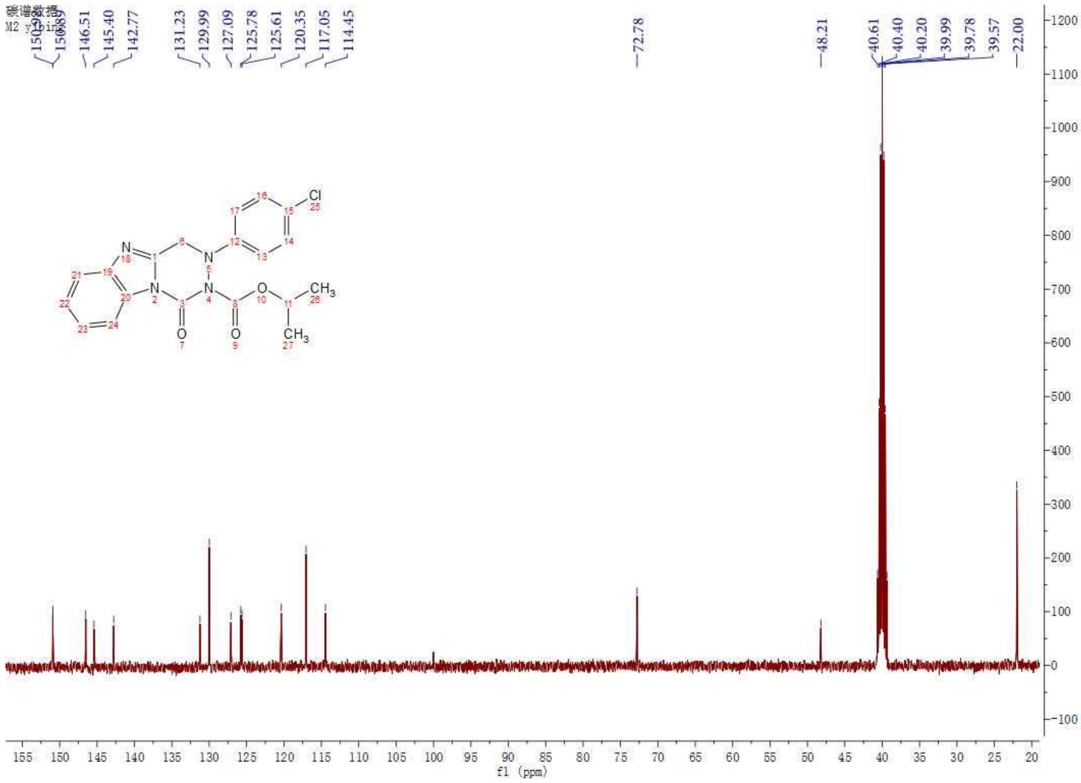

38. EI-MS spectra of compound **4j**

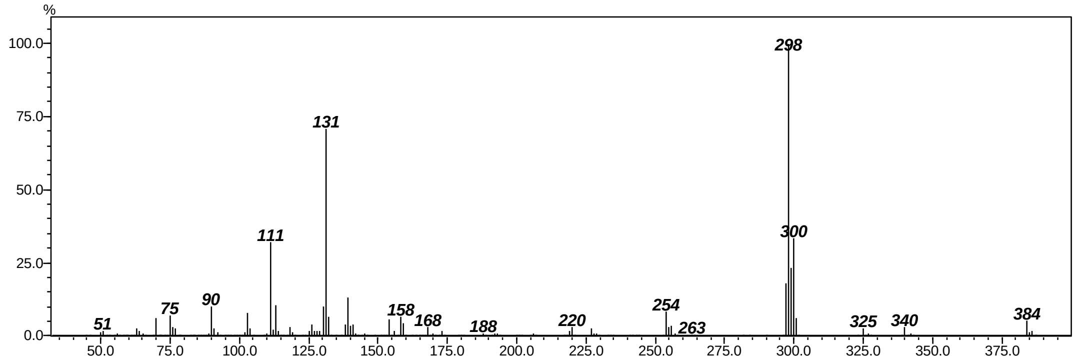

39. FT-IR spectra of compound **4j**

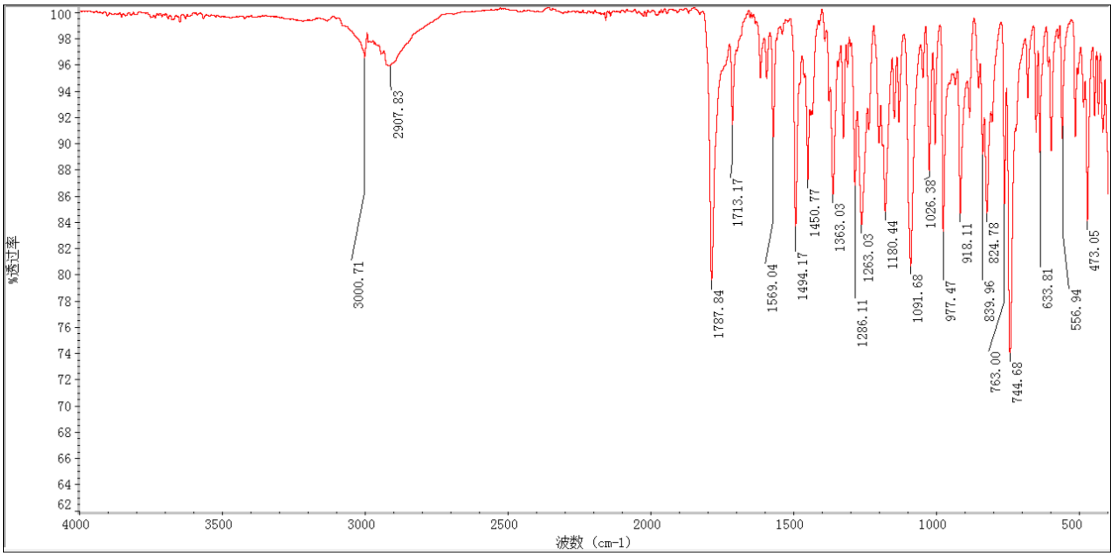

40. ¹H NMR spectra of compound **4k**

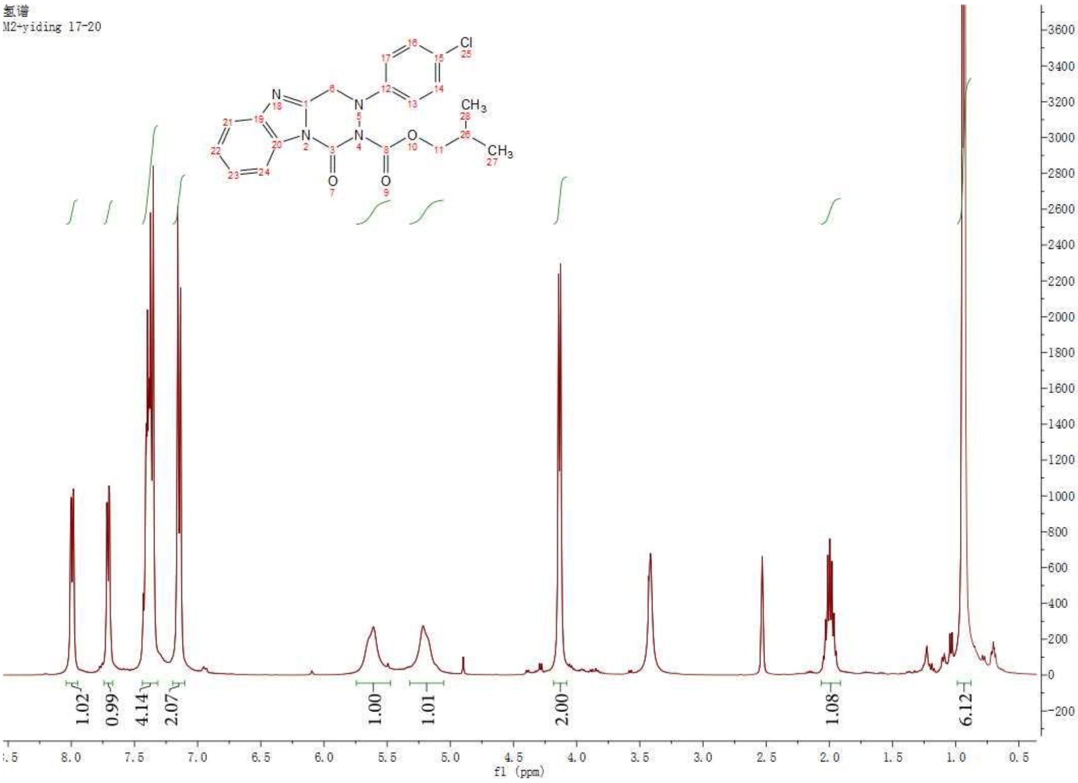

41. ^{13}C NMR spectra of compound **4k**

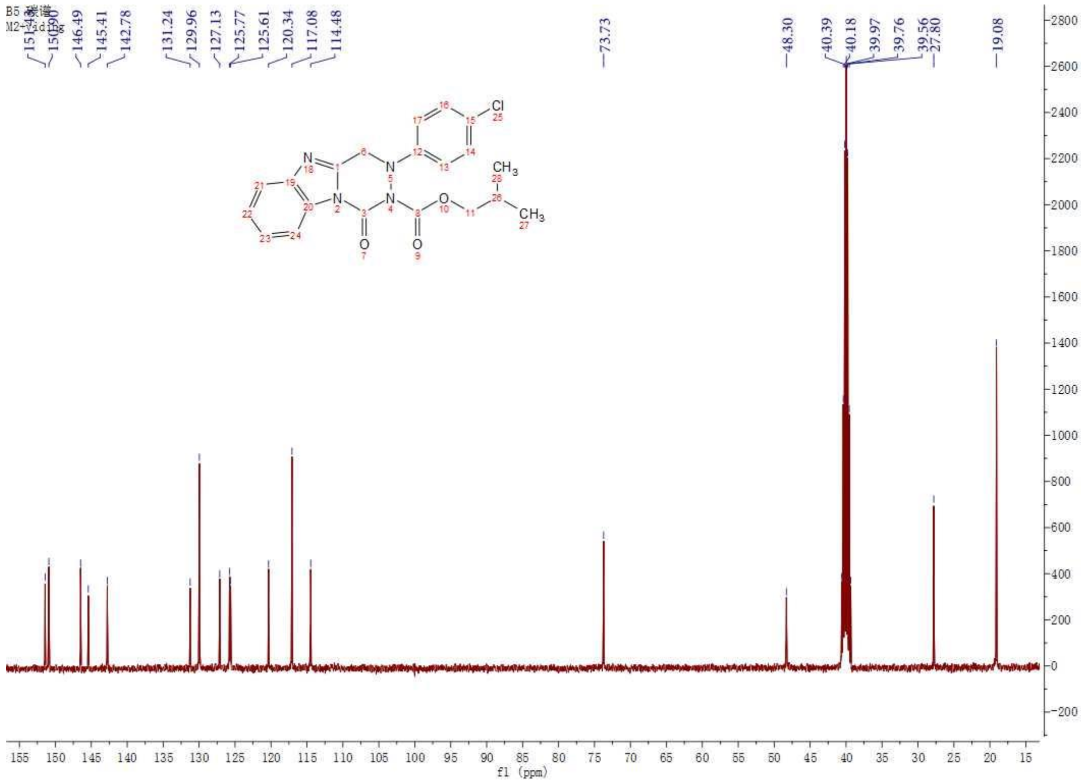

42. EI-MS spectra of compound **4k**

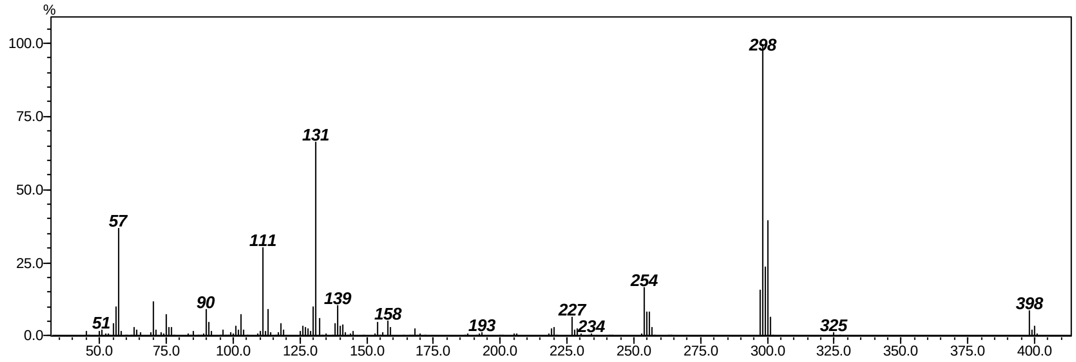

43. FT-IR spectra of compound **4k**

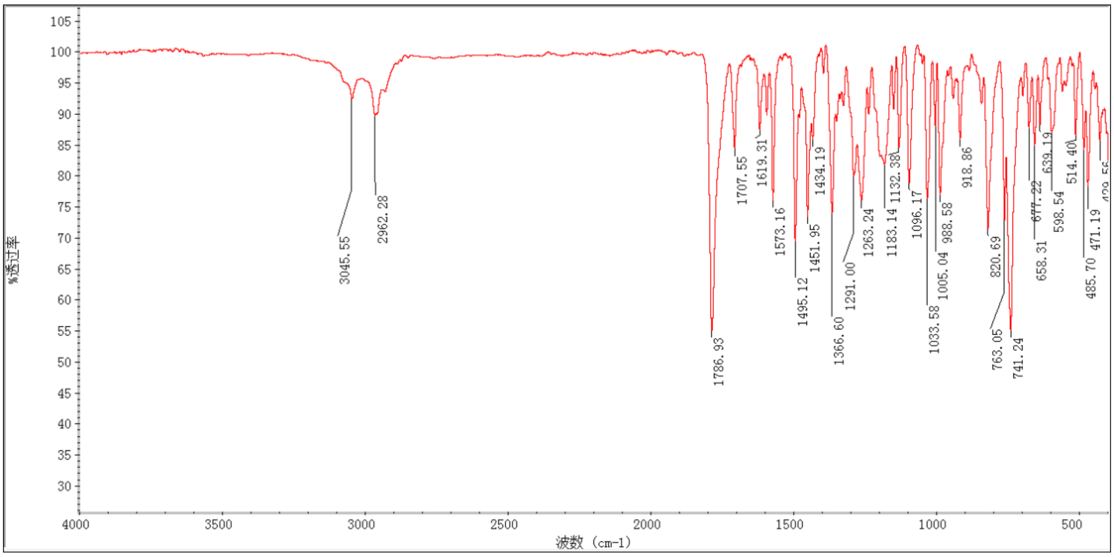

44. ^{1}H NMR spectra of compound **4l**

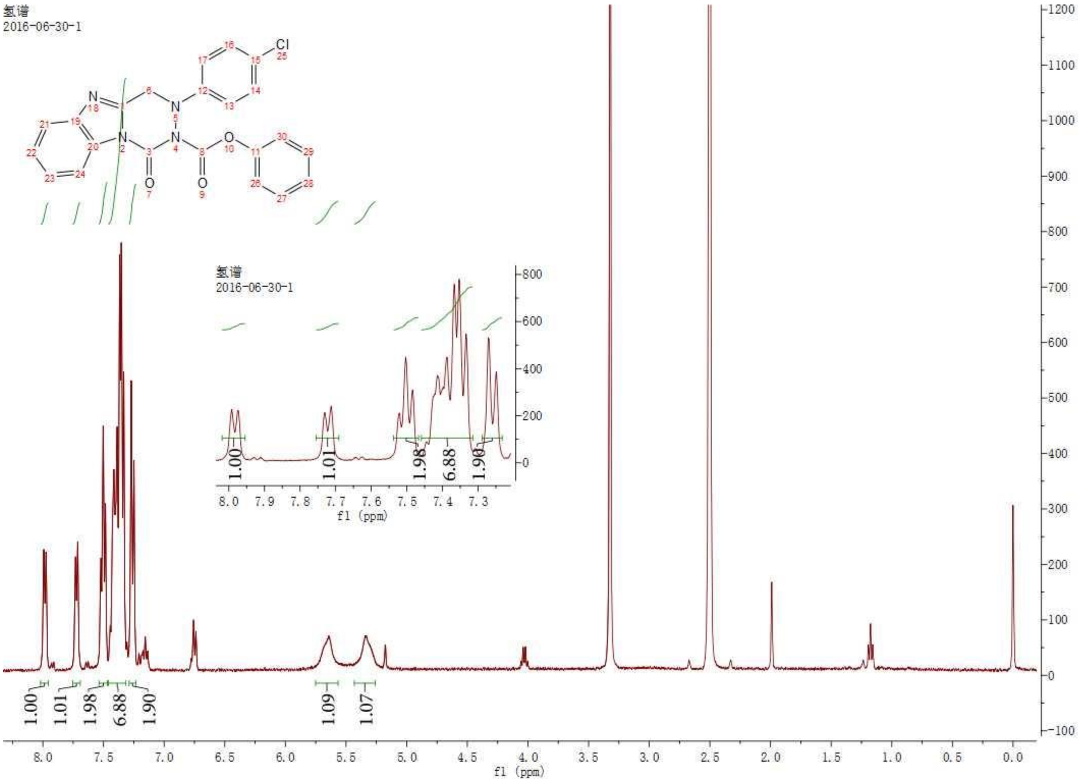

45. ^{13}C NMR spectra of compound **4l**

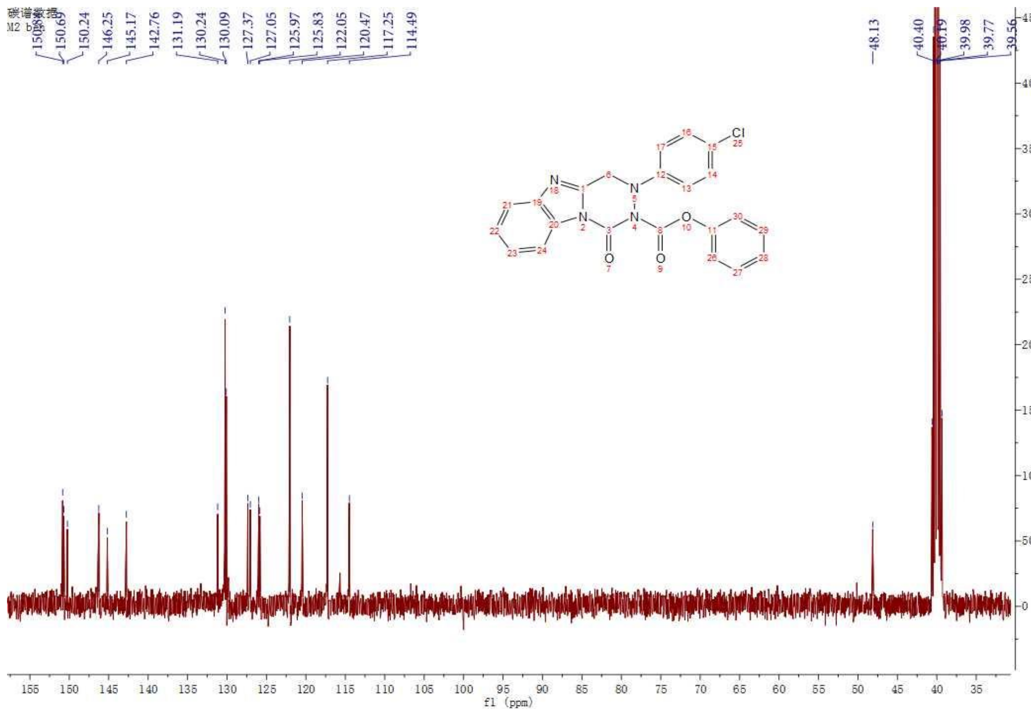

46. EI-MS spectra of compound **4l**

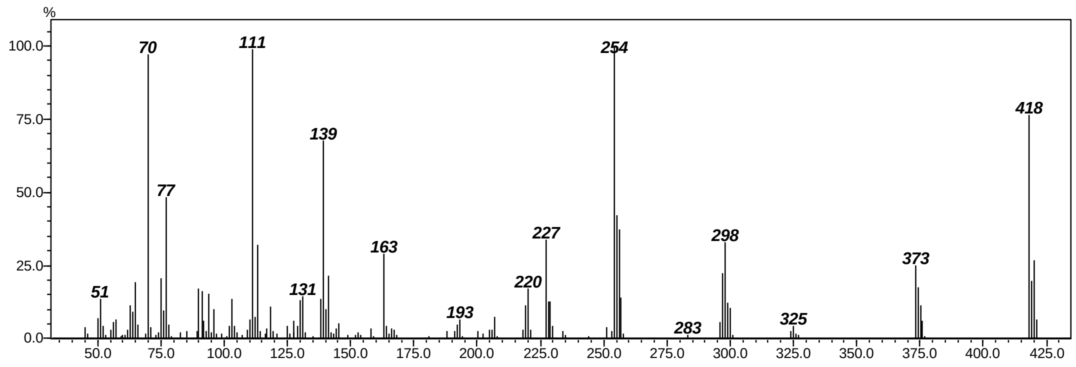

47. FT-IR spectra of compound **4l**

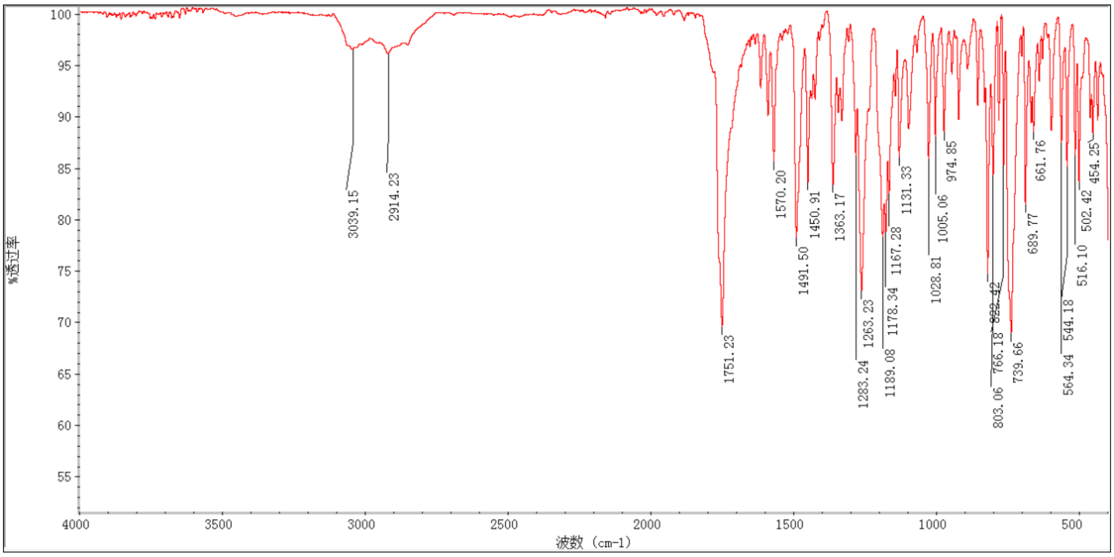

48. ^{1}H NMR spectra of compound **4m**

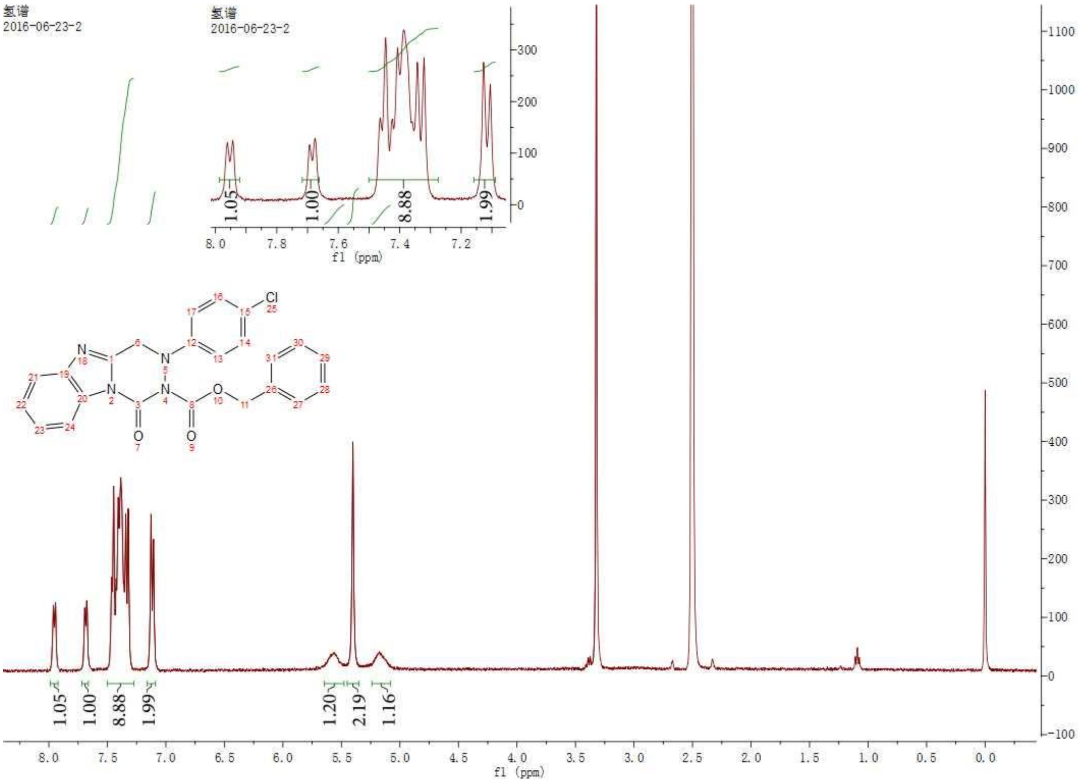

49. ^{13}C NMR spectra of compound **4m**

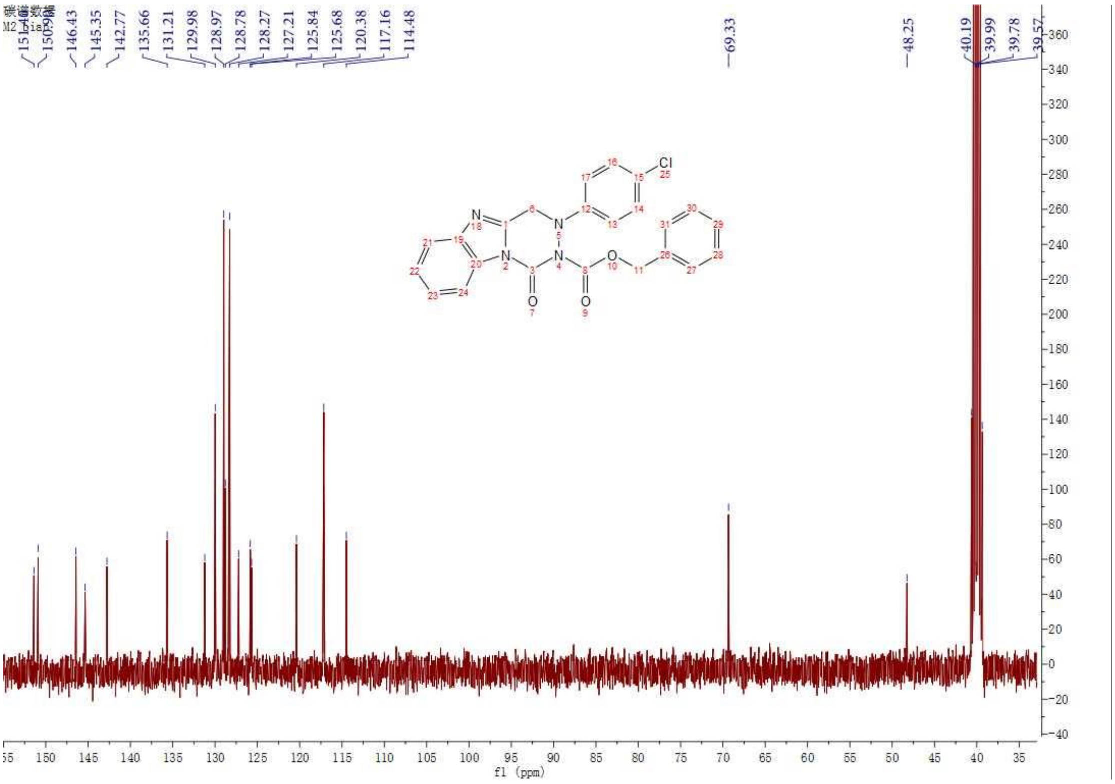

50. EI-MS spectra of compound **4m**

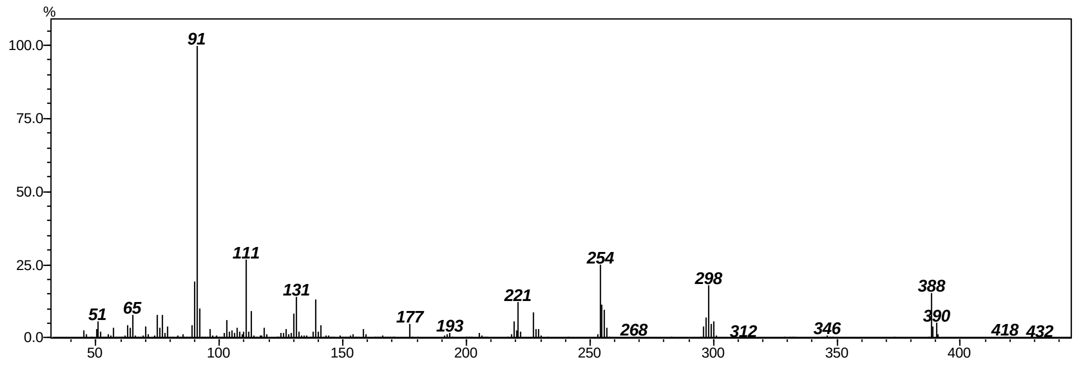

51. FT-IR spectra of compound **4m**

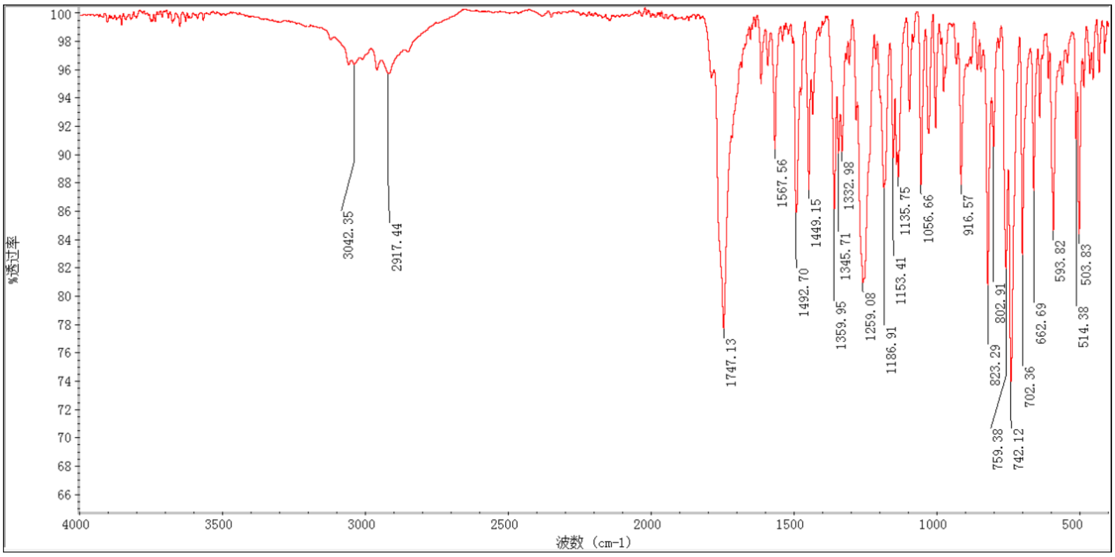

52. ^{1}H NMR spectra of compound **4n**

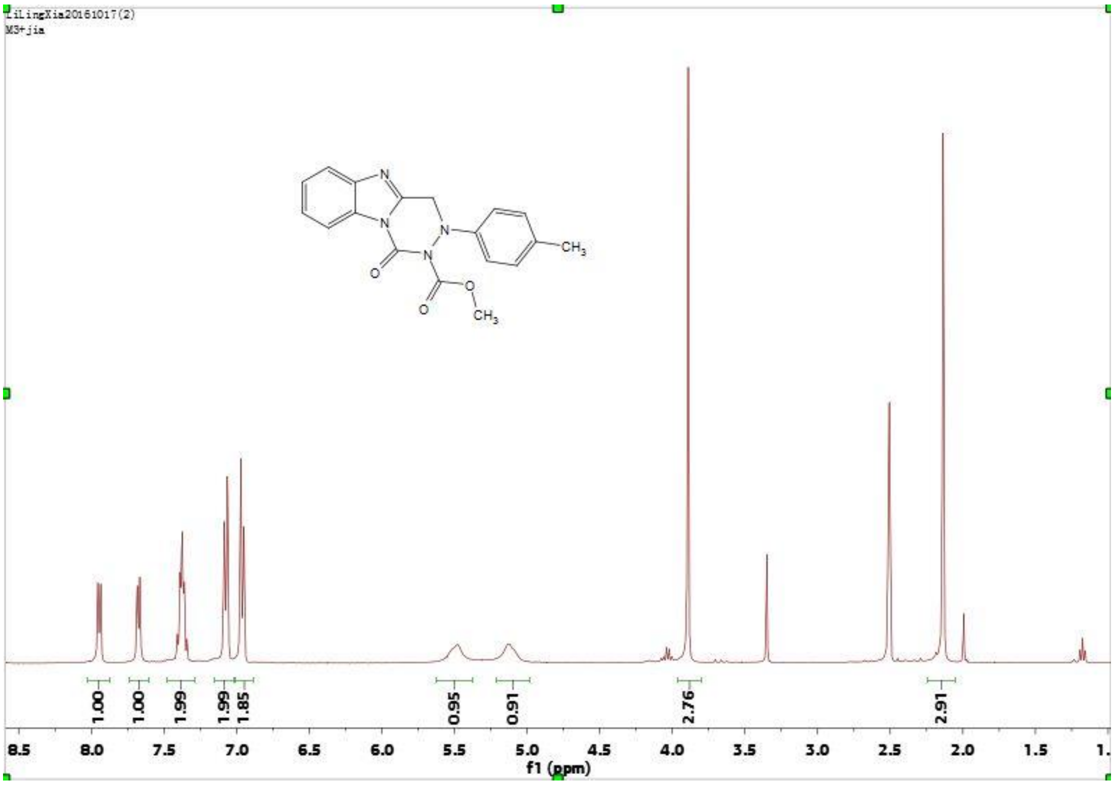

53. ^{13}C NMR spectra of compound **4n**

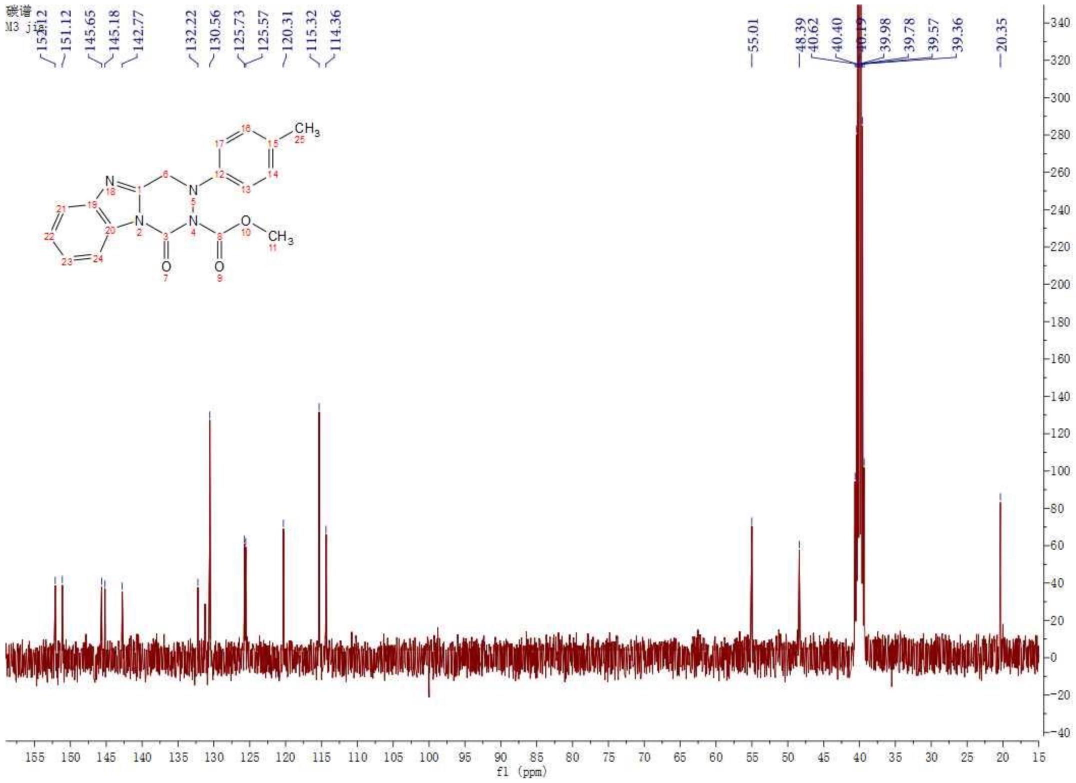

54. EI-MS spectra of compound **4n**

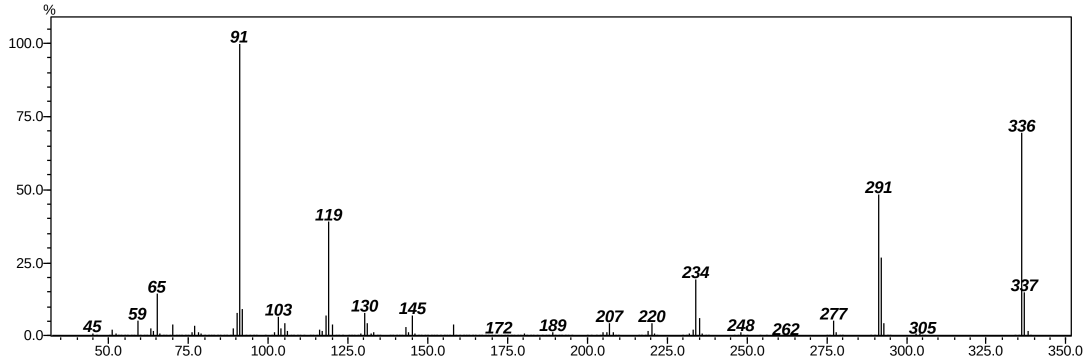

55. FT-IR spectra of compound **4n**

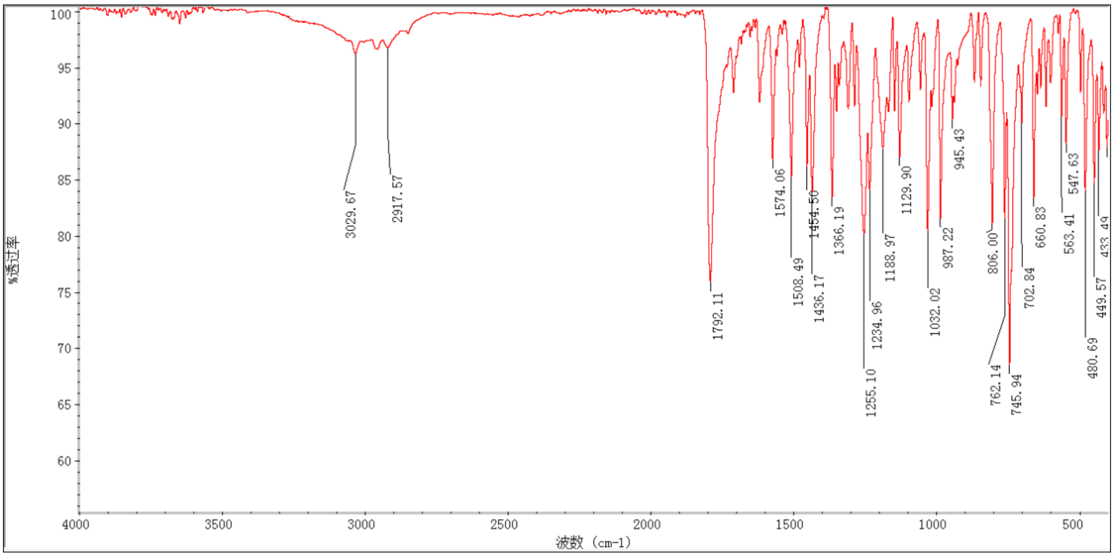

56. ^{1}H NMR spectra of compound **4o**

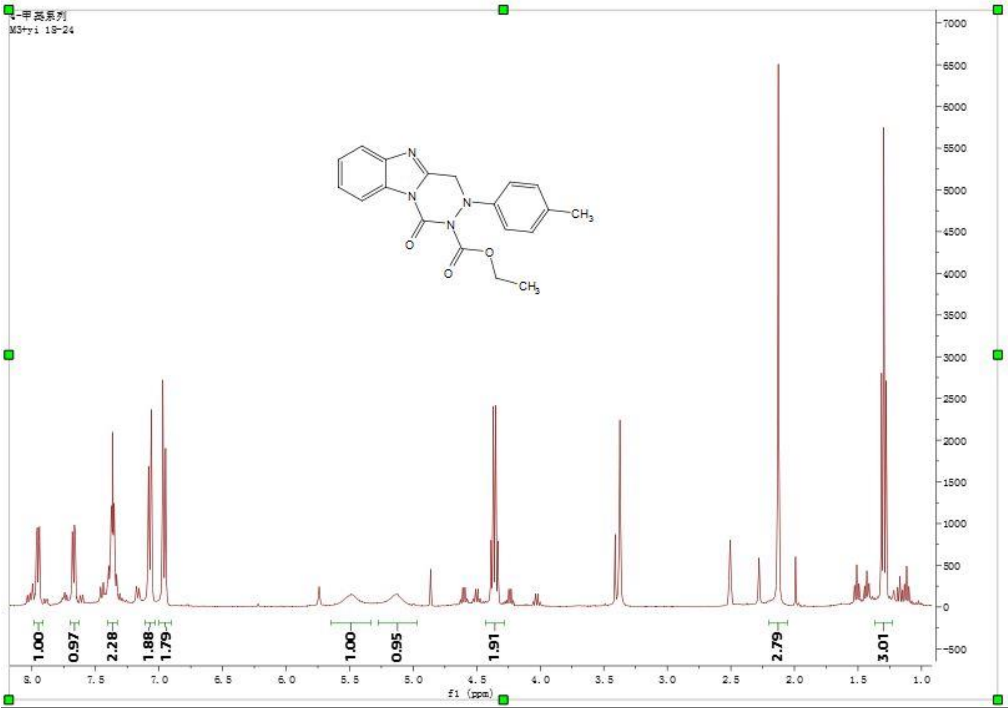

57. ^{13}C NMR spectra of compound **4o**

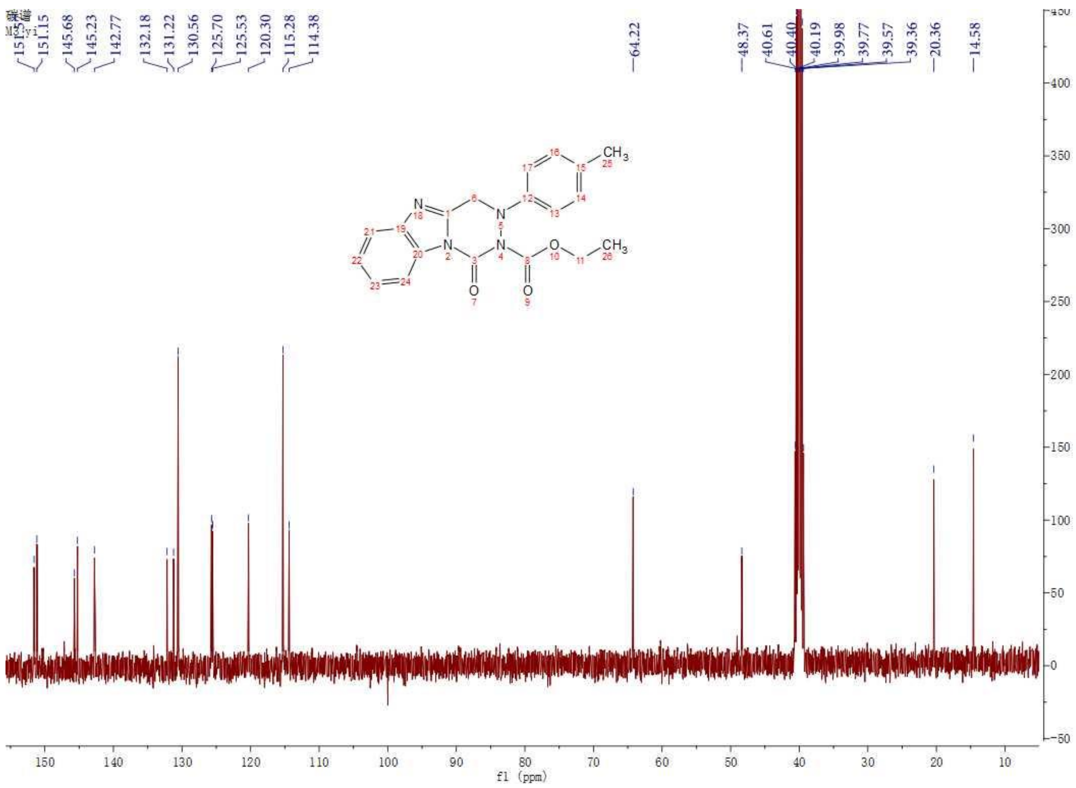

58. EI-MS spectra of compound **4o**

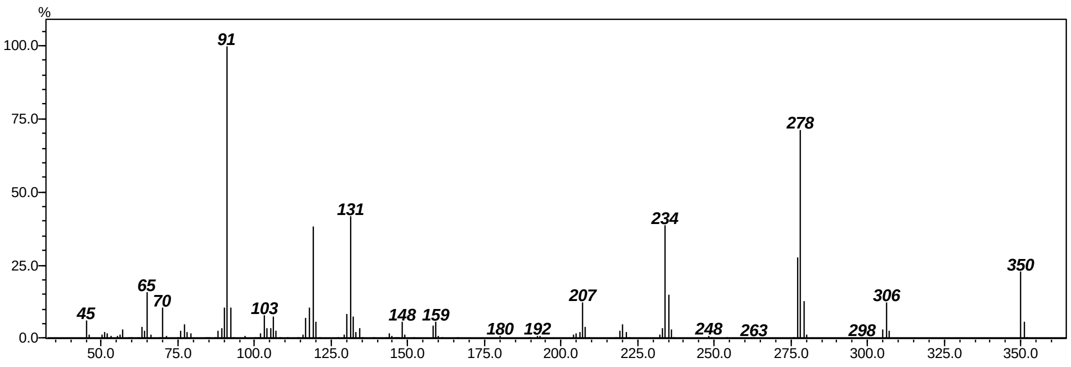

59. FT-IR spectra of compound **4o**

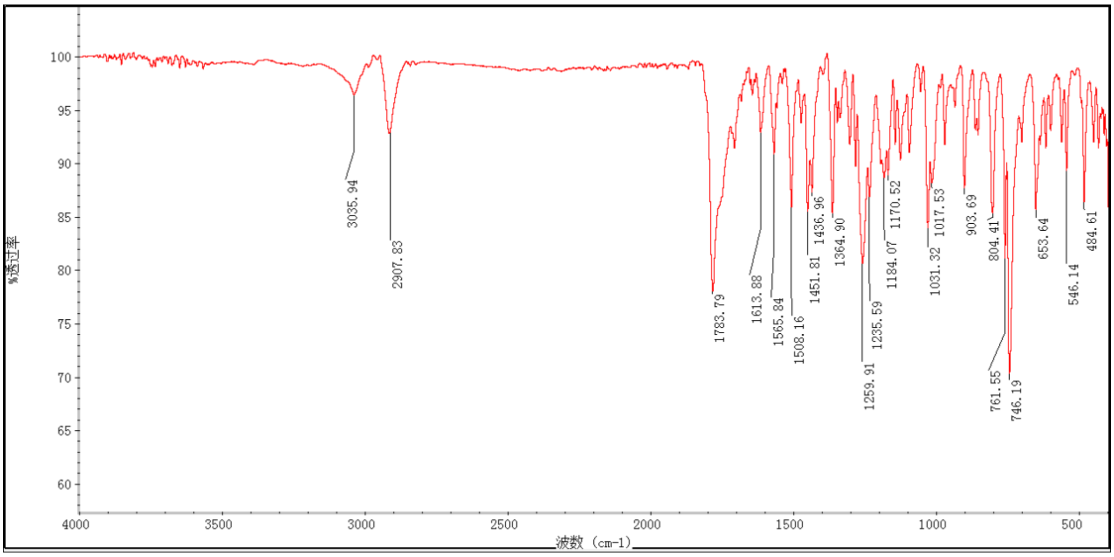

60. ^{1}H NMR spectra of compound **4p**

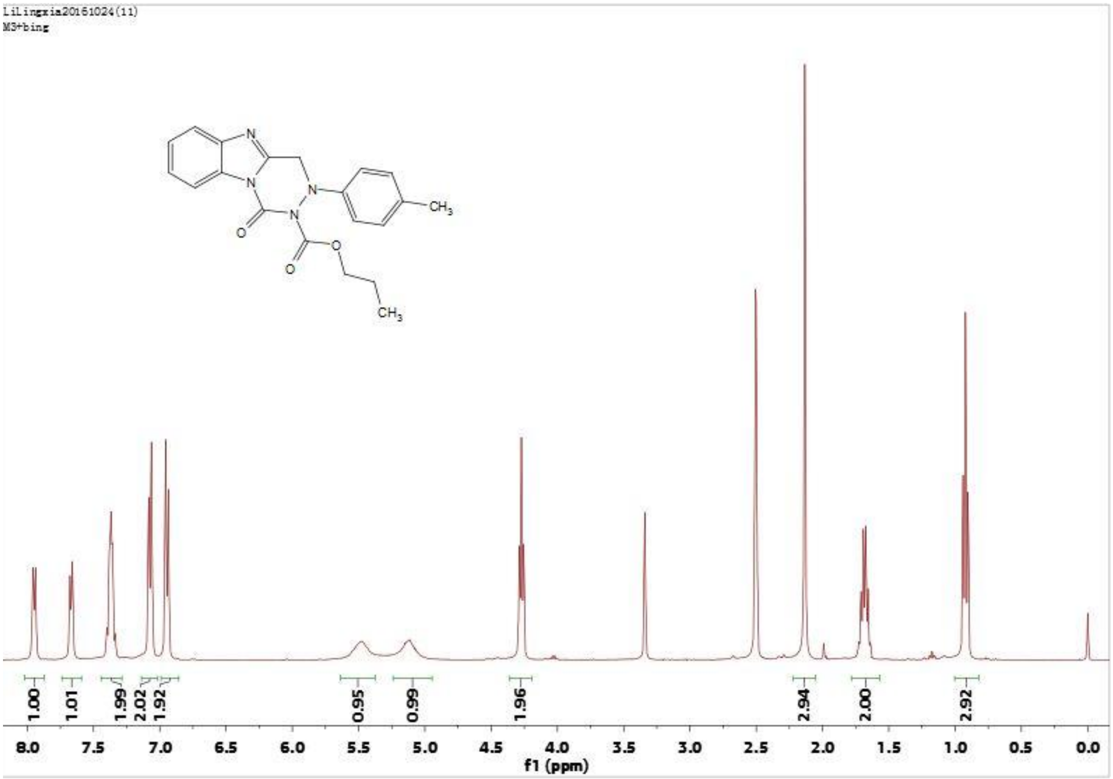

61. ^{13}C NMR spectra of compound **4p**

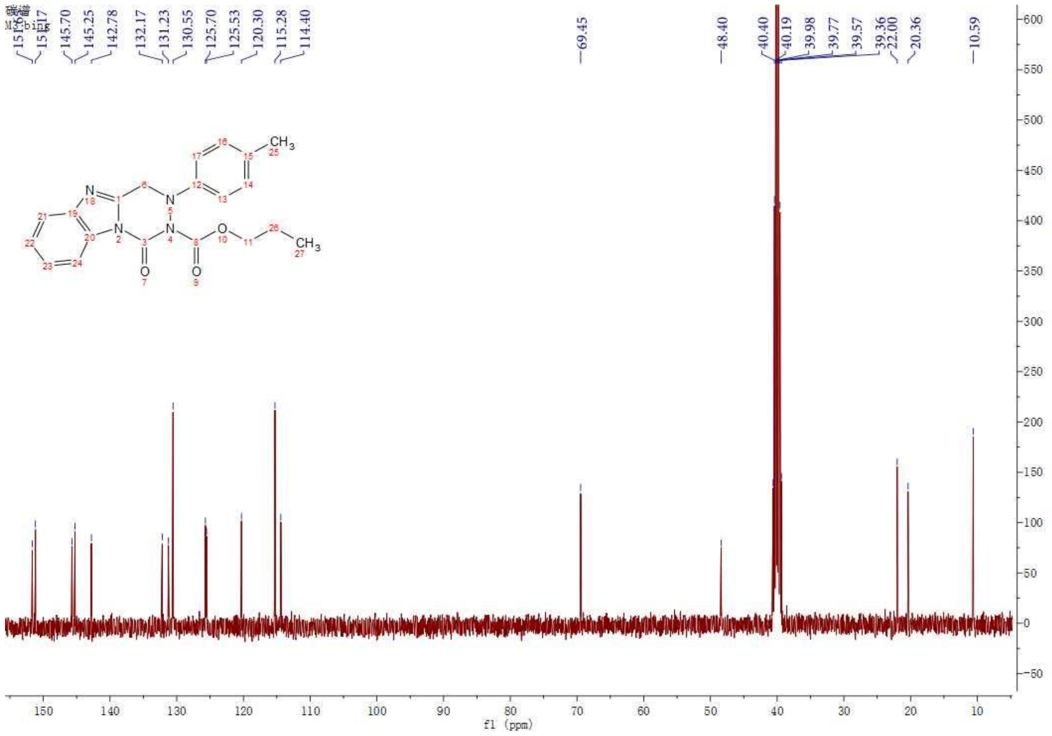

62. EI-MS spectra of compound **4p**

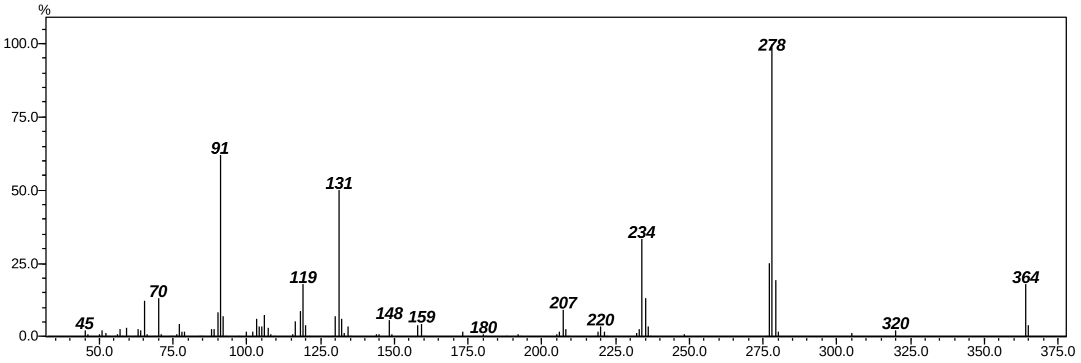

63. FT-IR spectra of compound **4p**

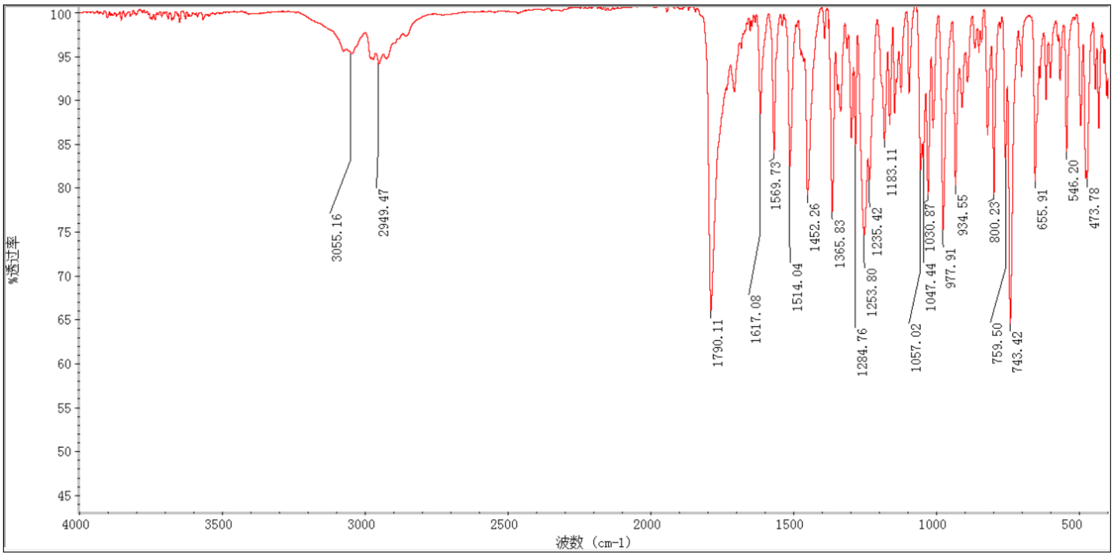

64. ^{1}H NMR spectra of compound **4q**

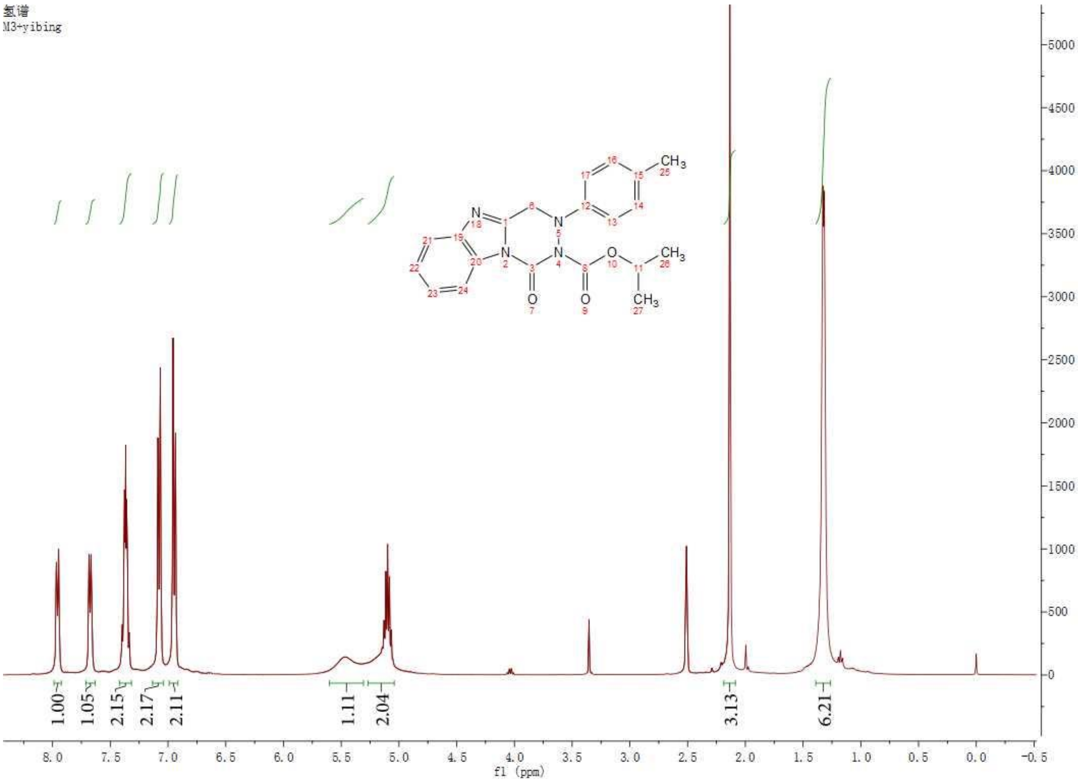

65. ^{13}C NMR spectra of compound **4q**

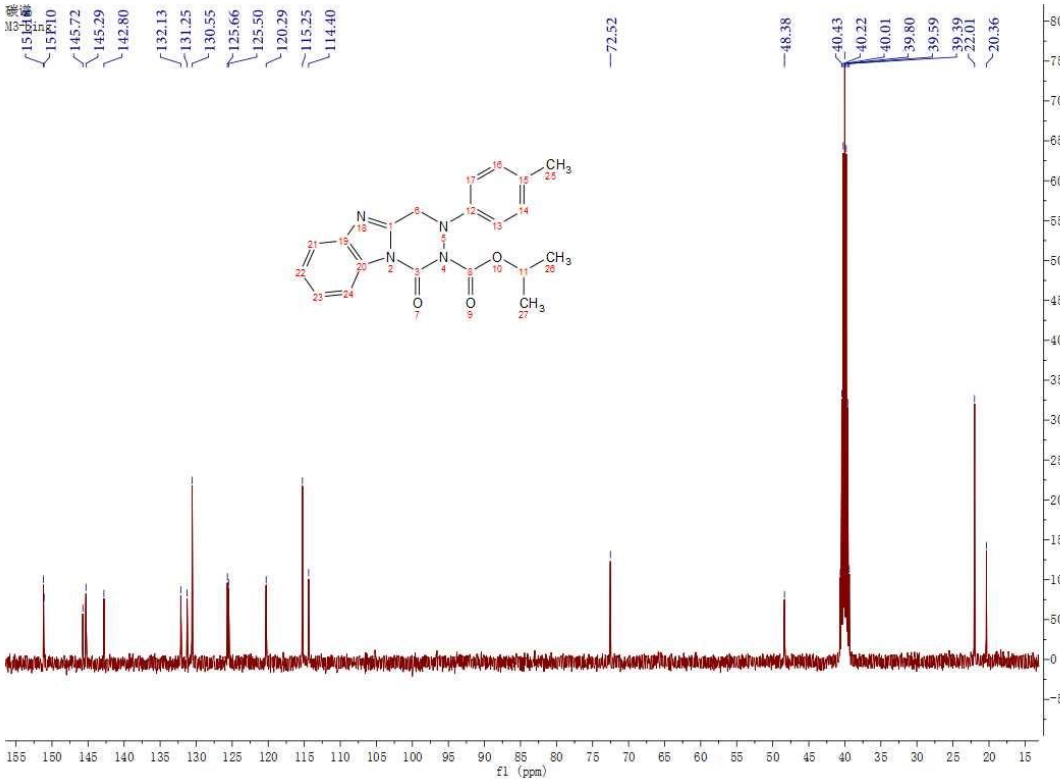

66. EI-MS spectra of compound **4q**

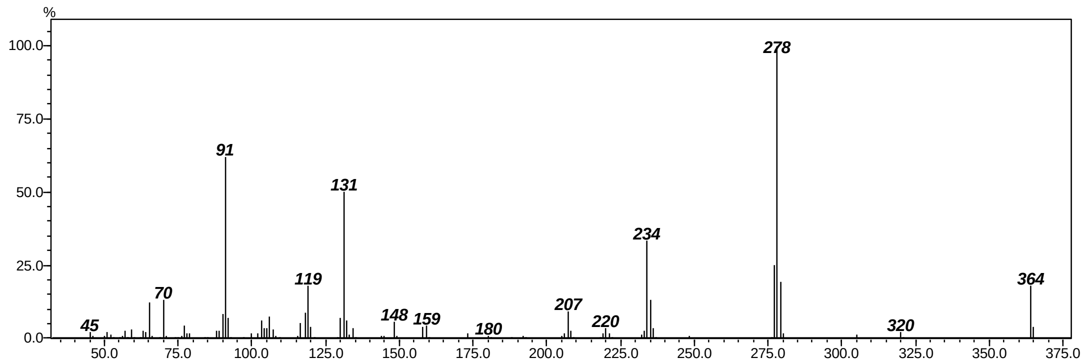

67. FT-IR spectra of compound **4q**

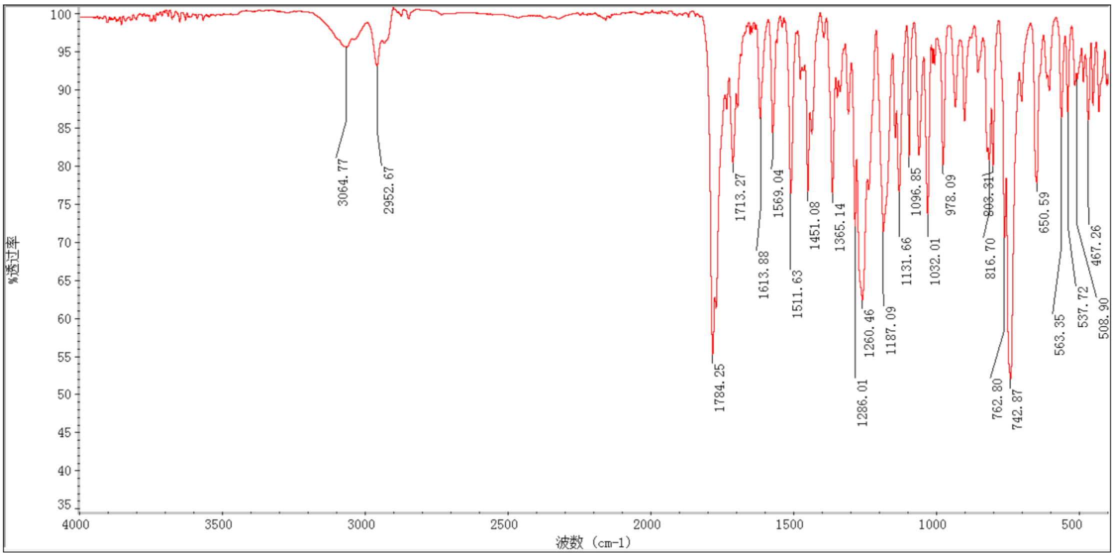

68. ^{1}H NMR spectra of compound **4r**

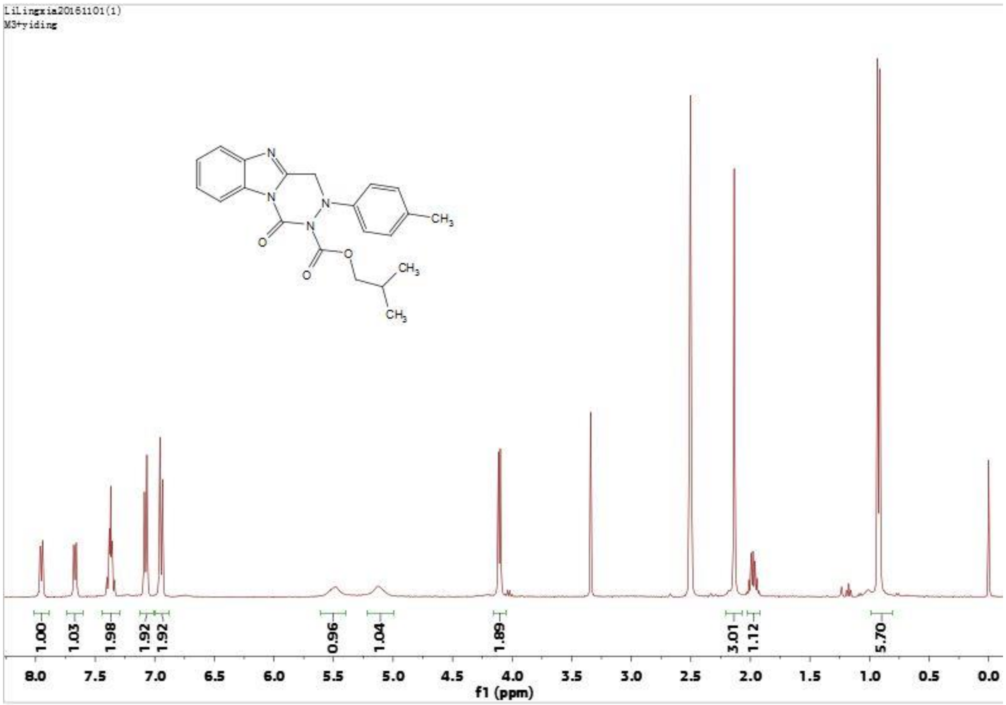

69. ^{13}C NMR spectra of compound **4r**

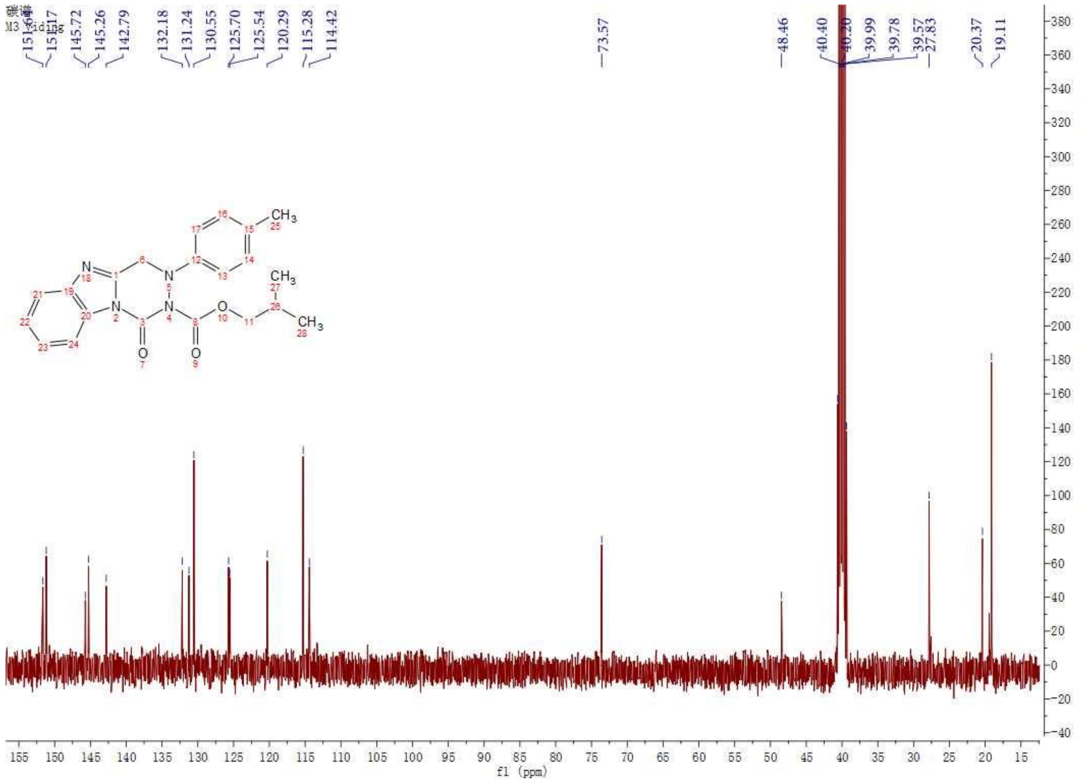

70. EI-MS spectra of compound **4r**

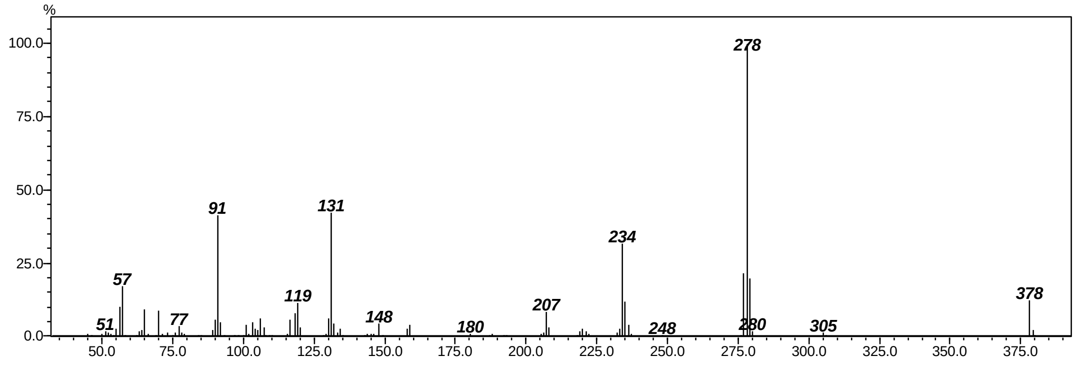

71. FT-IR spectra of compound **4r**

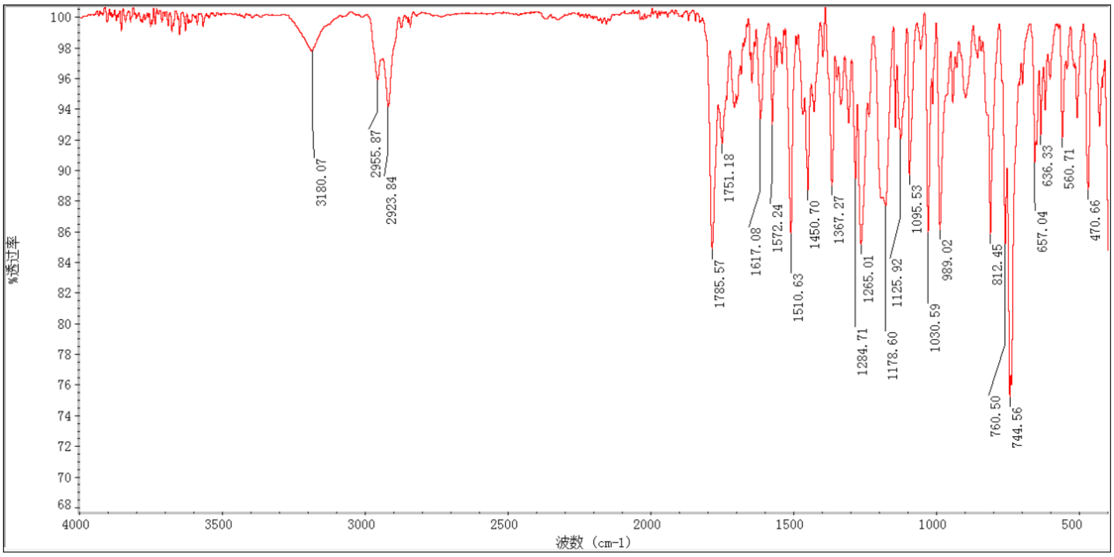

72. ^{1}H NMR spectra of compound **4s**

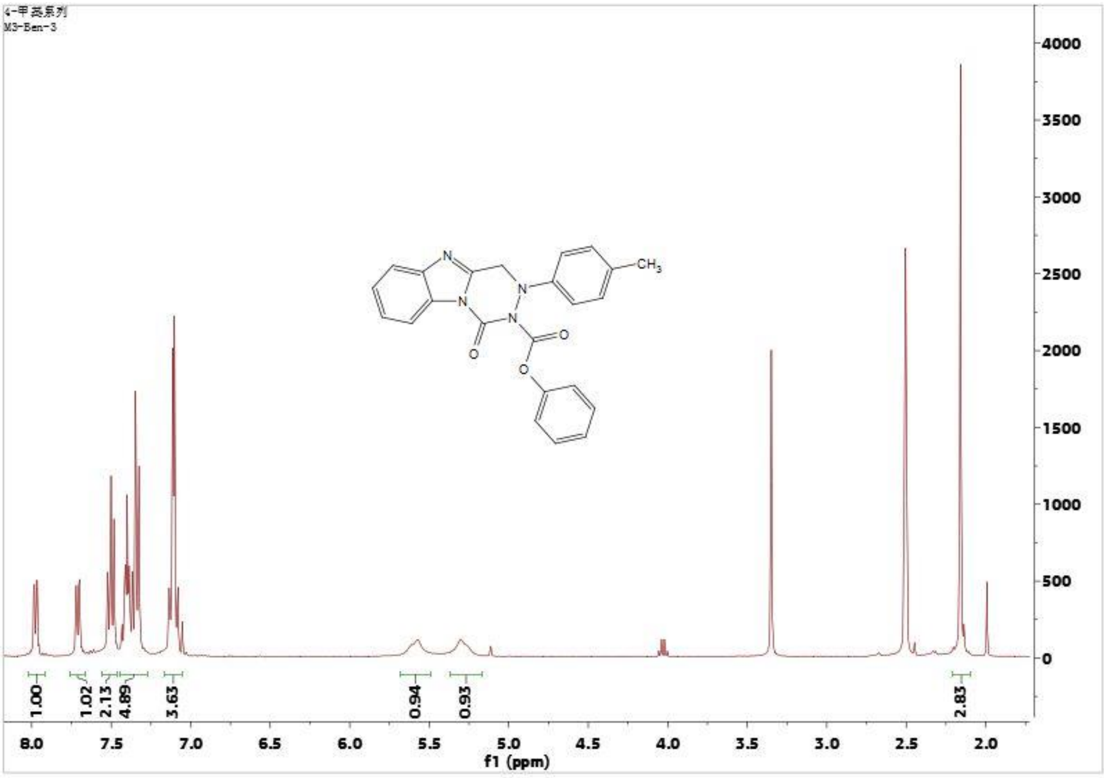

73. ^{13}C NMR spectra of compound **4s**

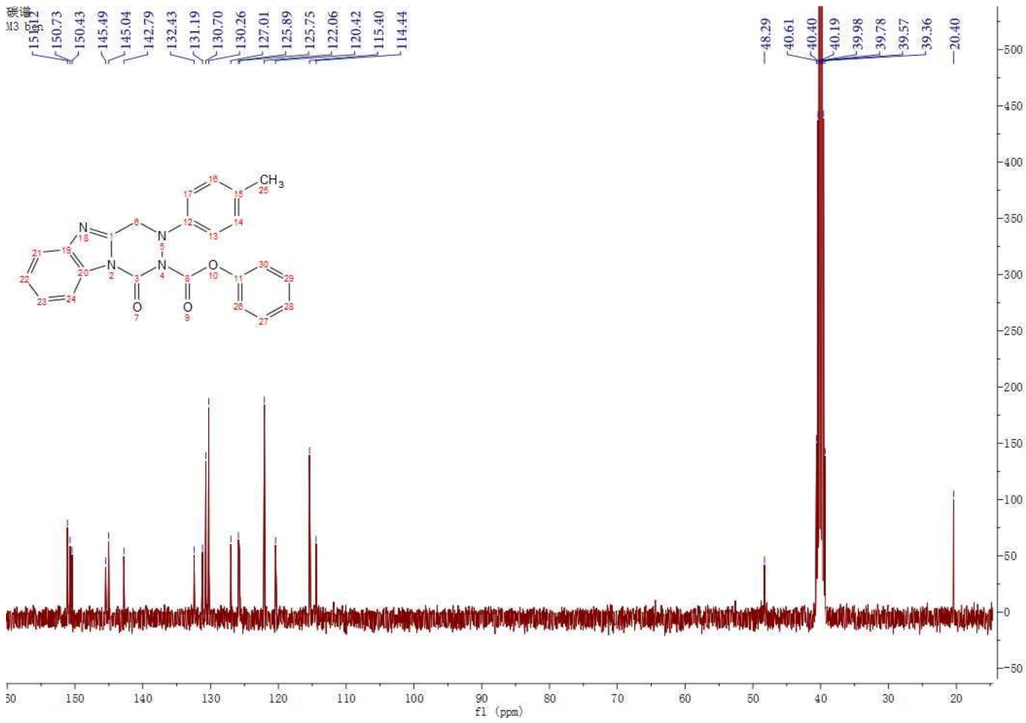

74. EI-MS spectra of compound **4s**

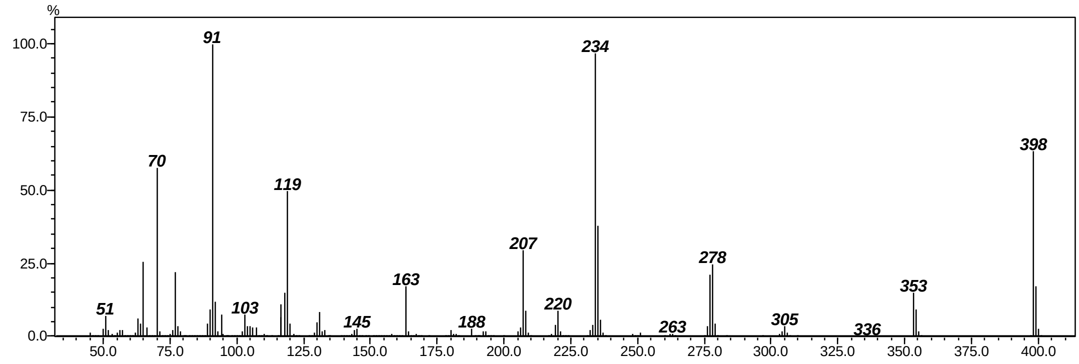

75. FT-IR spectra of compound **4s**

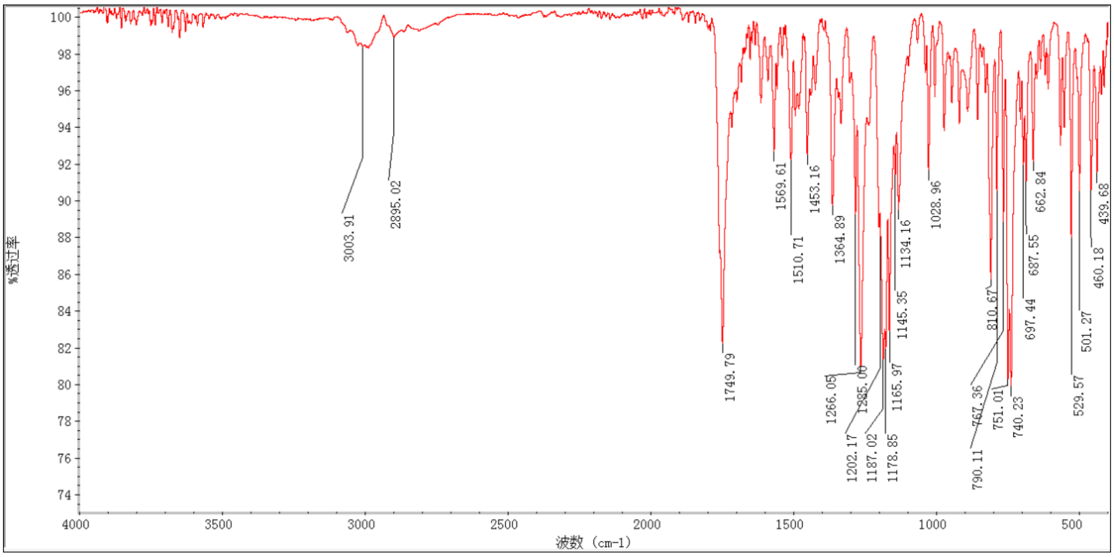

76. ^{1}H NMR spectra of compound **4t**

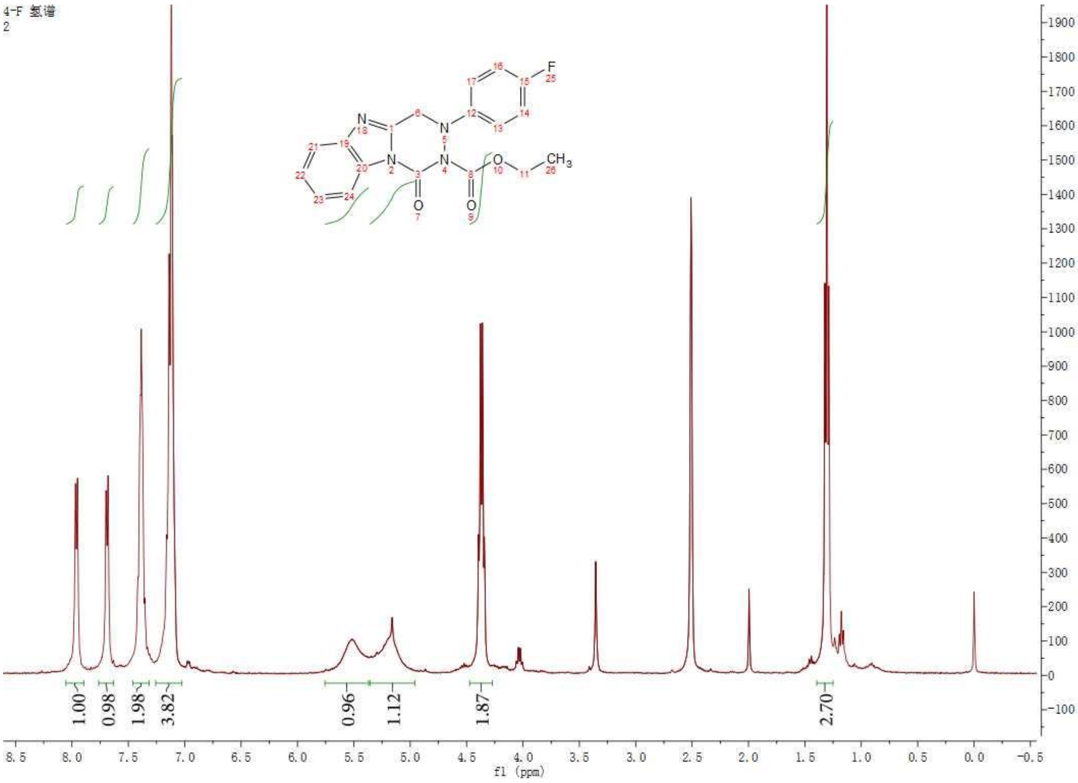

77. ^{13}C NMR spectra of compound **4t**

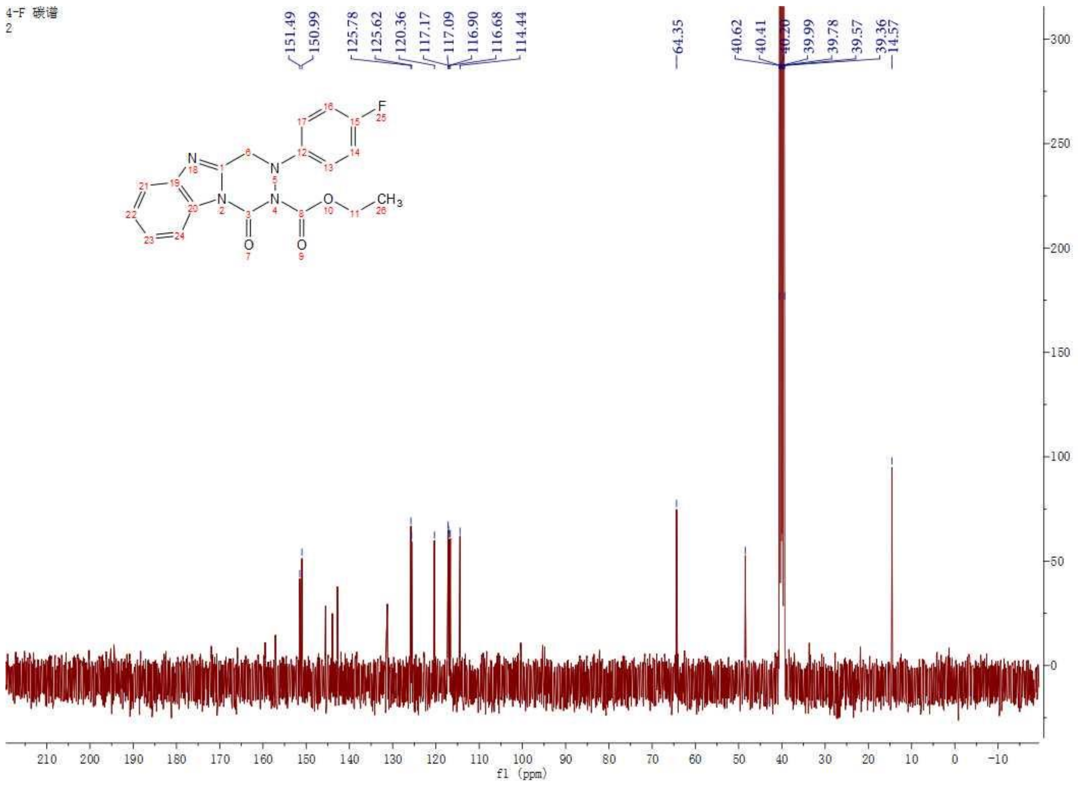

78. EI-MS spectra of compound **4t**

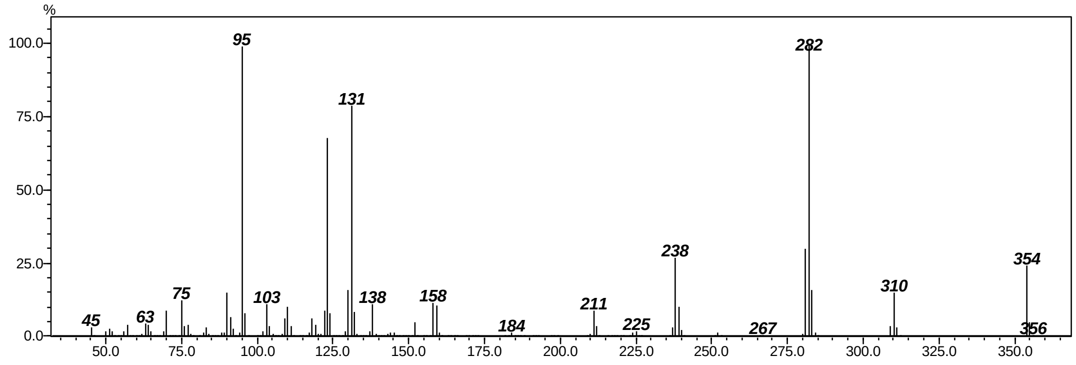

79. FT-IR spectra of compound **4t**

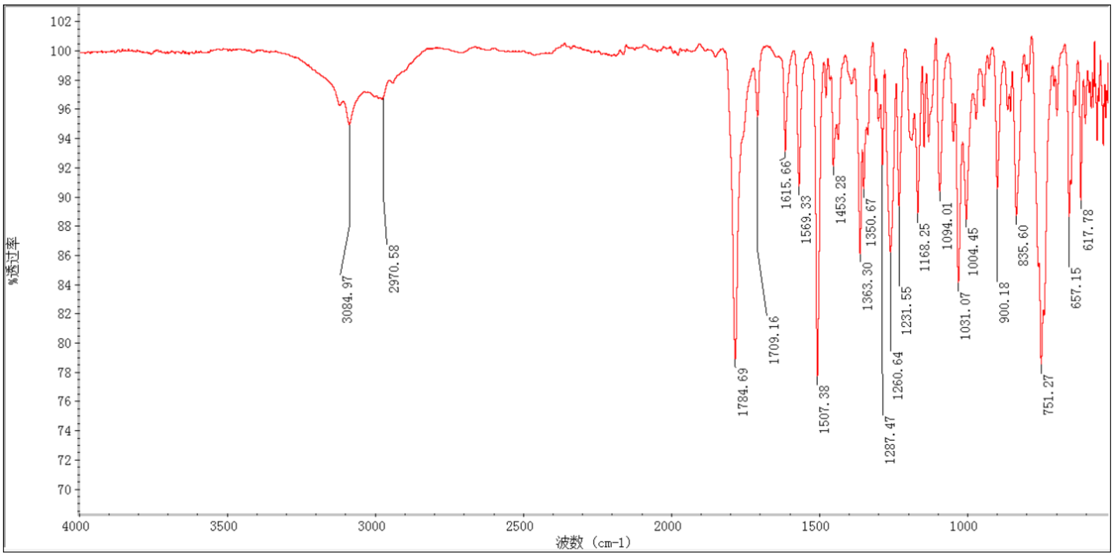

80. ^{1}H NMR spectra of compound **4u**

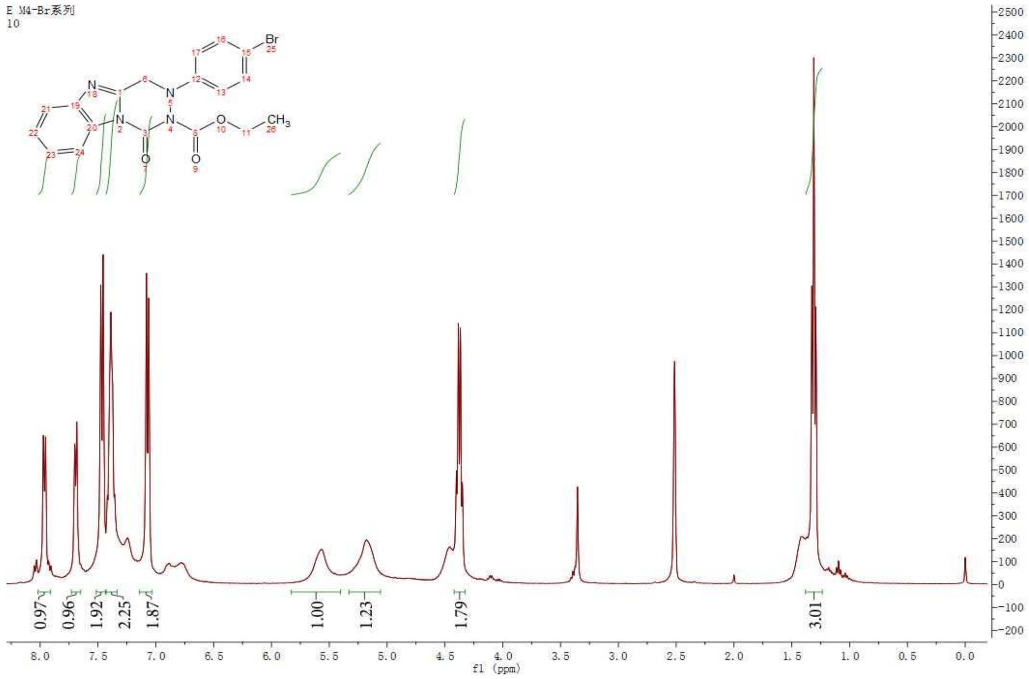

81. ^{13}C NMR spectra of compound **4u**

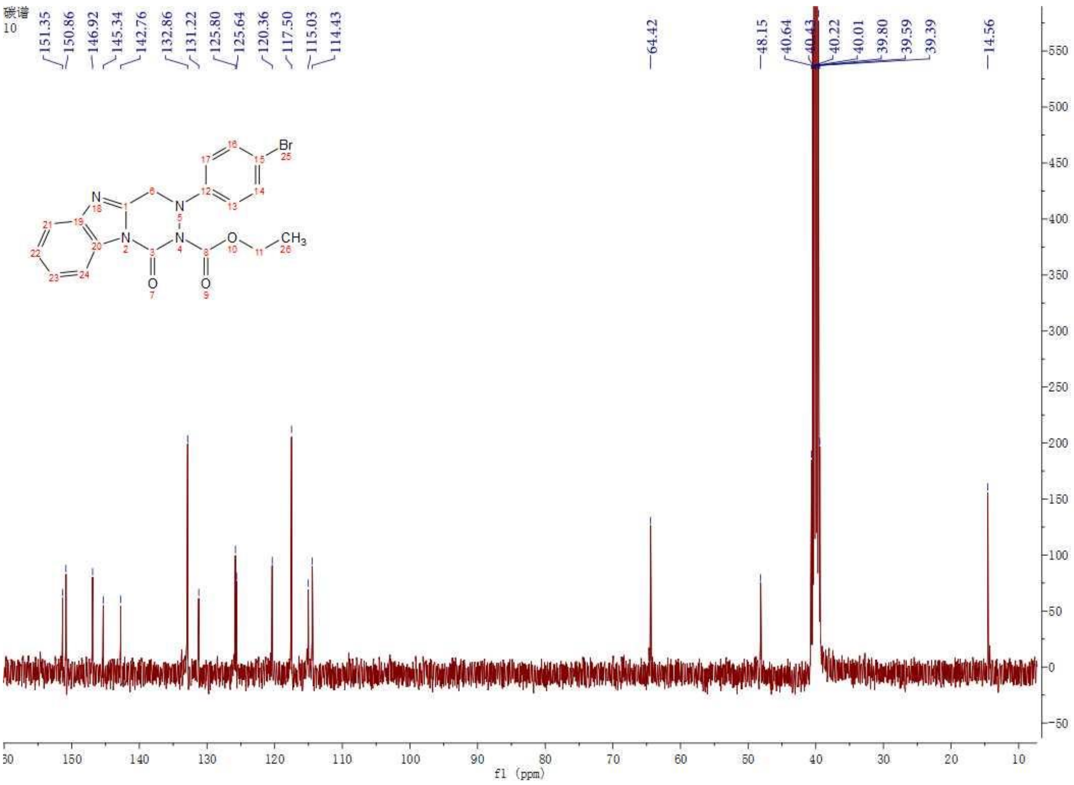

82. EI-MS spectra of compound **4u**

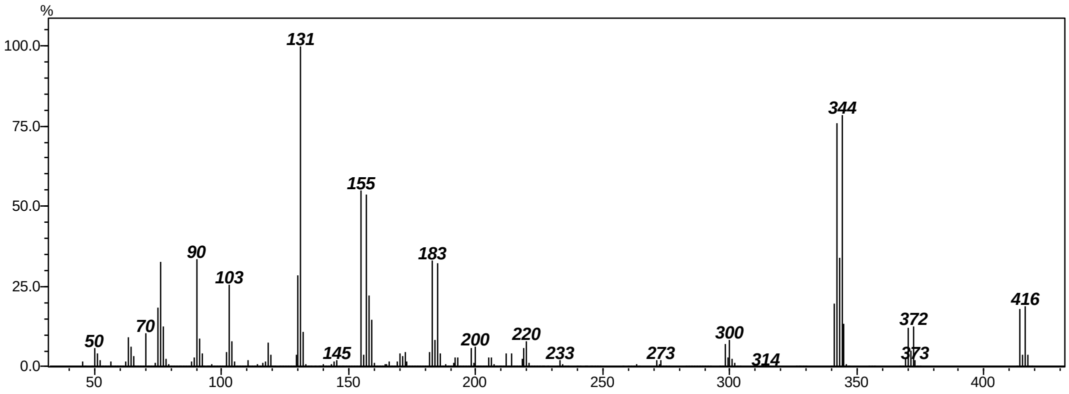

83. FT-IR spectra of compound **4u**

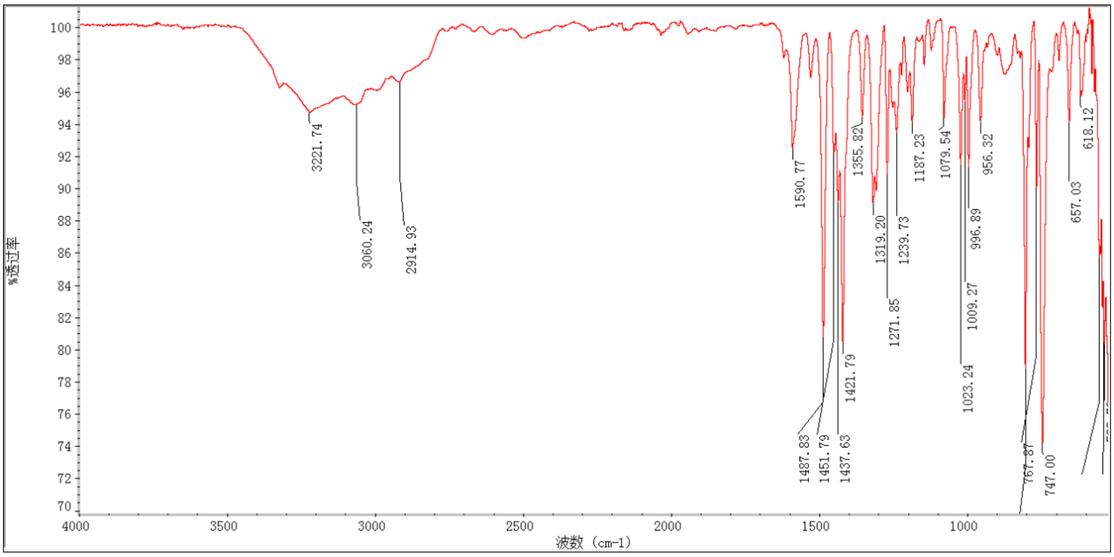

84. ^{1}H NMR spectra of compound **4v**

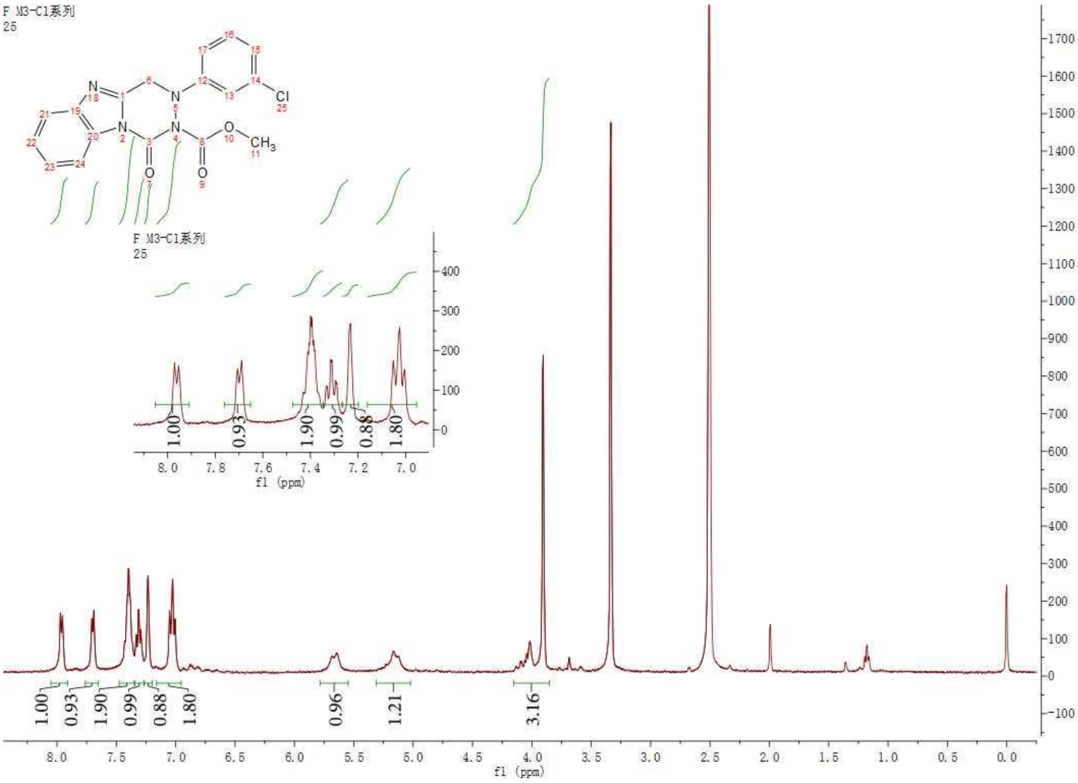

85. ^{13}C NMR spectra of compound **4v**

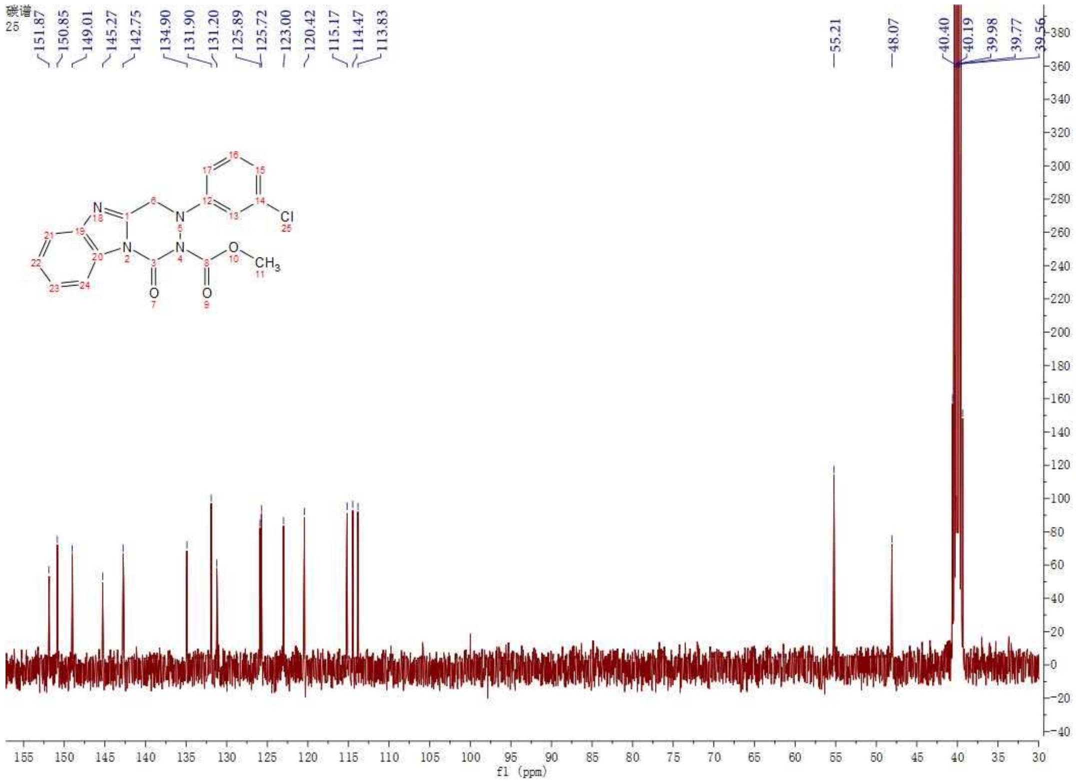

86. EI-MS spectra of compound **4v**

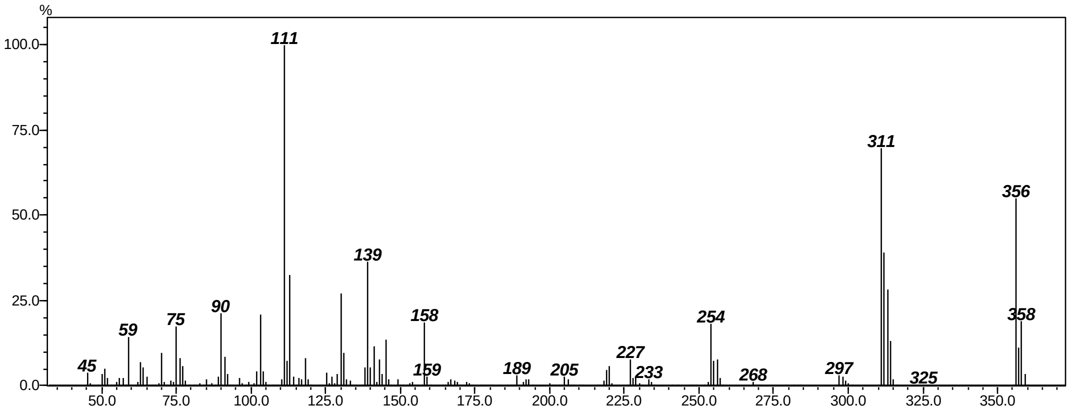

87. FT-IR spectra of compound **4v**

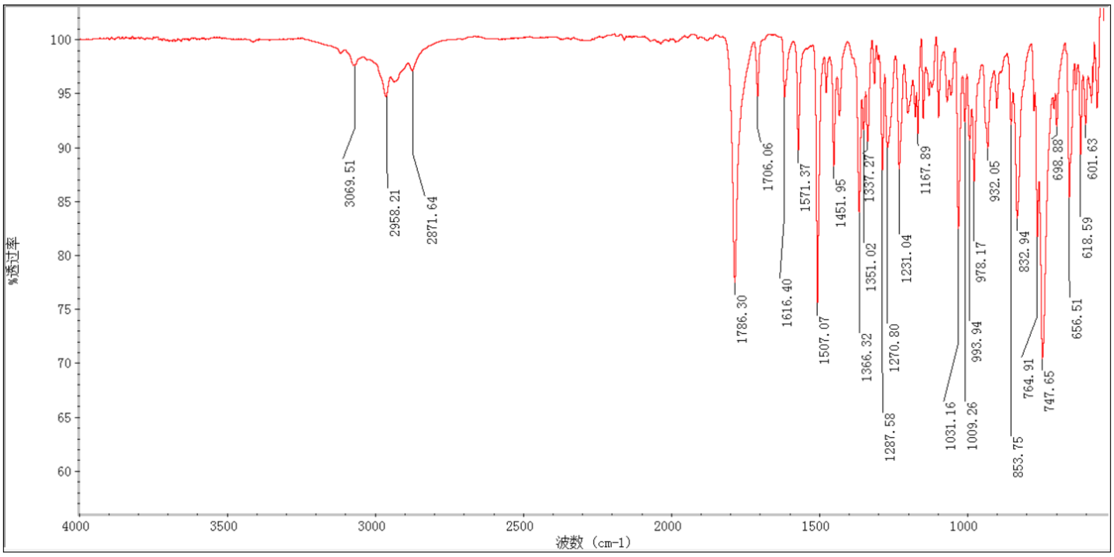

88. ¹H NMR spectra of compound **4w**

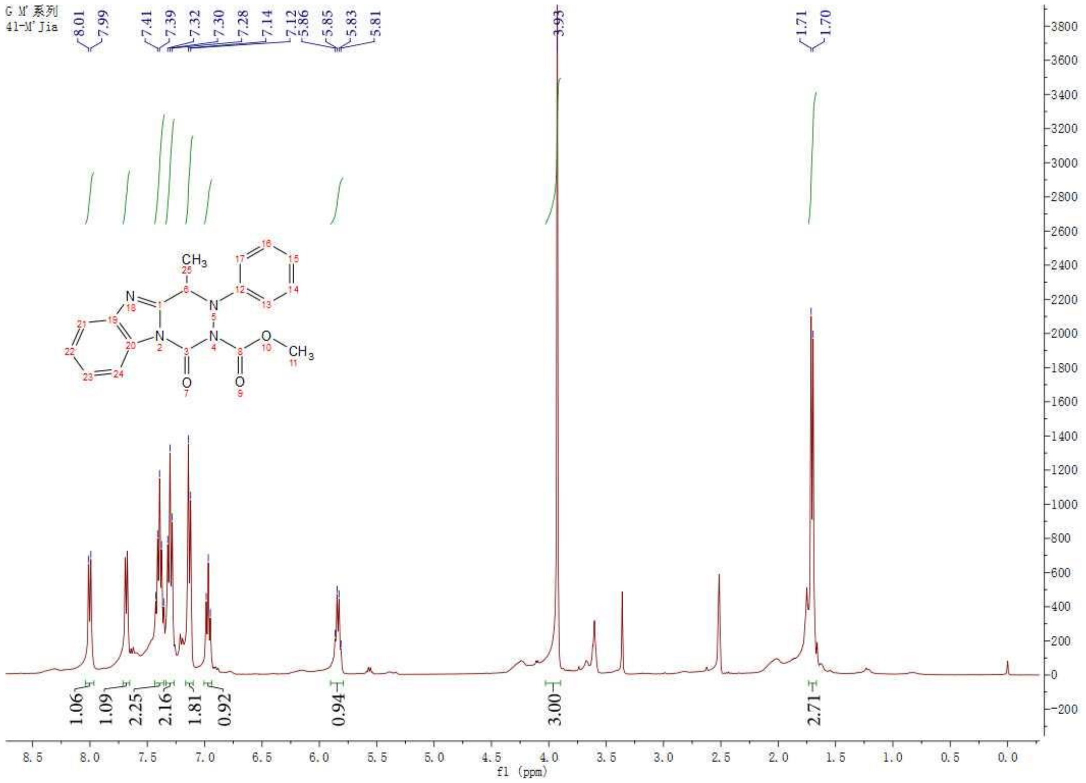

89. ^{13}C NMR spectra of compound **4w**

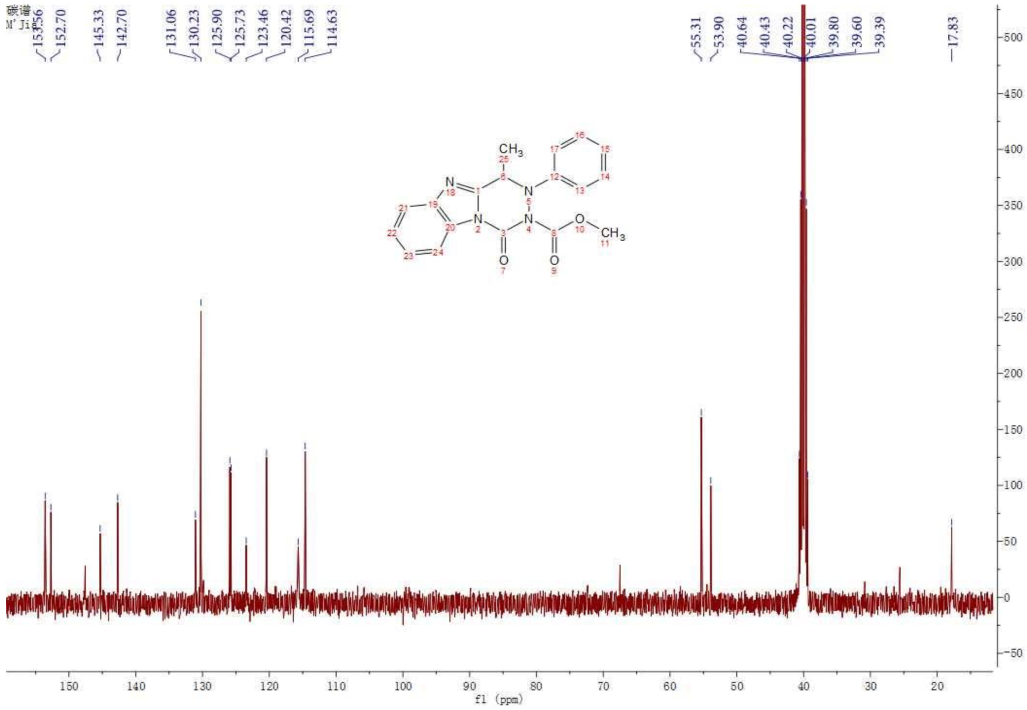

90. EI-MS spectra of compound **4w**

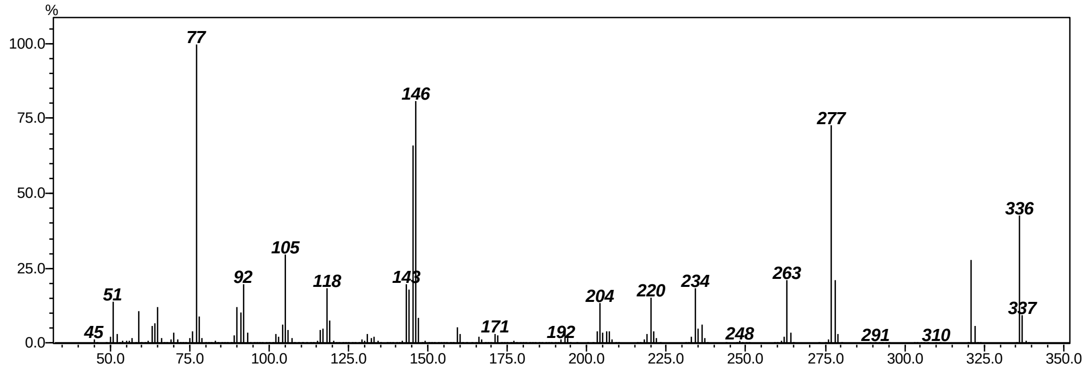

91. FT-IR spectra of compound **4w**

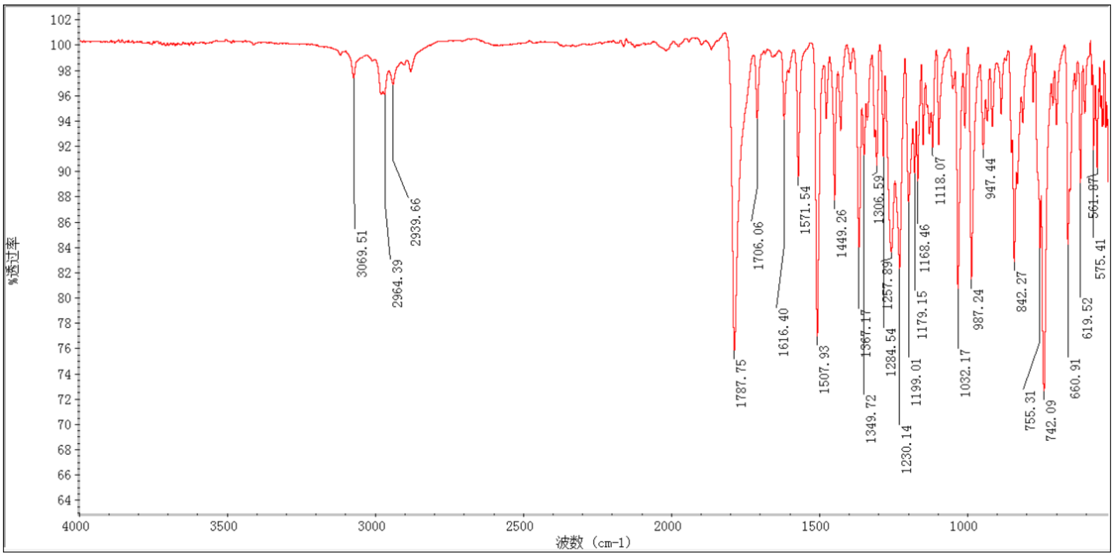

92. Selected geometric parameters of compound **4n**.

**Bond Lengths**			
**Bond**	**Dist. (Å)**	**Bond**	**Dist. (Å)**
O1–C9	1.202(2)	C1–C2	1.396(3)
O2–C10	1.190(2)	C1–C6	1.397(3)
O3–C10	1.327(2)	C2–C3	1.377(3)
O3–C11	1.460(3)	C3–C4	1.395(3)
N1–C1	1.410(2)	C4–C5	1.390(3)
N1–C7	1.293(2)	C5–C6	1.391(3)
N2–C6	1.406(2)	C7–C8	1.490(2)
N2–C7	1.401(2)	C12–C13	1.390(3)
N2–C9	1.402(2)	C12–C17	1.398(3)
N3–N4	1.423(2)	C13–C14	1.394(3)
N3–C8	1.471(2)	C14–C15	1.390(3)
N3–C12	1.440(2)	C15–C16	1.390(3)
N4–C9	1.408(2)	C16–C17	1.389(3)
N4–C10	1.417(2)	C15–C18	1.513(3)
**Bond Angles**			
**Angle**	**(°)**	**Angle**	**(°)**
C10–O3–C11	116.59(16)	N1–C7–N2	114.06(13)
C1–N1–C7	104.51(14)	N1–C7–C8	131.16(14)

C6–N2–C7	105.60(13)	N2–C7–C8	114.78(14)
C6–N2–C9	128.08(13)	N3–C8–C7	107.83(12)
C7–N2–C9	125.77(13)	O1–C9–N2	122.52(15)
N4–N3–C8	109.18(12)	O1–C9–N4	125.60(17)
N4–N3–C12	112.75(12)	N2–C9–N4	111.86(13)
C8–N3–C12	118.51(13)	O2–C10–O3	126.63(17)
N3–N4–C9	119.26(14)	O2–C10–N4	123.85(16)
N3–N4–C10	118.07(13)	O3–C10–N4	109.53(14)
C9–N4–C10	122.54(13)	N3–C12–C13	121.63(15)
N1–C1–C2	128.83(17)	N3–C12–C17	120.24(14)
N1–C1–C6	111.04(15)	C13–C12–C17	118.00(14)
C2–C1–C6	120.12(16)	C12–C13–C14	120.41(17)
C1–C2–C3	117.69(19)	C13–C14–C15	122.23(18)
C2–C3–C4	121.5(2)	C14–C15–C16	116.66(16)
C3–C4–C5	122.05(18)	C14–C15–C18	122.36(16)
C4–C5–C6	115.81(18)	C16–C15–C18	120.98(17)
N2–C6–C1	104.76(13)	C15–C16–C17	122.10(17)
N2–C6–C5	132.38(17)	C12–C17–C16	120.59(16)
C1–C6–C5	122.84(17)		

93. Hydrogen bonding data for compound **4n** (Å, °).

D–H···A	d(D–H)	d(H···A)	d(D···A)	∠(DHA)
C8–H8B···O3[a]	0.9700	2.500	3.385(3)	152.00
C17–H17···N4	0.9300	2.4500	2.761(3)	100

Symmetry code for compound **4n**: (a) -x,-y,-z.

94. Selected distances between hydrogen and carbon atoms for compound **4n** (Å).

**Bond**	**Dist. (Å)**	**Bond**	**Dist. (Å)**
H3···C12	3.4404(23)	H4···C12	3.5022(26)
H3···C13	3.8177(27)	H4···C13	3.1355(26)
H3···C14	3.8124(31)	H4···C14	3.1414(26)
H3···C15	3.4249(26)	H4···C15	3.5343(29)
H3···C16	2.9135(24)	H4···C16	3.8278(32)

H3···C17	2.8950(24)	H4···C17	3.8172(29)
